# TANK potentiates antiviral innate immunity by recruiting deubiquitinase USP46 to activate IKKε

**DOI:** 10.1371/journal.ppat.1014412

**Published:** 2026-07-10

**Authors:** Zhenghao Li, Can Yang, Juanjuan Shu, Jiaxin Wang, Xinyu Wang, Shasha Tan, Ji Liu, Jun Xiao, Jiaji Pan, Xing Feng, Hui Wu, Hao Feng

**Affiliations:** 1 College of Life Science, Hunan Normal University, Changsha, China; 2 Institute of Interdisciplinary Studies, Hunan Normal University, Changsha, China; 3 College of Engineering and Design, Hunan Normal University, Changsha, China; 4 Hunan Laboratory of Study and Discovery of Small Targeted Molecules of Hunan Province, School of Pharmaceutical Sciences, Hunan Normal University, Changsha, China; Shenzhen University, CHINA

## Abstract

IKKε plays an important role in interferon (IFN) production, however, its regulation remains poorly understood. In this study, we demonstrate that black carp TANK (bcTANK) interacts with bcIKKε and significantly enhances its activation of bcIRF3 and bcIRF7, leading to the improved antiviral activity against spring viremia of carp virus (SVCV). Mechanistically, bcTANK recruits deubiquitinase bcUSP46 to bcIKKε and leads to a reduction in polyubiquitination of and a concurrent increase in phosphorylation of bcIKKε, which suggests the activation status of this molecule. Site-directed mutagenesis revealed that lysine residues K418 and K545 of bcIKKε are critical for bcIKKε-mediated IFN production. Furthermore, we identified the SVCV phosphoprotein (P) as a viral antagonist of bcTANK/bcIKKε/bcIRF cascade. The SVCV P protein interacts with bcIKKε, promotes its polyubiquitination, attenuates its phosphorylation and disrupts its interaction with bcTANK, bcUSP46, bcIRF3, and bcIRF7, thereby facilitating viral replication. Thus, our findings reveal a novel positive regulation of IKKε by TANK.

## Author summary

The activation of IKKε during viral infection remains largely unknown. This study highlights the critical role of bcTANK in enhancing bcIKKε-mediated antiviral signaling against SVCV infection. We show that bcTANK potentiates host antiviral responses by modulating the phosphorylation and ubiquitination of bcIKKε by recruiting bcUSP46. Furthermore, SVCV P protein antagonizes this pathway by targeting bcIKKε, providing a new perspective on viral immune evasion strategies. This research offers insights into the molecular interactions that govern antiviral immunity in teleost fish and presents potential targets for antiviral strategies in aquaculture.

## Introduction

Viral pathogens pose a continuous threat to their hosts, necessitating the rapid deployment of an effective innate immune response to restrict replication before adaptive immunity develops [1]. A central component of this first line of defense is the recognition of viral nucleic acids by host pattern recognition receptors (PRRs). Among these, RIG-I-like receptors (RLRs) are specialized in detecting RNA viruses and initiating signaling cascades that culminate in the production of type I IFNs and the induction of a broad antiviral state [2–6]. The core signaling machinery that translates RLR activation into IFN production centers on the non-canonical IκB kinases, TANK-binding kinase 1 (TBK1) and inducible IκB kinase (IKKε), which phosphorylate and activate IFN regulatory factor 3 (IRF3) and IRF7 [7–9]. Despite the well-characterized role of TBK1, the regulatory mechanisms that govern IKKε activation and its downstream effects remain incompletely understood. Elucidating how IKKε is activated upon viral detection, as well as how viruses may evolve strategies to disrupt its function, is a critical question for understanding host-pathogen interactions and immune evasion.

IKKε and TBK1 are members of the IKK family and are classified as non-canonical IKKs [10,11]. Their structures are highly similar, comprising an N-terminal kinase domain (KD), a ubiquitin-like domain (ULD), a scaffold-dimerization domain (SDD/CCD1), and a coiled-coil domain 2 (CCD2) [12,13]. Both IKKε and TBK1 play crucial roles in multiple signaling pathways, including those involving IFN and NF-κB activation [14,15]. Initially, IKKε was identified as an LPS-induced gene [16]. Later studies revealed that it functions similarly to TBK1 in regulating IFN production. Upon activation of upstream receptors such as TLRs, RLRs, or cGAS, these pathways initiate cascades that ultimately activate IRF3/7 and NF-κB, promoting their nuclear translocation and functional activation [15,17–19]. The post-translational modification (PTM) and precise mechanism that control IKKε activity, particularly in response to viral infection, still need further investigation.

A key regulator of the TBK1/IKKε complex is the scaffold protein TANK (TRAF family member-associated NF-κB activator), which was initially identified as a TRAF-binding protein involved in immune signaling [7]. TANK knockdown impairs TNFα-induced NF-κB activation [20,21], whereas IKKβ-mediated phosphorylation of TANK under TNFα stimulation reduces its interaction with NEMO, thereby inhibiting NF-κB signaling [22]. TANK itself is subjected to multiple forms of post-translational regulation, including phosphorylation, ubiquitination, and SUMOylation, which fine-tune its activity [8,23]. Recent studies have demonstrated that TANK functions as a scaffold protein to assemble the TBK1/IKKε complex and to promote IRF3/7 activation. Although substantial evidence supports a role for TANK in IFN pathways, its functional mechanisms in teleost fish, particularly in relation to IKKε, remain incompletely characterized.

The intricate host defense network is inevitably countered by viral evasion strategies. Many viruses encode proteins that directly target key components of the IFN pathway to facilitate immune evasion [24–28]. Spring viremia of carp virus (SVCV), a negative-sense RNA virus and a major pathogen in freshwater aquaculture, provides a compelling model to study these interactions in teleost fish [29–31]. Previous reports indicate that SVCV proteins can interfere with host immunity, but the molecular targets and mechanisms, especially concerning the IKKε axis, remain largely unexplored [32,33]. Thus, SVCV represents both a significant economic threat and a valuable model system for investigating the mechanisms underlying the interactions between aquatic viruses and their hosts [34–36].

In our previous studies, we cloned and characterized IKKε and TANK from black carp (bcIKKε and bcTANK), demonstrating their involvement in the antiviral response against SVCV [37–39]. To further elucidate the roles of bcTANK and bcIKKε in innate antiviral immunity, we conducted a detailed investigation into the regulatory mechanisms underlying their functions. We found that bcTANK recruits bcUSP46 to bcIKKε, thereby facilitating the removal of polyubiquitin chains, enhancing serine phosphorylation, and promoting bcIKKε activation. We also identified the SVCV P protein as a viral antagonist of this process. The SVCV P protein binds to bcIKKε, promotes its polyubiquitination, suppresses its phosphorylation, and disrupts its interaction with bcTANK and bcIRF3/7, thereby compromising the host’s immune response and facilitating viral replication. Our findings illuminate a complete cycle of host defense and viral countermeasures, centered on the dynamic ubiquitination of bcIKKε, and provide deep insight into the molecular interplay between teleost fish and RNA viruses.

## Materials and methods

### Ethics statement

Fish experiments in this study were conducted in accordance with institutional guidelines and approved by the biomedical ethics committee of Hunan Normal University (Approval No. 2024195).

### Cell culture and transfection

Human embryonic kidney (HEK) 293T cells, Epithelioma Papulosum Cyprinid (EPC) cells, black carp kidney (MPK) cells, and black carp caudal fin (MPF) cells were kept in the laboratory. EPC, MPF, and MPK cells were cultured at 26 °C with 5% CO_2_, whereas HEK293T cells were cultured at 37 °C with 5% CO_2_. All cell lines were maintained in Dulbecco’s Modified Eagle Medium (DMEM) (BaseIMedia, China) containing 10% fetal bovine serum, 100 U/mL penicillin, and 100 μg/mL streptomycin. Polyethylenimine (PEI) (Yeasen, China) was used for cell transfection according to the manufacturer’s instructions.

To establish stable eUSP46 knockdown cell lines, EPC cells were seeded in 6-well plates and transfected with pLKO-eUSP46-shRNA or pLKO-scramble control plasmids. After 48 hours of transfection, cells were selected with complete DMEM medium containing puromycin (1 mg/mL). Stable eUSP46 knockdown cell line was established when all control EPC cells without transfection had died.

### Plasmids and reagents

*bcTANK* and *bcIKKε* genes were amplified by PCR from MPK cell cDNA. Other plasmids, including bcIRF3, bcIRF7, SVCV-M, SVCV-G, SVCV-P, SVCV-N, HA-Ub, pRL-TK, Luci-DrIFNφ3 (for zebrafish IFNφ3 promoter transcription analysis), and Luci-bcIFNa (for black carp IFNa promoter transcription analysis) were previously established in our laboratory.

### Virus infection and plaque assay

SVCV (strain 741) was propagated in EPC cells. When cytopathic effect (CPE) reached approximately 50% at 2–3 days post-infection, both cells and supernatant were collected. After three freeze-thaw cycles, the lysate was filtered through a 0.45 μm membrane, and the virus stock was stored at -80 °C. For plaque assays, EPC cells were infected with SVCV at the indicated MOI. The supernatant was collected 1–2 h after viral infection. Subsequently, untreated EPC cells were infected with serial dilutions of the viral supernatant. After 1–2 h, the viral supernatant was replaced with the semi-solid medium containing methylcellulose and 2% FBS. Plaques were counted following 2–3 days of incubation after crystal violet staining.

### Quantitative real-time PCR (qRT-PCR)

Total RNA was extracted using the Rapure Total RNA Plus Kit (Magen, China). cDNA was synthesized using the PrimeScript FAST RT reagent Kit with gDNA Eraser (Takara Bio, Japan). qRT-PCR was performed using ChamQ Universal SYBR qPCR Master Mix (Vazyme, China) in the following program: 1 cycle of 95 °C/10 min, 40 cycles of 95 °C/15 s, 60 °C/1 min. Gene expression levels were calculated using the 2^-△△Ct^ method, with β-actin serving as the internal control.

### Dual-luciferase reporter assay

Cells in 24-well plates were co-transfected with expression plasmids (300 ng), pRL-TK (25 ng) and Luci-bcIFNa/Luci-DrIFNφ3 (200 ng) for 24 h. Cells were then collected and lysed with Passive Lysis Buffer (PLB). Luciferase activity was measured using the Dual-Luciferase Reporter Assay System (Promega, USA), according to the manufacturer’s protocol.

### Immunofluorescence

Cells in 24-well plates were co-transfected with recombinant expression plasmids (500 ng/well) for 24 h. Cells were subsequently fixed with 4% paraformaldehyde for 10 min. The cells were immediately permeabilized with 0.2% Triton X-100 for 10 min and blocked with goat serum for 1 h. Next, the cells were sequentially incubated with indicated primary antibodies (1:500, Abmart, China) and secondary antibodies (1:1000, Invitrogen, USA). Finally, the cells were washed three times with PBS. Nuclei were stained with of DAPI (Vector Laboratories, USA). Images were captured using a laser confocal microscope (Olympus FV1200, Japan).

### Co-immunoprecipitation (Co-IP) and western blot assay

Cells in 10 cm dishes were co-transfected with indicated plasmids (15 μg/dish) and were washed with PBS, then lysed by sonication with 1% NP40 and centrifuged to obtain whole-cell lysate. Whole-cell lysates were incubated with anti-HA-conjugated agarose beads (Sigma, USA) or anti-Flag-conjugated agarose beads (Sigma, USA) at 4 °C overnight. The next day, the agarose beads were washed five times with 1% NP40 lysis buffer. Then the agarose beads were resuspended in SDS loading buffer for immunoblotting (IB). The prepared samples were separated by 10% SDS-PAGE, and the proteins were subsequently transferred to 0.45 μm PVDF membranes. The membranes were blocked by immersion in 5% skimmed milk and subsequently incubated with primary antibodies (1:5000, Abmart, China) at 4°C overnight. The membranes were washed four times with TBST and then incubated with secondary antibodies (1:30000, Sigma, USA) for 1 h at room temperature. Protein bands were visualized using the BCIP/NBT Alkaline Phosphatase Color Development Kit (Sigma, USA).

### shRNA knockdown assay

shRNAs targeting bcTANK were designed using Thermo Fisher’s BLOCK-iT RNAi-Designer website. The designed shRNA sequences were synthesized and inserted into the pLKO.1 plasmid vector. The constructed shRNA plasmids were then transiently transfected into cells and knockdown efficiency was evaluated by qRT-PCR.

### Statistical analysis

The statistical analysis was performed using GraphPad Prism version 10.0. Data are presented as the mean ± SEM from three independent experiments. Comparisons between two groups were performed using a two-tailed Student’s t-test, with the following significance levels: ^*^, P < 0.05; ^**^, P < 0.01.

## Results

### Involvement of bcTANK in the antiviral innate immune response

**Fig 1:**
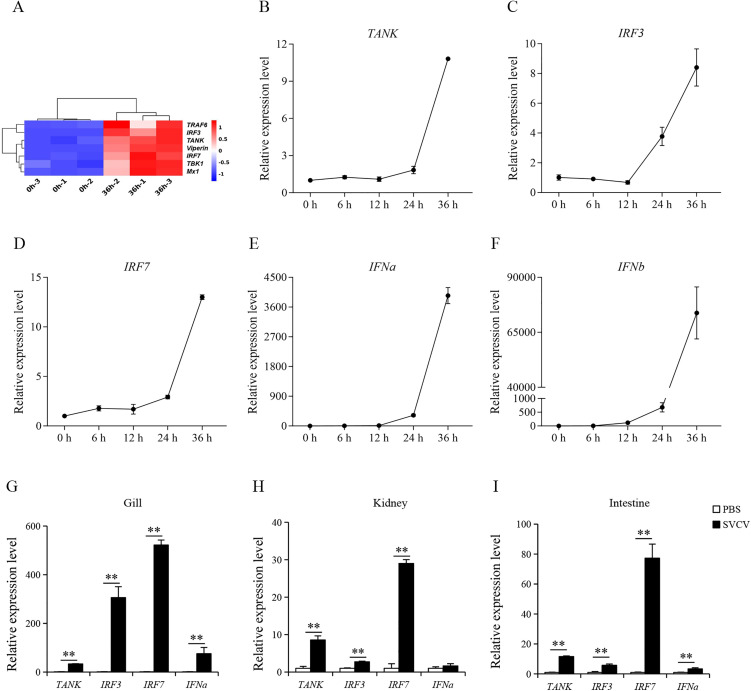
The involvement of bcTANK in host antiviral innate immune response. **(A)** Heatmap showing transcriptional variations of *bcTANK*, *bcTRAF6*, *bcTBK1*, *bcIRF3*, *bcIRF7*, and *bcISG*s in MPF cells either mock-treated or infected with SVCV (MOI = 0.1) for 36 hours. (B-F) qRT-PCR analysis of *bcTANK* **(B)**, *bcIRF3* **(C)**, *bcIRF7* **(D)**, *bcIFNa* (E) and *bcIFNb* (F) mRNA levels in MPF cells infected with SVCV (MOI = 0.1) at 0, 6, 12, 24, and 36 h post infection. Data represented the means ± SEM (n = 3). (G-I) Juvenile black carp were infected with either PBS or SVCV (2 × 10^6^ copies/mL) (MOI = 0.1), and indicated tissues were harvested after 3 days infection and used for qRT-PCR detection.

Based on the transcriptome data from MPF cells infected with SVCV, we analyzed the transcriptional levels of selected genes, including *bcTANK*, *bcTRAF6*, *bcTBK1*, *bcIRF3*, *bcIRF7*, and *IFN stimulated genes* (*ISGs*). The analysis revealed a notable increase in *bcTANK* mRNA expression at 36 hours post-SVCV infection. Similarly, the mRNA levels of other key antiviral signaling components, including *bcTRAF6*, *bcTBK1*, *bcIRF3*, *bcIRF7*, *bcViperin*, and *bcMX1*, also exhibited significant upregulation (Fig 1A). Furthermore, qRT-PCR validation confirmed that *bcTANK* expression began to gradually increase at 12 hours after SVCV stimulation, peaking at 36 hours post-infection 10.84 times higher than the control group (Fig 1B). Concurrently, the expression of two key IFN regulatory factors, *bcIRF3* and *bcIRF7*, as well as type I IFNs such as *bcIFNa* and *bcIFNb* (Fig 1C-1F), followed a similar upward trend to that of *bcTANK*. Subsequently, juvenile black carp were infected with SVCV or injected with PBS as control. Following SVCV infection, the expression levels of *bcTANK*, *bcIRF3*, *bcIRF7*, and *bcIFNa* were significantly upregulated in the examined tissues, including gill, kidney, and intestine (Fig 1G-1I). These data collectively indicated that bcTANK participated in host innate immune response to SVCV infection.

### Knockdown of bcTANK impaired the antiviral activity of host

**Fig 2:**
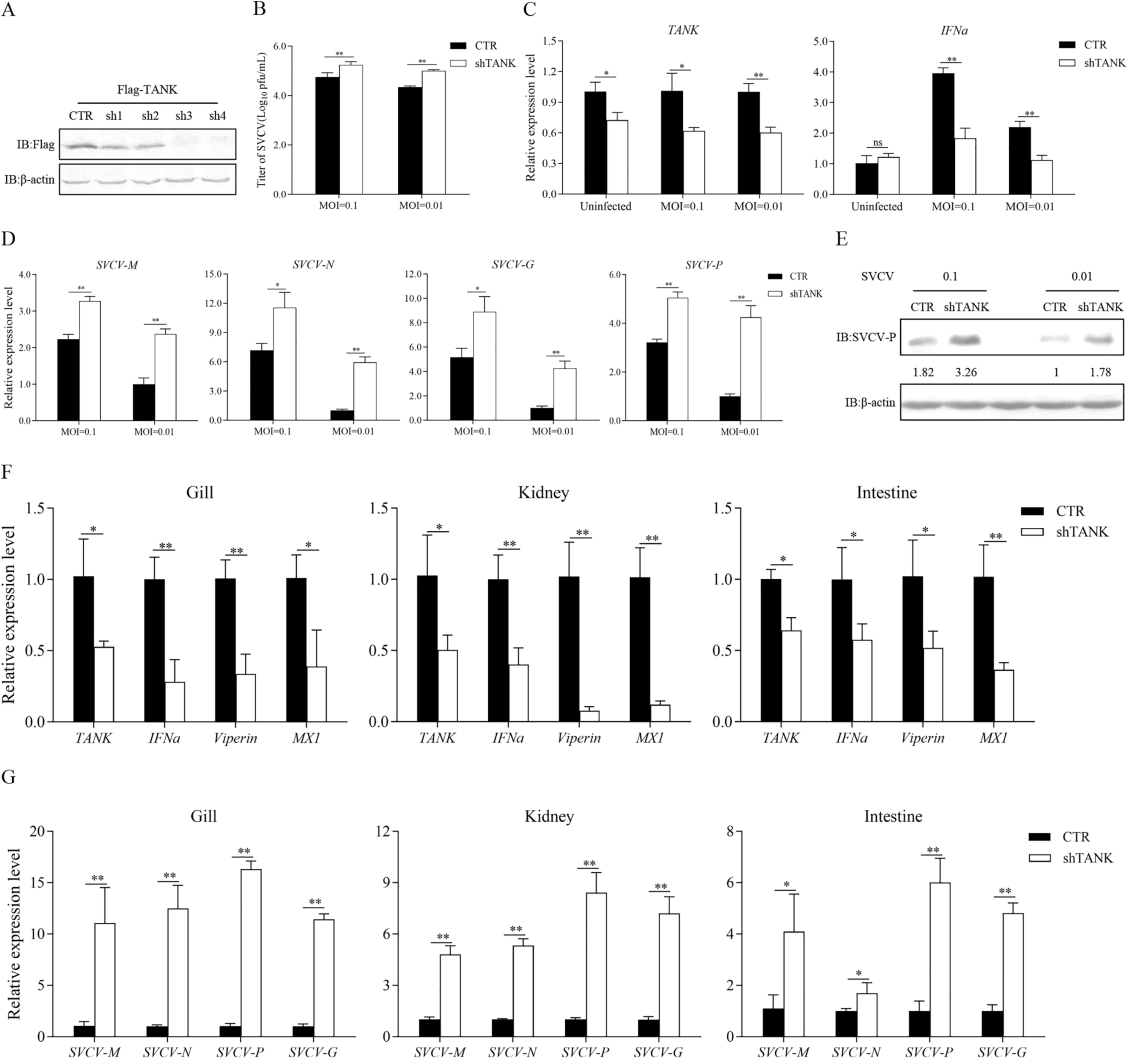
Knockdown of bcTANK impairs the antiviral activity of host cells. **(A)** Western blot analysis of bcTANK expression in HEK293T cells co-transfected with Flag-bcTANK (1.5 μg/well) and control scramble shRNA (1.5 μg/well) or one of four independent bcTANK-targeting shRNAs (sh1-sh4) (1.5 μg/well). **(B)** MPK cells in 6-well plates were transfected with shbcTANK (sh-3) (3 μg/well) or shscramble (CTR) (3 μg/well) and infected with SVCV at the indicated MOIs for 24 hours. Then, viral titers in the supernatants from MPK cells were measured. (C-D) qRT-PCR analysis of *bcTANK*, *bcIFNa* **(C)**, and *SVCV* (D) mRNA levels in the cells from **(B)**. Data represented the means ± SEM (n = 3) and were tested for statistical significance using a two-tailed Student’s t-test. **P < 0.01. **(E)** SVCV P protein levels were measured in the cells from **(B)**. **(F-G)** Black carp were intramuscularly injected with shbcTANK (sh-3) or control scramble shRNA at a dosage of 1 μg plasmid per gram of body weight. Three days after injection, the fish were challenged with either PBS or SVCV (2 × 10^6^ copies/mL). At 3 days post-infection, gill, kidney, and intestine tissues were harvested for qRT-PCR analysis **(F-G)**. Relative mRNA expression levels of *bcTANK*, *bcIFNa*, *bcViperin*, and *bcMX1* in gill, kidney, and intestine from the bcTANK knockdown (sh-3) and scramble control groups prior to viral infection **(F)**. Relative mRNA expression levels of *SVCV-M*, *SVCV-N*, *SVCV-P,* and *SVCV-G* in the same tissues after SVCV infection **(G)**.

To investigate the role of bcTANK in antiviral immunity, we generated four shRNA plasmids targeting bcTANK (sh-1, sh-2, sh-3, and sh-4) and co-transfected them with the Flag-bcTANK expression plasmids into HEK293T cells. Western blot analysis confirmed that both plasmids sh-3 and sh-4 possessed high knockdown efficiency (Fig 2A). Next, MPK cells were transfected with bcTANK (sh-3) or scramble shRNA, and subsequently infected with SVCV at two different multiplicities of infection (MOIs) of 0.1 and 0.01. The viral titers in the supernatants of bcTANK-knockdown group were significantly higher compared to the control group, suggesting that bcTANK-knockdown enhanced viral replication (Fig 2B). qRT-PCR further confirmed the knockdown efficiency, showing a marked decrease in *bcTANK* mRNA levels in both uninfected or infected cells, as well as a significant reduction in *bcIFNa* expression in infected cells (Fig 2C). In addition, the expression levels of the viral genes *SVCV-M*, *-N*, *-P* and *-G* were upregulated in bcTANK-knockdown cells, indicating increased viral replication (Fig 2D). Consistently, SVCV-P protein levels were also increased in bcTANK-knockdown cells at both MOIs of 0.1 and 0.01 (Fig 2E). To further explore the impact of bcTANK knockdown on viral replication *in vivo*, juvenile black carp were injected with shRNA plasmids targeting *bcTANK* or the control scramble shRNA independently, then infected with SVCV. Significant reductions in *bcTANK*, *bcIFNa*, *bcViperin* and *bcMX1* expression were observed in the gill, kidney, and intestine of the bcTANK-knockdown group compared with the scramble group, indicating that bcTANK knockdown impaired basal antiviral defense mechanisms in these tissues (Fig 2F). Subsequent analysis revealed a marked increase of the viral genes *SVCV-M*, *-N*, *-P* and *-G* in the gill, kidney, and intestine of the bcTANK-knockdown group infected with SVCV (Fig 2G), and the increased viral titer was also detected in the gill of the bcTANK-knockdown group (S1 Fig), indicating that the antiviral response in these tissues was compromised due to bcTANK knockdown, leading to enhanced viral replication. Collectively, these findings highlighted the critical role of bcTANK in regulating antiviral responses both *in vivo* and *in vitro*, particularly in the modulation of the IFN pathway and viral control during SVCV infection.

### bcTANK enhanced bcIKKε-mediated antiviral ability

**Fig 3:**
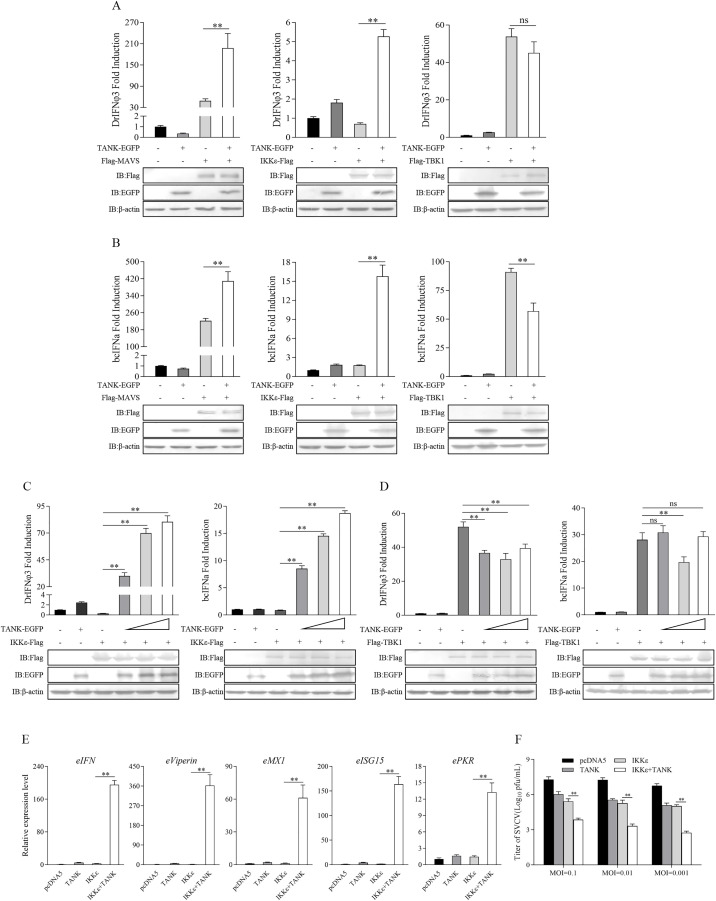
bcTANK enhances bcIKKε-mediated antiviral activity. **(A&B)** EPC cells in 24-well plates were co-transfected with bcMAVS (200 ng/well), bcIKKε (200 ng/well), bcTBK1 (200 ng/well), bcTANK (200 ng/well), Luci-bcIFNa (Luci-DrIFNφ3) (250 ng/well), or pRL-TK (25 ng/well). IFN promoter activity of DrIFNφ3 (A) or bcIFNa (B) was analyzed by dual-luciferase reporter assay. The protein expression levels of the corresponding plasmids were detected by western blotting assay. **(C&D)** Dose-dependent effects of bcTANK on (C) bcIKKε- or (D) bcTBK1-mediated DrIFNφ3 or bcIFNa promoter activation. The protein expression levels of the corresponding plasmids were detected by western blotting assay. (E) qRT-PCR analysis of *eIFN*, *eViperin*, *eMX1*, *eISG15*, and *ePKR* expression in EPC cells transfected as indicated (500 ng plasmids/well). **(F)** Viral titers in supernatants from EPC cells transfected as indicated (500 ng/well) and infected with SVCV at MOIs of 0.1, 0.01, and 0.001. Data represented the means ± SEM (n = 3) and were tested for statistical significance using a two-tailed Student’s t-test. ns, P > 0.05. *P < 0.05. **P < 0.01.

To investigate the molecular mechanisms through which bcTANK influenced the IFN antiviral signaling, we co-transfected plasmids expressing bcMAVS, bcIKKε, or bcTBK1 with bcTANK into EPC cells and assessed IFN promoter activity using a dual-luciferase reporter assay. The results demonstrated that bcTANK significantly enhanced the DrIFNφ3 and bcIFNa promoter transcription induced by both bcMAVS and bcIKKε; however, no significant enhancement was observed in bcTBK1/bcTANK co-transfection group (Fig 3A and 3B). Furthermore, varying the amount of bcTANK plasmid revealed that bcTANK potentiated the IFN-inducing activity of bcIKKε, while it did not amplify the effect of bcTBK1 (Fig 3C and 3D). Besides, the qRT-PCR results indicated that co-transfection of bcTANK and bcIKKε significantly enhanced the mRNA levels of key antiviral factors, including *eIFN*, *eViperin*, *eMX1*, *eISG15*, and *ePKR*, compared to those of the cells expressing bcIKKε alone (Fig 3E). To further confirm the role of bcTANK in regulating bcIKKε-mediated antiviral activity, EPC cells over-expressing bcTANK and/or bcIKKε were infected with SVCV at MOIs of 0.1, 0.01, and 0.001. Viral titer assays revealed that SVCV titers from the supernatants of the cells co-expressing bcTANK and bcIKKε were significantly lower than those of the cells expressing bcIKKε alone (Fig 3F). However, co-expression of bcTANK with bcTBK1 did not result in a significant reduction in viral titers compared to the control group expressing bcTBK1 alone (S2 Fig). These results indicated that bcTANK enhanced host cell antiviral capacity through bcIKKε rather than bcTBK1.

### bcTANK facilitated bcIKKε self-assembly and promoted IRF3/IRF7 activation

**Fig 4:**
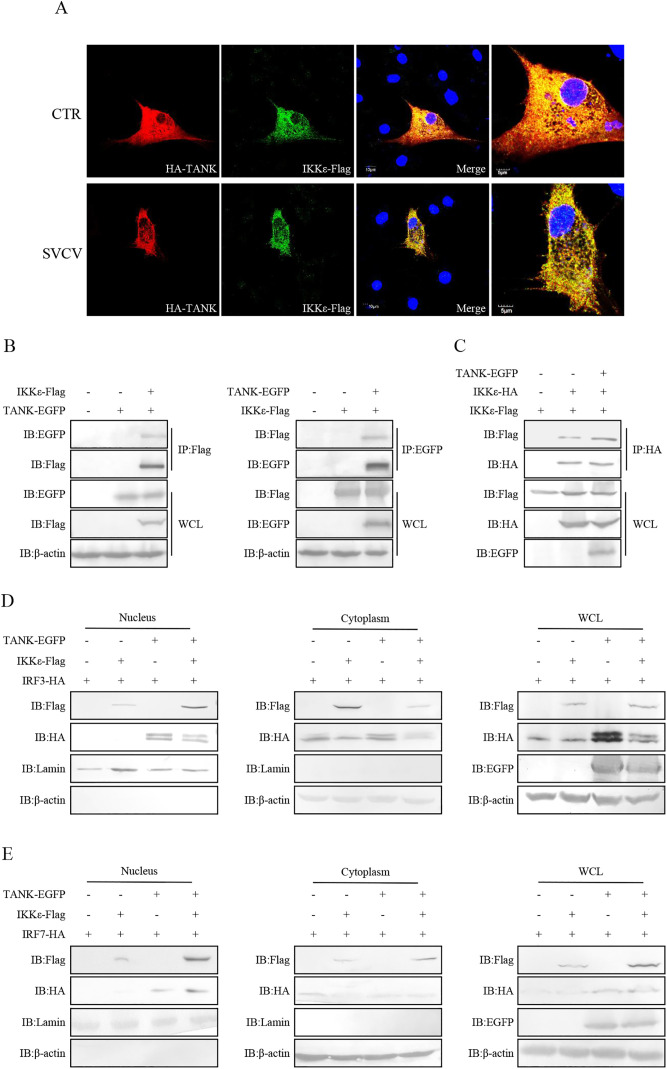
bcTANK interacts with bcIKKε and promotes downstream signaling activation. **(A)** MPK cells in 24-well plates were co-transfected with bcTANK (250 ng/well) and bcIKKε (250 ng/well) and infected with SVCV at MOI of 0.1 for 6 **h.** Immunofluorescence was used to detect colocalization: bcTANK (red), bcIKKε (green), merged signals (yellow), and nuclei (blue, DAPI). **(B)** HEK293T cells in 10 cm dishes were transfected with bcIKKε-Flag (7.5 μg/dish), and bcTANK-EGFP (7.5 μg/dish) as indicated, followed by co-IP. **(C)** HEK293T cells in 10 cm dishes were transfected with bcIKKε-Flag (5 μg/dish), bcIKKε-HA (5 μg/dish), and bcTANK-EGFP (5 μg/dish) as indicated, followed by co-IP. **(D&E)** Recombinant plasmids (3μg/well) shown in the figure were overexpressed in HEK293T cells. Nuclear and cytoplasmic fractions from cells transfected with the indicated plasmids were analyzed by western blot for bcIRF3 (D) and bcIRF7 (E) localization. Lamin and β-actin served as nuclear and cytoplasmic markers, respectively.

To investigate the association between bcTANK and bcIKKε, immunofluorescence and co-immunoprecipitation assays were conducted. As shown in Fig 4A, before and after SVCV infection, bcTANK and bcIKKε in MPK cells were predominantly localized in the cytoplasm and exhibited clear colocalization. Furthermore, co-IP results identified the interaction between bcTANK and bcIKKε in HEK293T cells (Fig 4B), as well as in EPC cells (S3 Fig). It has been reported that activated IKKε can undergo self-assembly, thereby promoting the nuclear translocation and activation of downstream transcription factors IRF3 and IRF7 [9,14,15,20,23,37,40]. The co-IP results revealed that co-expression of bcTANK and bcIKKε significantly enhanced the self-assembly capacity of bcIKKε (Fig 4C). Subsequent nuclear-cytoplasmic fractionation combined with western blot analyses revealed that no nuclear bcIRF3 was detected in the group of bcIRF3 expressing alone and co-expression of bcIKKε with bcIRF3. However, when co-expressed with bcTANK, bcIRF3 was detected in the nuclear fraction (Fig 4D). Co-expression of bcTANK with bcIRF7 led to partial nuclear localization of bcIRF7, which was significantly strengthened in the presence of bcIKKε (Fig 4E). Collectively, these results demonstrated that bcIKKε triggered nuclear translocation of bcIRF3 and bcIRF7, and bcTANK acted as a positive regulator that further potentiated bcIKKε-promoted bcIRF3/7 activation.

### bcTANK attenuated the ubiquitination and enhanced the phosphorylation of bcIKKε

**Fig 5:**
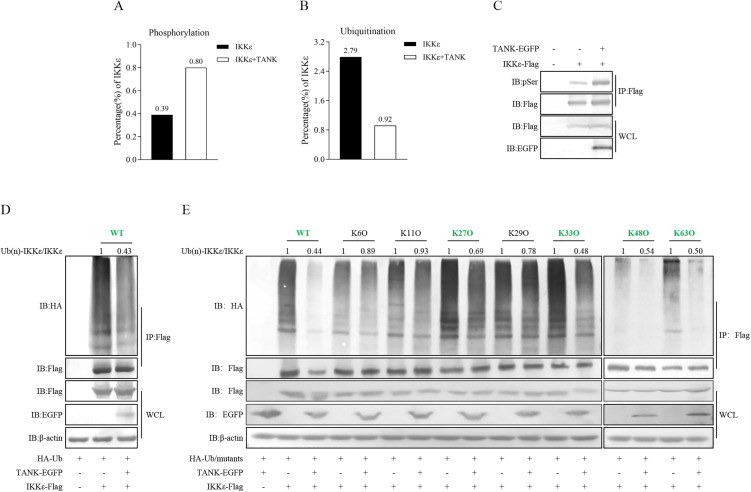
bcTANK enhances bcIKKε phosphorylation and reduces its ubiquitination. **(A&B)** Mass spectrometry analysis of immunoprecipitated bcIKKε from cells transfected with bcIKKε alone or co-transfected with bcIKKε and bcTANK (15 μg/dish). Relative phosphorylation (A) and ubiquitination (B) ratios of bcIKKε were quantified. **(C&D)** Immunoblot analysis of serine phosphorylation level (C) and ubiquitination level (D) of immunoprecipitated bcIKKε from HEK293T cells transfected as indicated (15 μg/dish). **(E)** HEK293T cells in 10 cm dishes were co-transfected with wild-type or mutant ubiquitin constructs together with either bcIKKε alone or with both bcIKKε and bcTANK (15 μg/dish). bcIKKε ubiquitination levels were assessed by immunoblotting, and relative intensities were quantified by grayscale analysis.

Having established that bcTANK promoted the IFN-inducing and antiviral functions of bcIKKε, we sought to elucidate the underlying regulatory mechanism. Given that the activity of IKKε is associated with its ubiquitination and phosphorylation status, we assessed the impact of bcTANK on these post-translational modifications of bcIKKε using mass spectrometry. The results revealed that co-expression of bcTANK and bcIKKε markedly increased the phosphorylation level of bcIKKε while concomitantly reducing its ubiquitination level (Fig 5A and 5B). These findings were further validated by immunoprecipitation assays, which confirmed that bcTANK enhanced bcIKKε general serine phosphorylation and diminished its ubiquitination (Fig 5C and 5D). To delineate the specific ubiquitination linkages modulated by bcTANK, we employed a panel of ubiquitin mutants in which specific lysine residues were substituted. Co-transfection experiments showed that bcTANK significantly suppressed the wild-type ubiquitin modification of bcIKKε. Moreover, bcTANK markedly inhibited K27-, K33-, K48-, and K63-linked ubiquitinations of bcIKKε, while exerting little impact on K6-, K11-, and K29-linked ubiquitinations (Fig 5E). In summary, these results indicated that bcTANK inhibited multiple forms of polyubiquitination modifications on bcIKKε, including K27-, K33-, K48-, and K63-linked modifications, while enhancing its phosphorylation.

### Lysine residues K418 and K545 of bcIKKε were important for bcTANK-triggered antiviral activity

**Fig 6:**
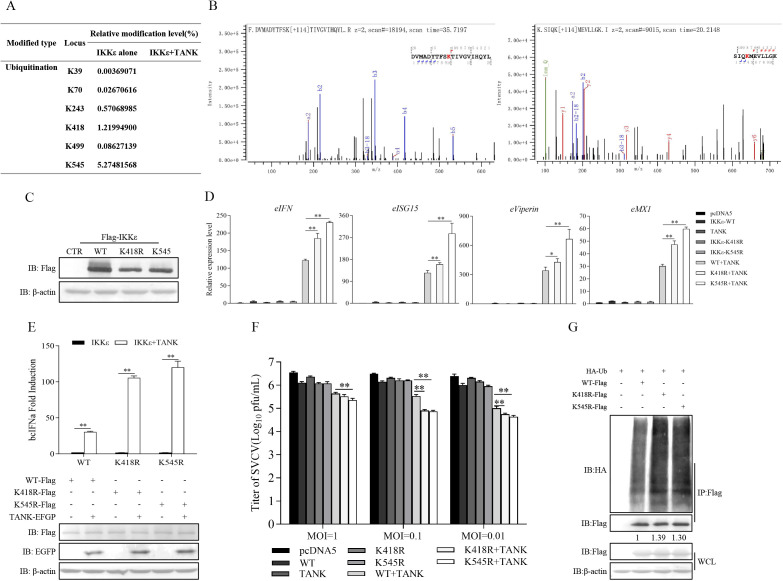
Lysine residues K418 and K545 of bcIKKε are critical for bcTANK-mediated antiviral activation. **(A)** Mass spectrometry identified bcIKKε ubiquitination sites regulated by bcTANK. **(B)** Mass spectra of bcIKKε peptides showing ubiquitination at K418 (sequence: DVMADYTFSKTIVGVIHQYL) and K545 (sequence: SIQKMEVLLGK). Ubiquitinated lysins are highlighted in red. **(C)** Western blot analysis confirmed expression of wild-type and mutant bcIKKε proteins. **(D)** EPC cells in 24-well plates were transfected with bcIKKε (or its mutants), either alone or in combination with bcTANK (500 ng/well), and used for qRT-PCR analysis. **(E)** EPC cells were transfected with the indicated plasmids (525 μg/well) and used for dual-luciferase assay. The protein expression levels of the corresponding plasmids were detected by western blotting assay. **(F)** Viral titers in supernatants from EPC cells in 24-well plates transfected as indicated (500 ng/well), then infected with SVCV at indicated MOIs. Data represented the means ± SEM (n = 3) and were tested for statistical significance using a two-tailed Student’s t-test. **, P < 0.01. **(G)** HEK293T cells were co-transfected with indicated plasmids (15 μg/dish) and used for co-IP analysis at 48 h post-transfection. The relative intensities were quantified by grayscale analysis.

To identify the key ubiquitination sites of bcIKKε induced by bcTANK, we integrated the mass spectrometry data and pinpointed two lysine residues: K418 and K545 (Fig 6A and 6B). Accordingly, two ubiquitin deficient mutants, bcIKKε-K418R and bcIKKε-K545R, were constructed. Structural prediction using ROBETTA revealed that substitution of K418 and K545 with arginine did not significantly alter the secondary or tertiary structure of bcIKKε (S4 Fig). Immunoblotting confirmed that the mutants were successfully expressed in the EPC cells (Fig 6C). To assess the functional impact of these mutations, we performed qRT-PCR analysis to examine the mRNA levels of key antiviral genes (*eIFN*, *eISG15*, *eViperin*, and *eMX1*) in EPC cells overexpressing bcIKKε or its mutants, either alone or together with bcTANK. As shown in Fig 6D, overexpression of bcIKKε or the mutants alone did not significantly alter the mRNA levels of above antiviral genes. However, when co-transfected with bcTANK, the K418R and K545R mutants induced significantly higher mRNA levels of antiviral genes compared to the wild-type bcIKKε co-expression group. In addition, we carried out the dual-luciferase reporter assays. The induced bcIFNa promoter activation by K418R and K545R mutants was similar to that of wild-type bcIKKε. However, upon co-expression with bcTANK, the mutants triggered significantly stronger induction of the bcIFNa promoter (Fig 6E). Consistently, viral titration assays following SVCV infection showed that viral titers in the supernatants of EPC cells co-expressing K418R or K545R with bcTANK were significantly lower than those of the cells co-expressing bcIKKε with bcTANK (Fig 6F). Furthermore, co-IP assays showed that the ubiquitination levels of K418R and K545R mutants were higher than that of wild-type bcIKKε (Fig 6G). Taken together, these results demonstrated that lysine residues K418 and K545 were critical sites through which bcTANK enhanced bcIKKε-mediated induction of antiviral responses.

### bcTANK recruited bcUSP46 to deubiquitinate bcIKKε

**Fig 7:**
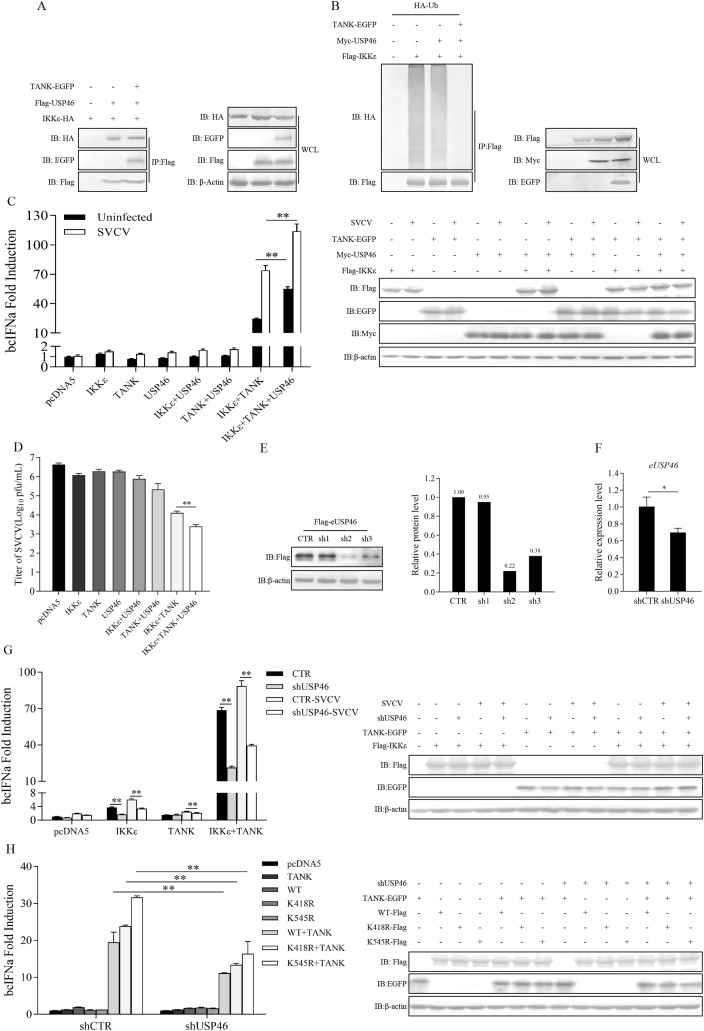
bcTANK recruits bcUSP46 to deubiquitinate bcIKKε. **(A&B)** HEK293T cells in 10 cm dishes were co-transfected with indicated plasmids (15 μg/dish) for 48 **h.** Whole-cell lysates were collected for co-IP to analyze protein interactions (A) and ubiquitination levels of bcIKKε **(B)**. **(C)** Dual-luciferase reporter assay of bcIFNa promoter activity in EPC cells transfected with the indicated plasmids (525 ng/well). The protein expression levels of the corresponding plasmids were detected by western blotting assay. **(D)** Viral titers in supernatants from EPC cells transfected as indicated and infected with SVCV (MOI = 0.1) for 24 **h. (E)** Detection of knockdown efficiency of shRNA targeting EPC-USP46. **(F)** Validation of USP46 knockdown in stable EPC cell lines. (G) bcIFNa promoter activity in control and USP46-knockdown EPC cells transfected with bcIKKε, bcTANK, and reporter plasmids (525 ng/well), followed by SVCV infection (MOI = 0.1). The protein expression levels of the corresponding plasmids were detected by western blotting assay. **(H)** In USP46-knockdown EPC cells and control cells, wild-type and mutant bcIKKε and bcTANK were overexpressed as indicated (525 ng/well). The bcIFNa promoter activity was detected using a dual-luciferase reporter assay. The protein expression levels of the corresponding plasmids were detected by western blotting assay. Data represented the means ± SEM and were tested for statistical significance using a two-tailed Student’s t-test. **, P < 0.01.

Since bcTANK lacked intrinsic deubiquitinating activity, we hypothesized that it recruited a deubiquitinating enzyme (DUB) to suppress bcIKKε ubiquitination. Mass spectrometry analysis of bcIKKε-associated proteins identified bcUSP46 as a potential interacting DUB. Co-IP assays further confirmed the interaction between bcIKKε and bcUSP46, which was further strengthened in the presence of bcTANK (Fig 7A). To investigate whether bcUSP46 mediated the deubiquitination of bcIKKε by bcTANK, we performed co-IP assay. The result showed that overexpression of bcUSP46 further enhanced the deubiquitinating effect of bcTANK on bcIKKε (Fig 7B). Subsequent dual-luciferase reporter assays and viral titration experiments demonstrated that bcUSP46 cooperated with bcTANK to potentiate bcIKKε-driven bcIFNa promoter activation and antiviral response (Fig 7C and 7D). To further define the role of endogenous eUSP46, we generated shRNAs (sh-1,2,3) targeting eUSP46. Knockdown efficiency revealed that sh-2 and sh-3 effectively suppressed eUSP46 expression (Fig 7E). EPC cells stably expressing sheUSP46–2 were established (Fig 7F) and used for dual-luciferase reporter assays under SVCV-infected or uninfected conditions. These results showed that knockdown of eUSP46 significantly reduced bcIKKε-mediated bcIFNa promoter transcription and markedly impaired bcTANK-enhanced bcIKKε activation (Fig 7G). This impairment was also observed in the ubiquitination-deficient mutants (K418R and K545R) of bcIKKε (Fig 7H). Collectively, these findings indicated that bcUSP46 was specifically recruited by bcTANK to deubiquitinate bcIKKε, thereby promoting bcIKKε-mediated IFN production and antiviral activity.

### SVCV P inhibited bcIKKε-mediated antiviral immunity through its PCD domain

**Fig 8:**
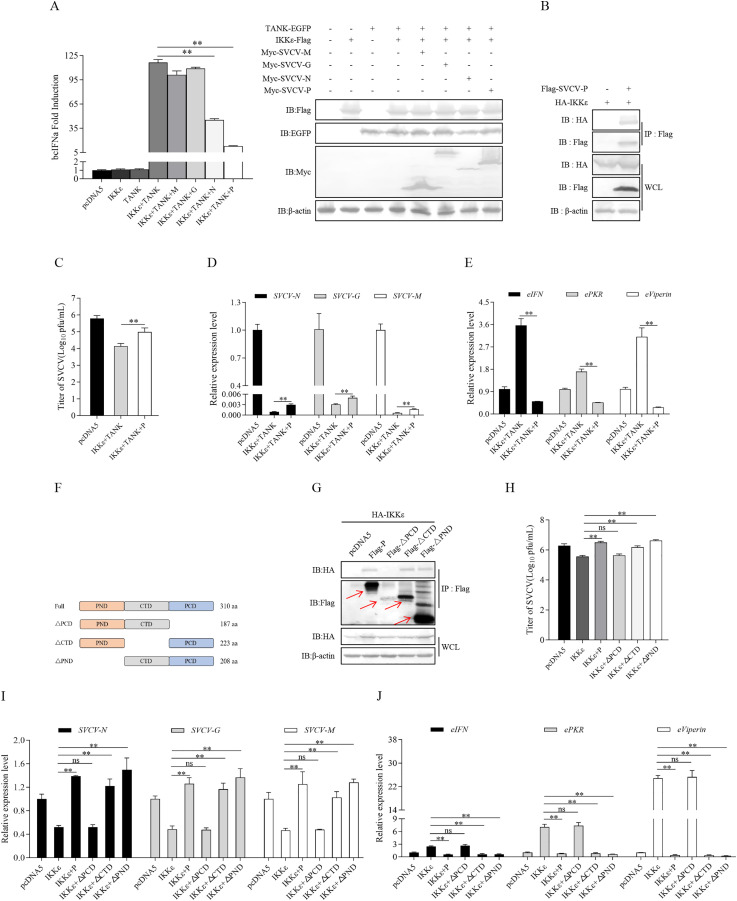
The PCD domain of SVCV P is essential for inhibiting bcIKKε-mediated antiviral signaling. **(A)** EPC cells seeded in 24-well plates were co-transfected with bcIKKε, bcTANK, and individual SVCV-encoded proteins (525 ng/well). At 24 h post-transfection, cells were harvested, and bcIFNa promoter activity was measured using a dual-luciferase reporter assay. The expression levels of the corresponding proteins were detected by western blotting. **(B)** Co-IP assays were performed to confirm the interaction between SVCV P and bcIKKε. **(C)** EPC cells were co-transfected with both bcTANK and bcIKKε, either in the absence or presence of SVCV **P.** At 24 h post-transfection, cells were infected with SVCV (MOI = 0.1). At 24 h post-infection, culture supernatants were collected, and viral titers were determined by plaque assay. (D&E) qRT-PCR analysis of SVCV-encoded gene (D) and antiviral factors (*eIFN*, *ePKR*, *eViperin*) (E) expression in cells from **(C)**. **(F)** Mapping of the SVCV P protein domain. **(G)** Full-length and truncated P proteins were co-expressed with bcIKKε and subjected to co-IP. **(H-J)** EPC cells were co-transfected with the indicated plasmids and subsequently infected with SVCV (MOI = 0.1). Viral replication was assessed by viral titration (H) and mRNA levels of of viral genes (I) and antiviral factors (J) were examined by qRT-PCR analysis.

To identify viral antagonists of the bcTANK-bcIKKε signaling axis, we screened SVCV-encoded proteins by dual-luciferase reporter assays. The results showed that the viral P and N proteins significantly suppressed bcTANK/bcIKKε-induced bcIFNa promoter activation, with the SVCV P protein exhibiting the strongest inhibitory effect (Fig 8A). Co-IP assays confirmed that SVCV P protein interacted with bcIKKε (Fig 8B). Virus titer assays and qRT-PCR results demonstrated that co-expression of SVCV P protein with bcIKKε and bcTANK significantly elevated viral titers and increased mRNA levels of SVCV-encoded genes compared to bcIKKε-bcTANK co-expression group, accompanied by a marked reduction in the expression of antiviral factors, including *eIFN, ePKR* and *eViperin* (Fig 8C-8E).

To further identify the domain of SVCV P protein responsible for bcKKε inhibition, we generated a series of SVCV P truncated mutants (Fig 8F). Co-IP analysis showed that deletion of the PCD domain abolished the major interaction between SVCV P and bcKKε, indicating that the PCD domain was required for bcKKε binding (Fig 8G). Subsequent viral titer assays showed that, unlike full-length SVCV P, the ΔPCD mutant failed to efficiently counteract the antiviral effect of bcKKε (Fig 8H). Accordingly, qRT-PCR analysis demonstrated that ΔPCD failed to enhance viral gene expression and failed to suppress antiviral gene expression compared with full-length SVCV P (Fig 8I and 8J). Collectively, these findings demonstrated that the PCD domain of SVCV P was essential for its interaction with bcIKKε and for its inhibition of bcIKKε-mediated antiviral signaling.

### SVCV P disrupted bcIKKε-associated functional complexes and altered its post-translational modification

**Fig 9:**
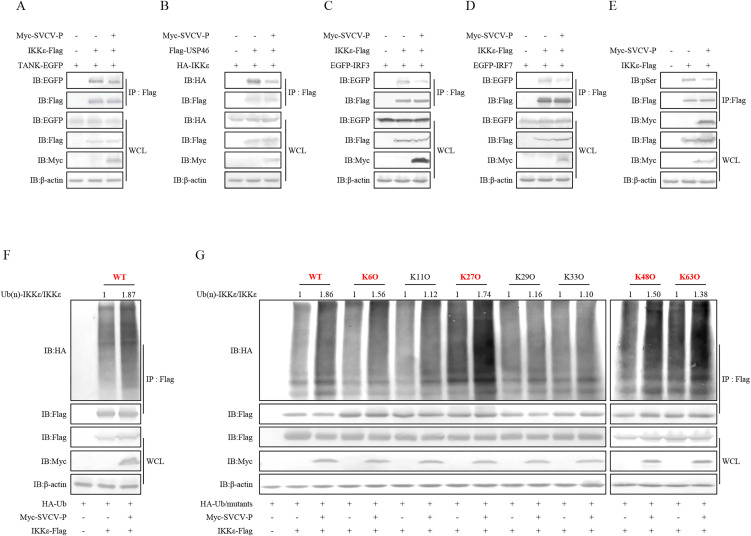
SVCV P protein disrupts bcIKKε-associated functional complexes and alters bcIKKε post-translational modifications. **(A-D)** HEK293T cells were co-transfected with indicated plasmids and collected for co-IP analysis at 48 h post-transfection. The effects of SVCV P on the interactions between bcIKKε and bcTANK **(A)**, bcUSP46 **(B)**, bcIRF3 **(C)**, or bcIRF7 (D) were examined. **(E)** The general serine phosphorylation level of bcIKKε was analyzed in the presence or absence of SVCV **P. (F, G)** HEK293T cells were co-transfected with the indicated plasmids and harvested for co-IP analysis at 48 h post-transfection. The overall ubiquitination level of bcIKKε (F) and the specific ubiquitin linkage types on bcIKKε (G) were analyzed.

To elucidate the mechanism by which SVCV P inhibited bcIKKε-mediated antiviral signaling, we examined whether SVCV P affected the formation of bcIKKε-associated signaling complexes. Co-IP assays in HEK293T cells (Fig 9A and 9B) and EPC cells (S5 Fig) showed that SVCV P impaired the interaction between bcIKKε and bcTANK, as well as the interaction between bcIKKε and bcUSP46. Moreover, SVCV P also weakened the association of bcIKKε with the downstream transcription factors bcIRF3 and bcIRF7 (Fig 9C and 9D). These results suggested that SVCV P disrupted both upstream regulatory complex formation and downstream effector recruitment in the bcIKKε signaling cascade.

We next examined whether SVCV P influenced phosphorylation and ubiquitination of bcIKKε. Co-IP analysis showed that SVCV P reduced the general serine phosphorylation level of bcIKKε (Fig 9E). In contrast, SVCV P markedly enhanced the overall ubiquitination level of bcIKKε (Fig 9F). Further ubiquitination assays using ubiquitin mutants revealed that SVCV P promoted K6-, K27-, K48-, and K63-linked polyubiquitination of bcIKKε, whereas it had little effect on K11-, K29-, and K33-linked polyubiquitination (Fig 9G). Collectively, these findings demonstrated that SVCV P interacted with bcIKKε, suppressed its phosphorylation, enhanced multiple types of bcIKKε polyubiquitination, and disrupted the association between bcIKKε with bcTANK, bcUSP46, bcIRF3, or bcIRF7, which jointly contributed to the inhibition of the bcIKKε-mediated antiviral signaling pathway.

## Discussion

TANK functions as a scaffold protein that orchestrates interactions within the IKK signaling complex [41]. It can form trimers with TBK1 and TRAF2, facilitating TBK1 activation, and can aggregate with both upstream adaptors (MAVS, TRIF, TRAF3) and downstream effectors (TBK1, IKKε, IRF3) [23]. Such multivalent interactions likely enhance the efficiency and specificity of IFN induction. IKKε, a key non-canonical IKK family member, has been reported to mediate phosphorylation and ubiquitination of TANK [7,42]. However, whether TANK reciprocally regulates PTMs of IKKε is remained unclear. Our finding suggest that black carp TANK can modulate IKKε by reducing its ubiquitination and promoting its phosphorylation, providing a novel mechanistic insight: TANK not only organizes signaling complexes but also fine-tunes IKKε activity through direct PTMs (Fig 5-6). This regulatory axis may potentiate IFN signaling and antiviral immune responses, highlighting a previously unrecognized layer of control within the non-canonical IKK pathway. These insights advance our understanding of IKKε regulation and may inform the design of targeted immunomodulatory strategies in teleost fish.

IKKε is a member of the I-κB kinase family, and its function is closely linked to its kinase activity [10,11,14]. However, IKKε requires homodimerization and possibly higher-order complex formation, such as homotetramers or homooctamers, to fully activate its kinase function. This suggests that its activity is modulated by the extent of its aggregation [43]. Our immunoprecipitation experiments confirmed that, similar to mammalian IKKε, bcIKKε undergoes self-aggregation. Additionally, bcTANK was found to significantly enhance bcIKKε aggregation (Fig 4C). As downstream molecules in the RLR signaling pathway, bcIRF3 and bcIRF7, consistent with their mammalian counterparts, primarily localize to the cytoplasm in the resting state. Upon activation by upstream signals, they translocate from the cytoplasm into the nucleus [17–19,44]. Our data indicate that bcTANK enhances the nuclear translocation of bcIRF3 and bcIRF7 through bcIKKε activation (Fig 4D and 4E).

The PTMs of IKKε, particularly ubiquitination, is critical for regulating its activity [14,20]. Protein ubiquitination, one of the most extensively studied PTMs, includes canonical ubiquitination, such as K48- and K63-linked chains, and non-canonical ubiquitination, such as K6-, K11-, K27-, K29-, and K33-linked chains [45]. Canonical K48-linked ubiquitination typically targets substrate proteins for proteasomal degradation, whereas K63-linked ubiquitination is often associated with the stabilization and activation of signaling protein [45]. Our data demonstrated that bcTANK reduced K48-linked ubiquitination of bcIKKε, which may prevent its degradation and enhance protein stability (Fig 5E). Non-canonical K27- and K33-linked ubiquitination also contributes to the regulation of innate immunity [45]. For instance, K27-linked ubiquitination of stimulator of IFN genes (STING) provides a platform for TBK1 recruitment and facilitates its translocation to perinuclear microsomes, whereas removal of this chain from STING suppresses TBK1-mediated IFN activation [46–48]. Similarly, removal of K33-linked chains from TBK1 by USP38 allows subsequent K48-linked ubiquitination and proteasomal degradation [49]. In the present study, TANK reduced K27-, K33-. K48- and K63-linked ubiquitination on another non-canonical IKK kinase, IKKε, and this reduction was associated with enhanced IKKε activity (Fig 5E). Mechanistically, this finding suggests a complex regulatory model. Reduced K48-linked ubiquitination may stabilize IKKε by limiting proteasomal degradation, whereas the decrease in K63-linked ubiquitination, which is conventionally associated with signaling activation, appear paradoxical. One possible explanation is that the functional outcome of K63-linked ubiquitination depends on its position, timing, chain architecture, or interaction with other ubiquitin linkage types. Alternatively, coordinated reduction of multiple ubiquitin linkages may alter IKKε conformation, facilitate its phosphorylation, or promote assembly of an active signaling complex. Further studies are required to determine how these distinct ubiquitin linkages cooperate to regulate IKKε activity. Collectively, these findings reveal a nuanced regulatory mechanism in which TANK fine-tunes IKKε activity through coordinated modulation of multiple ubiquitin linkages.

IKKε activation is closely associated with innate immune regulation and tumorigenesis, and several ubiquitination sites within IKKε have been identified. For example, K63-linked ubiquitination at lysine residues K30 and K401 is essential for IKKε kinase activity, and the K30R/K401R mutant completely abolishes IKKε ubiquitination and autophosphorylation [50]. In addition, lysine 416 (K416) of IKKε is also ubiquitinated, although its functional role appears to be partially redundant [50]. Interestingly, factor inhibiting hypoxia-inducible factor (FIH) attenuates ubiquitination of IKKε at K416 and negatively regulates antiviral immune response [51]. In the present study, we identified K418 and K545 as critical ubiquitination sites of black carp IKKε (Fig 6). Among these residues, black carp K418 is conserved with human K416, whereas black carp K545 lacks a corresponding residue in human IKKε. Our finding demonstrated that TANK-mediated removal of ubiquitination at K418 and K545 positively regulated IKKε-mediated antiviral immunity. Collectively, these findings expand the current understanding of IKKε post-translational regulation and highlight the evolutionary diversity of antiviral signaling mechanisms in vertebrates.

Extensive research has demonstrated that many viruses rely on encoded structural or non-structural proteins to bind with specific host proteins, thereby enhancing their entry efficiency or suppressing host immune response to achieve immune evasion [24,26,27,52]. SVCV possesses a simple structure, encoding only five structural proteins: M, N, G, P, and L [31]. However, several studies have shown that these proteins can suppress host antiviral capabilities through certain mechanisms [32,33,53]. Our data show that, SVCV P protein can interact with bcIKKε (Fig 8B). It can inhibit bcIKKε-mediated bcIFNa promoter activity and antiviral capacity activated by bcTANK (Fig 8A-8E). Further investigations revealed that SVCV P enhances the ubiquitination of bcIKKε, represses its serine phosphorylation, and disrupts its interactions with bcTANK, bcUSP46, and bcIRF3/7, ultimately suppressing bcIKKε-mediated antiviral activity (Fig 9).

Teleost, particularly cyprinids, are highly susceptible to viral pathogens such as SVCV and GCRV in aquaculture systems [29,31,54–58]. Previous studies have shown that viruses employ multiple immune evasion strategies to promote their replication. For example, SVCV modulates p53 protein level to suppress immune surveillance [59]. During the early stage of infection, the SVCV N protein decreases p53 expression by inhibiting K63-linked ubiquitination of K358, whereas during the late stage, the SVCV P protein increases p53 expression by enhancing K63-linked ubiquitination at the same residue. In addition, previous reports have demonstrated that the SVCV P protein targets the TBK1 axis by acting as a decoy substrate for TBK1, thereby reducing TBK1-mediated IRF3 phosphorylation and suppressing IFN signaling [33]. Our finding extends these previous observations by showing that the SVCV P protein also targets another non-canonical IKK family kinase, IKKε (Fig 8 and 9). This suggests that SVCV P does not merely interfere with a single kinase branch of the antiviral pathway but may broadly antagonize the bcTBK1/bcIKKε-bcIRF3/7 signaling module. Mechanistically, SVCV P blocks the association of bcIKKε with bcTANK, bcUSP46, bcIRF3, and bcIRF7 (Fig 9A-9D). Moreover, SVCV P protein suppresses bcIKKε phosphorylation, whereas enhances K6-, K27, K48-, and K63-linked ubiquitination of bcIKKε (Fig 9E-9G). bcTANK-mediated reduction of several ubiquitin linkage types was associated with bcIKKε activation in our study, SVCV P protein-induced increase in these ubiquitin chains may represent an opposing viral strategy to restrain bcIKKε function. Together with prior reports on the TBK1 axis, our results suggest that SVCV P functions as a multifunctional antagonist of non-canonical IKK signaling, targeting both TBK1- and IKKε-dependent antiviral pathways to evade innate immune responses.

**Fig 10:**
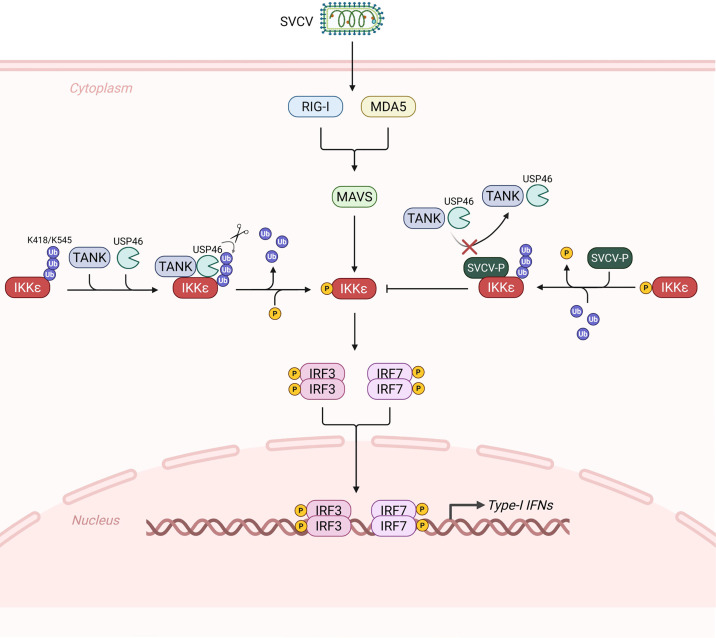
The model of bcTANK-bcUSP46-bcIKKε signaling axis and its antagonism by SVCV P protein. Upon SVCV infection, viral RNA is recognized by the cytosolic pattern recognition receptors bcRIG-I and bcMDA5, which activate bcMAVS signaling. Activated bcMAVS recruits bcIKKε, leading to the phosphorylation and activation of downstream transcription factors bcIRF3 and bcIRF7, which translocate into the nucleus to induce IFN expression. The adaptor protein bcTANK promotes bcIKKε activation by enhancing its phosphorylation and reducing its ubiquitination at key lysine residues (K418 and K545). This deubiquitination process is facilitated through the recruitment of the deubiquitinating enzyme bcUSP46 by bcTANK. In contrast, the SVCV-encoded P protein acts as a viral antagonist by binding to bcIKKε, inhibiting its phosphorylation, enhancing its ubiquitination, and disrupting its interactions with bcTANK, bcUSP46, and downstream effectors bcIRF3/bcIRF7. Consequently, bcIKKε-mediated IFN signaling is suppressed, facilitating viral immune evasion. Created in BioRender. Xiao, **J.** (2026) https://BioRender.com/rokqqht.

In summary, this study delineates a TANK-USP46-IKKε signaling axis that potentiates antiviral immunity in black carp, and identifies the SVCV P protein as a viral antagonist that disrupts this axis by promoting IKKε ubiquitination and inhibiting IKKε phosphorylation (Fig 10). Our findings not only advance the molecular understanding of teleost innate immunity but also reveal a novel host-virus interface centered on the ubiquitination control of IKKε. These insights may inform future strategies for the control of viral diseases in aquaculture.

## Supporting information

S1 Fig Viral titer detection in shTANK knockdown fish tissues. Juvenile black carp were intramuscularly injected with either shbcTANK-3 or control scramble shRNA at a dosage of 1 μg plasmid per gram of body weight. Three days post-injection, the fish were challenged with either PBS or SVCV at a concentration of 2 × 10⁶ copies/mL. At 3 days post-infection, gill tissue was harvested and processed for viral titer determination.
(TIF)

S2 Fig bcTANK does not target bcTBK1 to activate IFN-mediated antiviral signaling. EPC cells were co-transfected with bcTBK1 and/or bcTANK as indicated. At 24 h post-transfection, cells were infected with SVCV at an MOI of 0.1, and culture supernatants were collected for viral titer measurement. Data are presented as mean ± SEM (n = 3), and statistical significance was evaluated using a two-tailed Student’s t-test.
(TIF)

S3 Fig bcTANK interacts with bcIKKε in EPC cells. (A-B) EPC cells were co-transfected with bcIKKε-Flag and/or bcTANK-EGFP at 15 μg per dish. At 48 h post-transfection, EPC cells were harvested and subjected to co-IP analysis to examine the physical interaction between bcTANK and bcIKKε.
(TIF)

S4 Fig Structure comparison of bcIKKε and its mutants. The three-dimensional structures of bcIKKε and its mutant (K418R/K545R) were predicted by ROBETTA. Structural alignment and comparison were performed to evaluate potential conformational changes induced by specific mutations.
(TIF)

S5 Fig SVCV P blocks interaction between bcIKKε and bcTANK/bcUSP46. (A**-**B) EPC cells in 10 cm dishes were co-transfected with the indicated plasmids (15 μg/dish). At 48 h post-transfection, cells were harvested for co-IP analysis to assess the impact of SVCV P on the interactions between bcIKKε and its regulatory partners, bcTANK or bcUSP46.
(TIF)

S1 Data Raw data. Excel file containing raw data underlying all figures in this study.
(XLS)

S1 File Raw image. PDF file containing raw WB underlying all figures in this study.
(PDF)
