# Supplementary figures and images for: TANK potentiates antiviral innate immunity by recruiting deubiquitinase USP46 to activate IKKε
Source: PLoS Pathog. 2026 Jul 10;22(7):e1014412. doi: 10.1371/journal.ppat.1014412 (PMC13353983; doi:10.1371/journal.ppat.1014412)

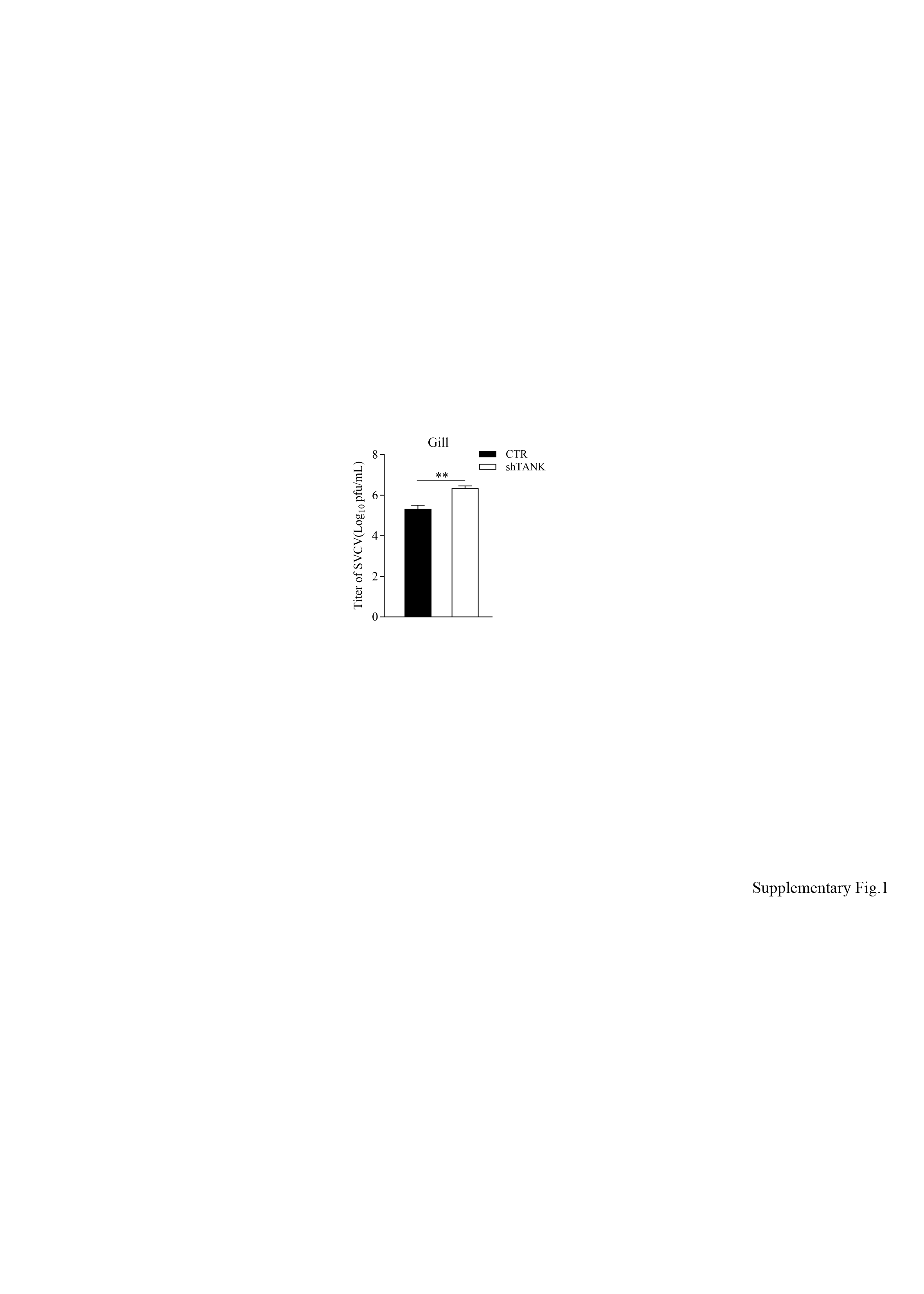

Supplement: S1 Fig — Juvenile black carp were intramuscularly injected with either shbcTANK-3 or control scramble shRNA at a dosage of 1 μg plasmid per gram of body weight. Three days post-injection, the fish were challenged with either PBS or SVCV at a concentration of 2 × 10⁶ copies/mL. At 3 days post-infection, gill tissue was harvested and processed for viral titer determination. (TIF) [file ppat.1014412.s001.tif]

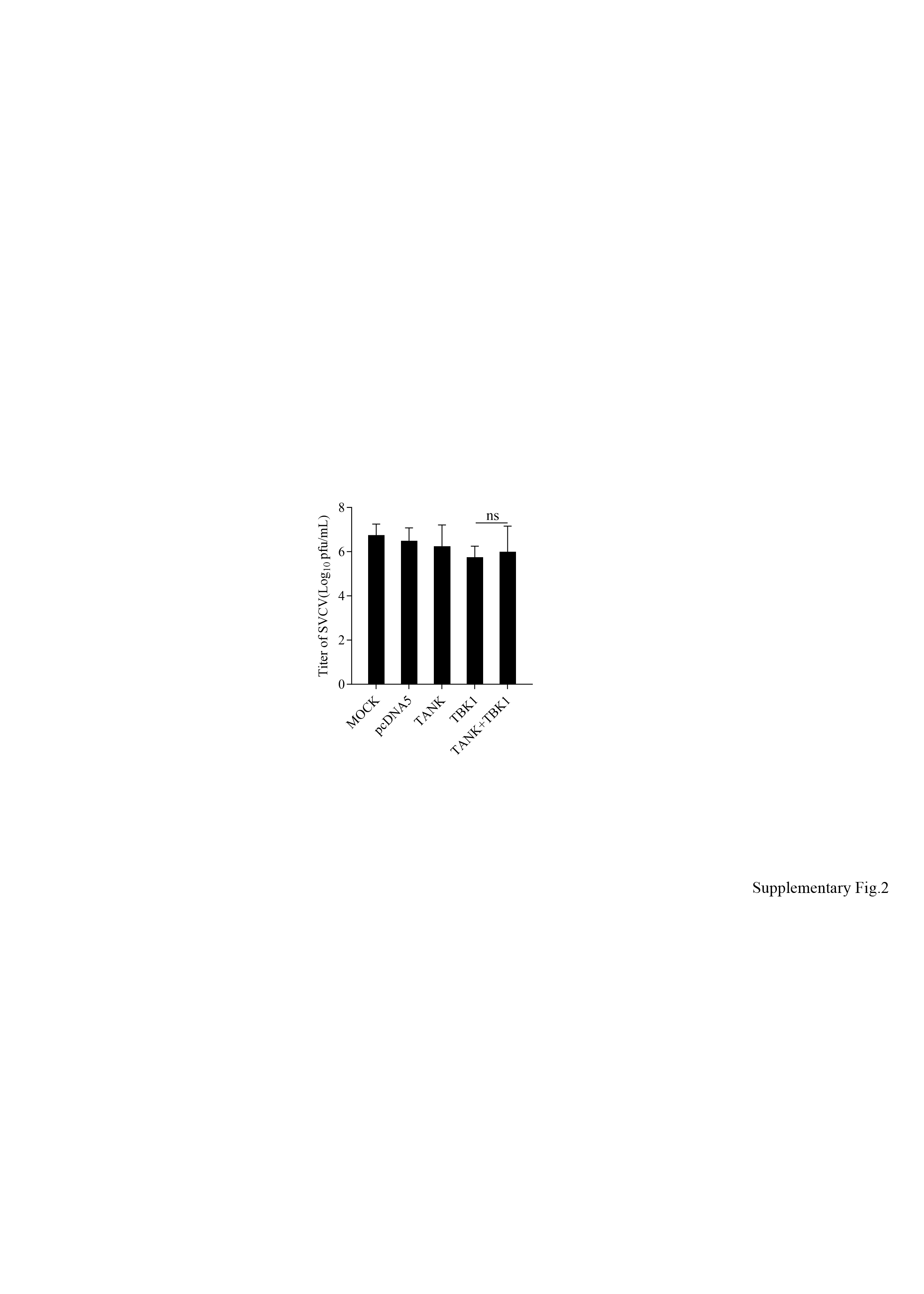

Supplement: S2 Fig — EPC cells were co-transfected with bcTBK1 and/or bcTANK as indicated. At 24 h post-transfection, cells were infected with SVCV at an MOI of 0.1, and culture supernatants were collected for viral titer measurement. Data are presented as mean ± SEM (n = 3), and statistical significance was evaluated using a two-tailed Student’s t-test. (TIF) [file ppat.1014412.s002.tif]

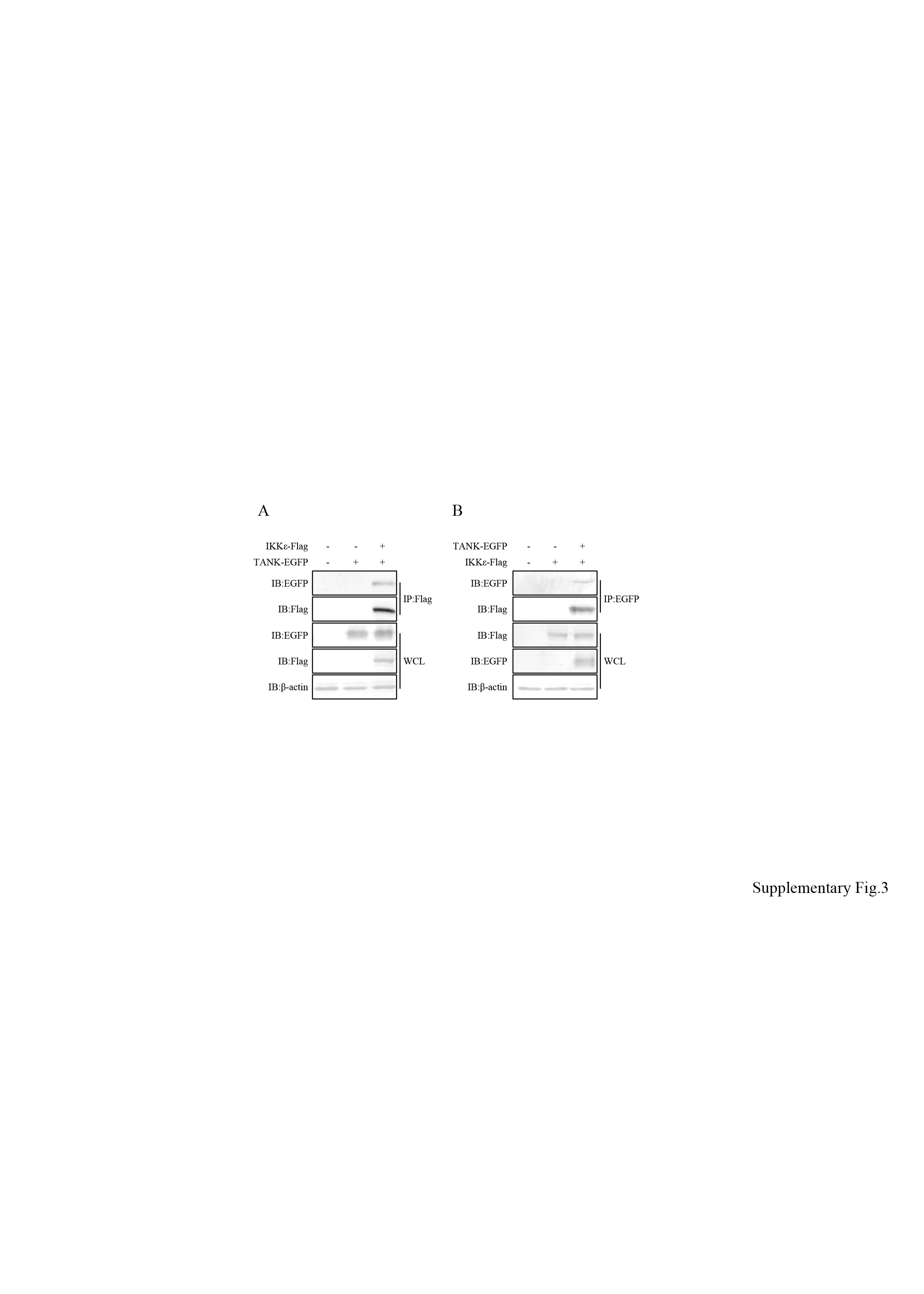

Supplement: S3 Fig — (A-B) EPC cells were co-transfected with bcIKKε-Flag and/or bcTANK-EGFP at 15 μg per dish. At 48 h post-transfection, EPC cells were harvested and subjected to co-IP analysis to examine the physical interaction between bcTANK and bcIKKε. (TIF) [file ppat.1014412.s003.tif]

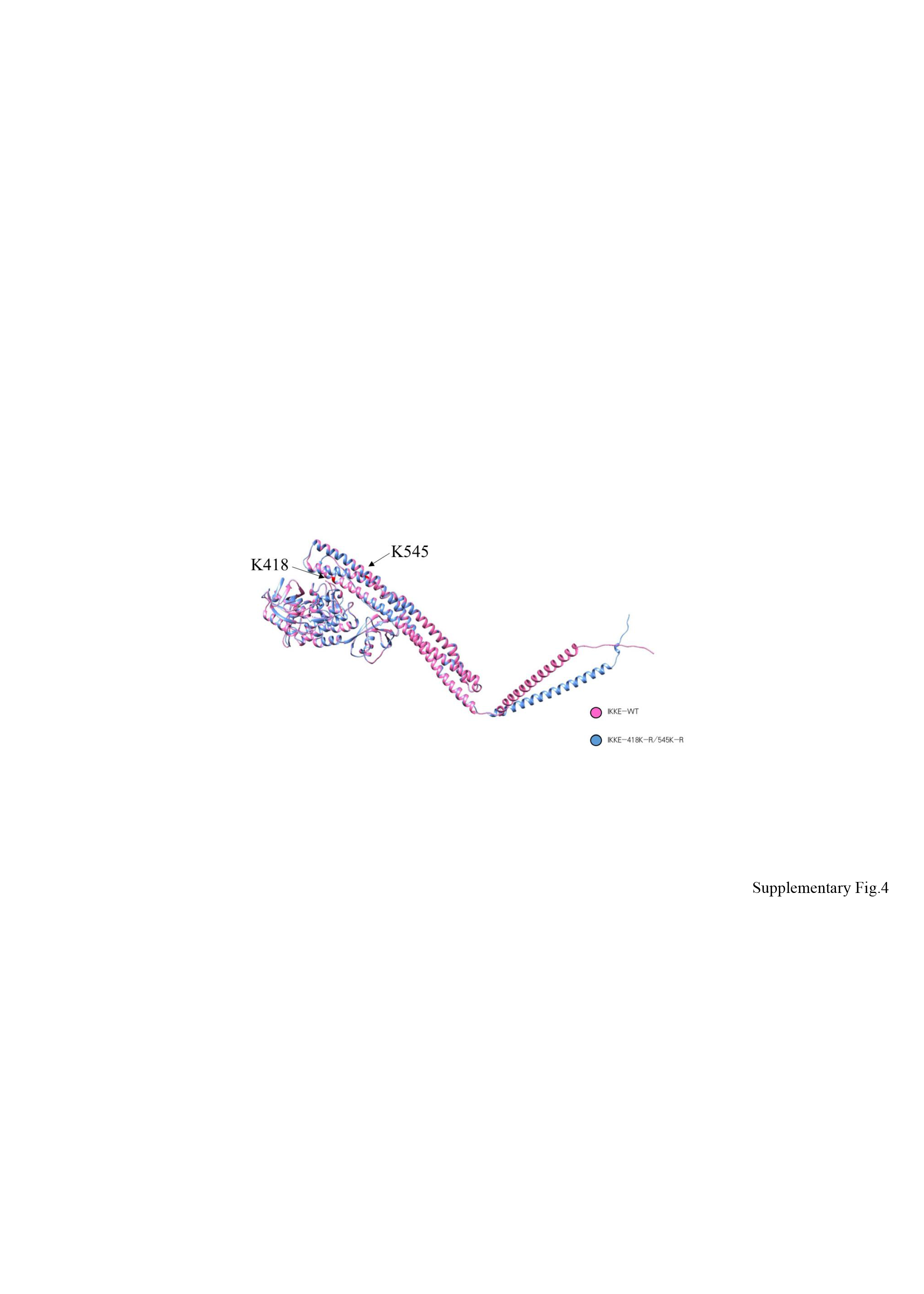

Supplement: S4 Fig — The three-dimensional structures of bcIKKε and its mutant (K418R/K545R) were predicted by ROBETTA. Structural alignment and comparison were performed to evaluate potential conformational changes induced by specific mutations. (TIF) [file ppat.1014412.s004.tif]

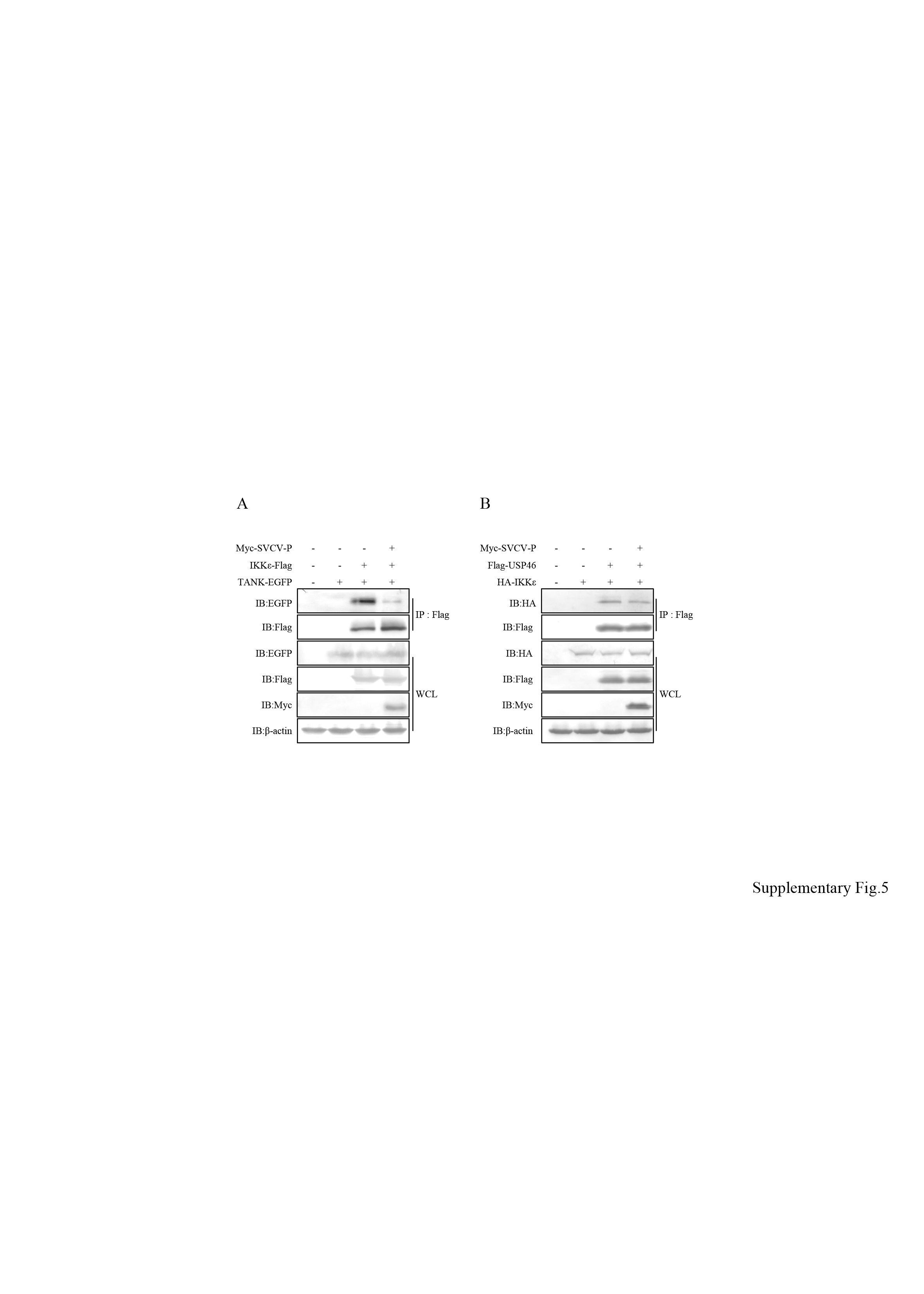

Supplement: S5 Fig — (A-B) EPC cells in 10 cm dishes were co-transfected with the indicated plasmids (15 μg/dish). At 48 h post-transfection, cells were harvested for co-IP analysis to assess the impact of SVCV P on the interactions between bcIKKε and its regulatory partners, bcTANK or bcUSP46. (TIF) [file ppat.1014412.s005.tif]
