# Supplementary material for: TANK potentiates antiviral innate immunity by recruiting deubiquitinase USP46 to activate IKKε
Source: PLoS Pathog. 2026 Jul 10;22(7):e1014412. doi: 10.1371/journal.ppat.1014412 (PMC13353983; doi:10.1371/journal.ppat.1014412)
Supplement: S1 File — PDF file containing raw WB underlying all figures in this study. (PDF) [file ppat.1014412.s007.pdf]

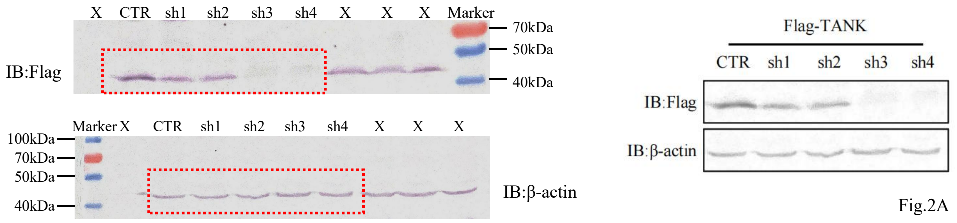
X CTR sh1 sh2 sh3 sh4 X X X Marker
70kDa
50kDa
40kDa
IB:Flag
Marker X CTR sh1 sh2 sh3 sh4 X X X
100kDa
70kDa
50kDa
40kDa
IB:β-actin
Flag-TANK
CTR sh1 sh2 sh3 sh4
IB:Flag
IB:β-actin

Fig.2A

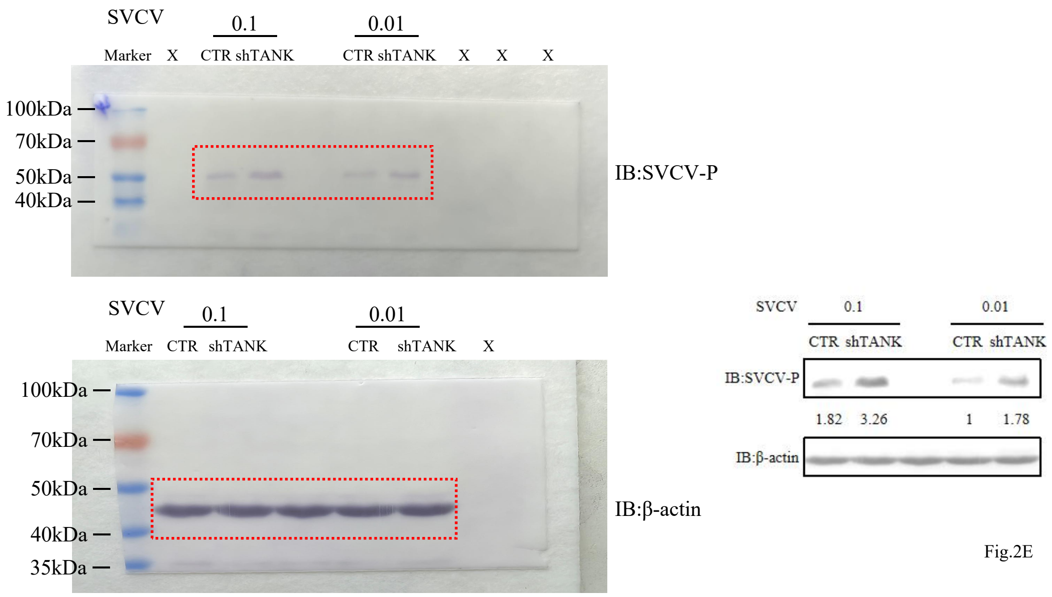
SVCV 0.1 0.01
Marker X CTR shTANK CTR shTANK X X X
100kDa
70kDa
50kDa
40kDa
IB:SVCV-P
SVCV 0.1 0.01
Marker CTR shTANK CTR shTANK X
100kDa
70kDa
50kDa
40kDa
35kDa
IB:β-actin
SVCV 0.1 0.01
CTR shTANK CTR shTANK
IB:SVCV-P
1.82 3.26 1 1.78
IB:β-actin

Fig.2E

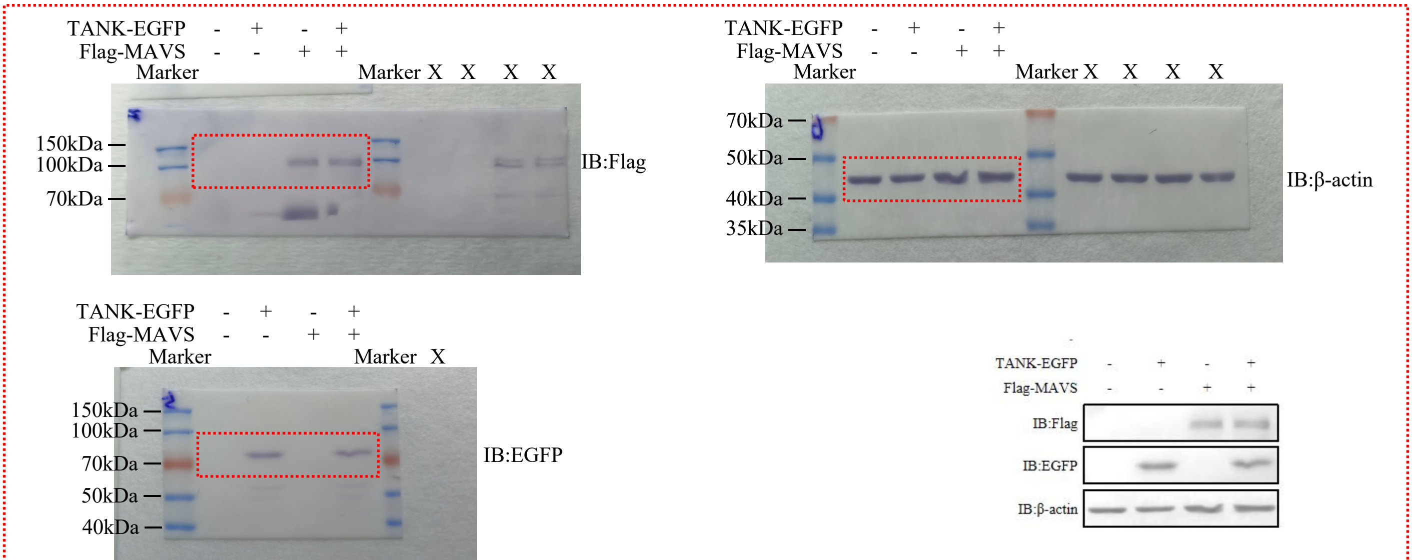

Fig.3A

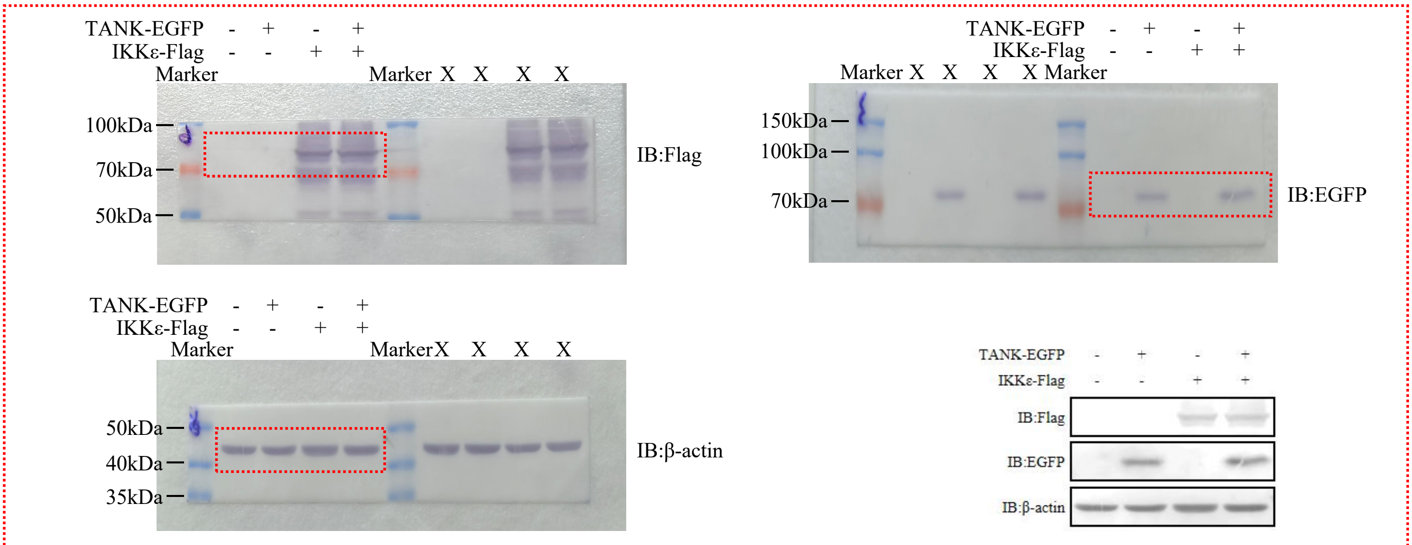

Fig.3A

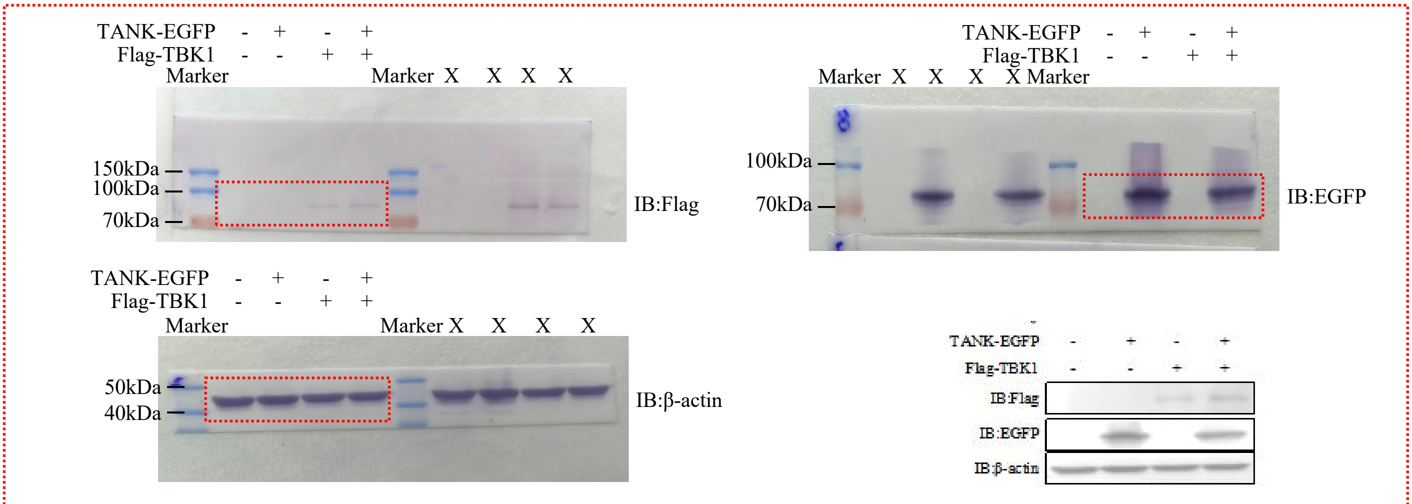

Fig.3A

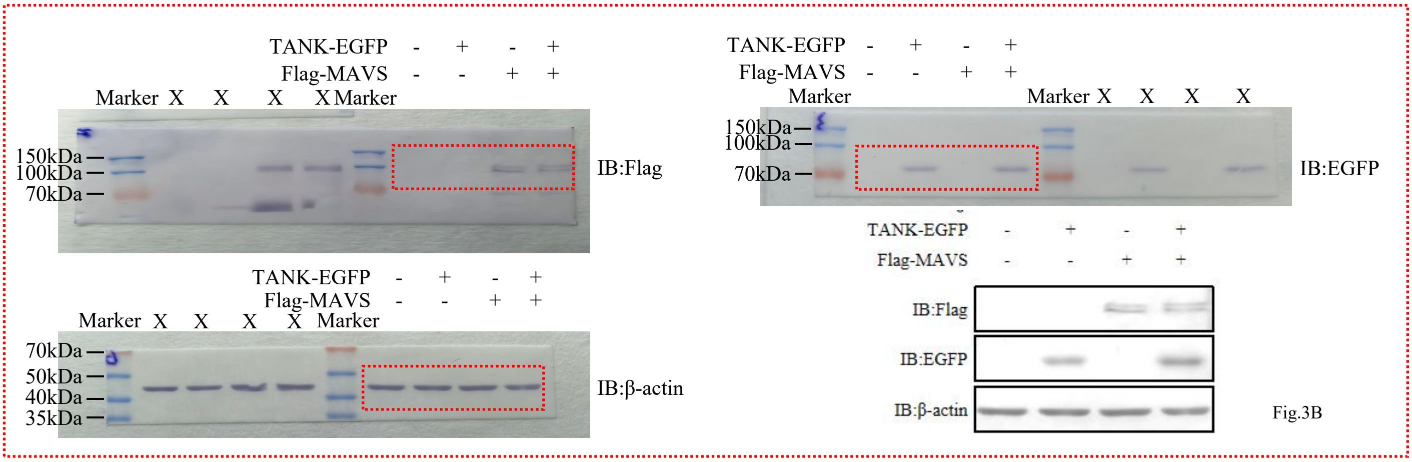

Fig.3B

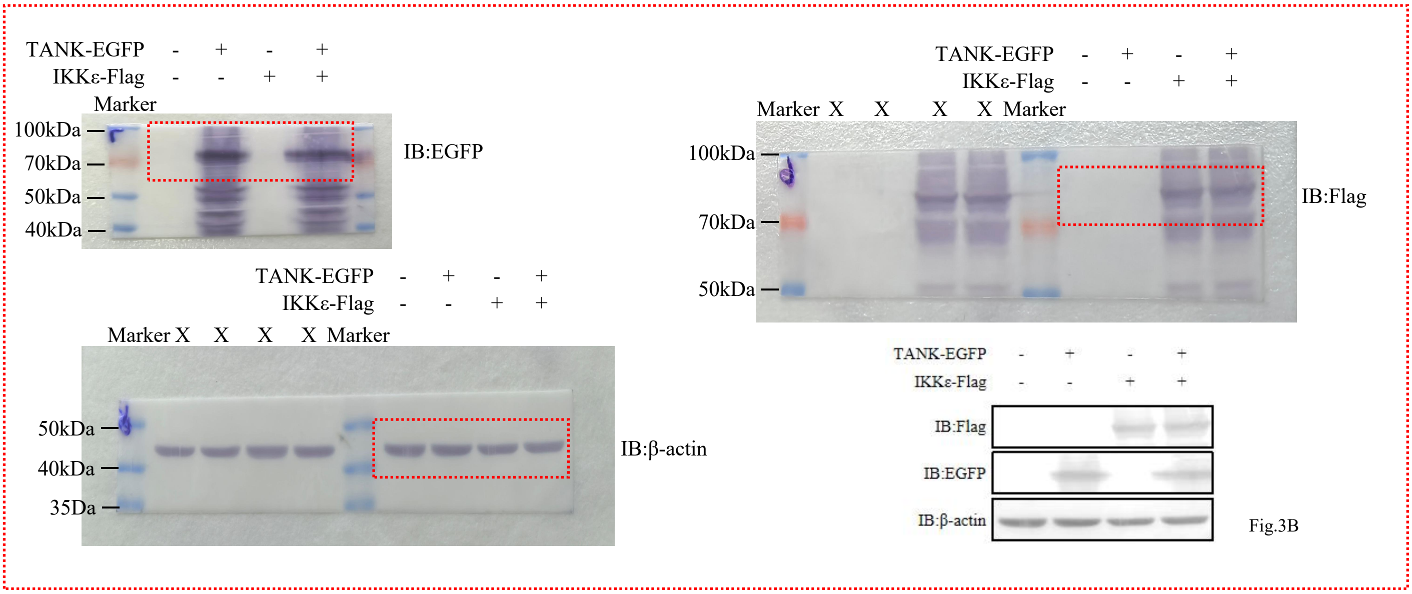

Fig.3B

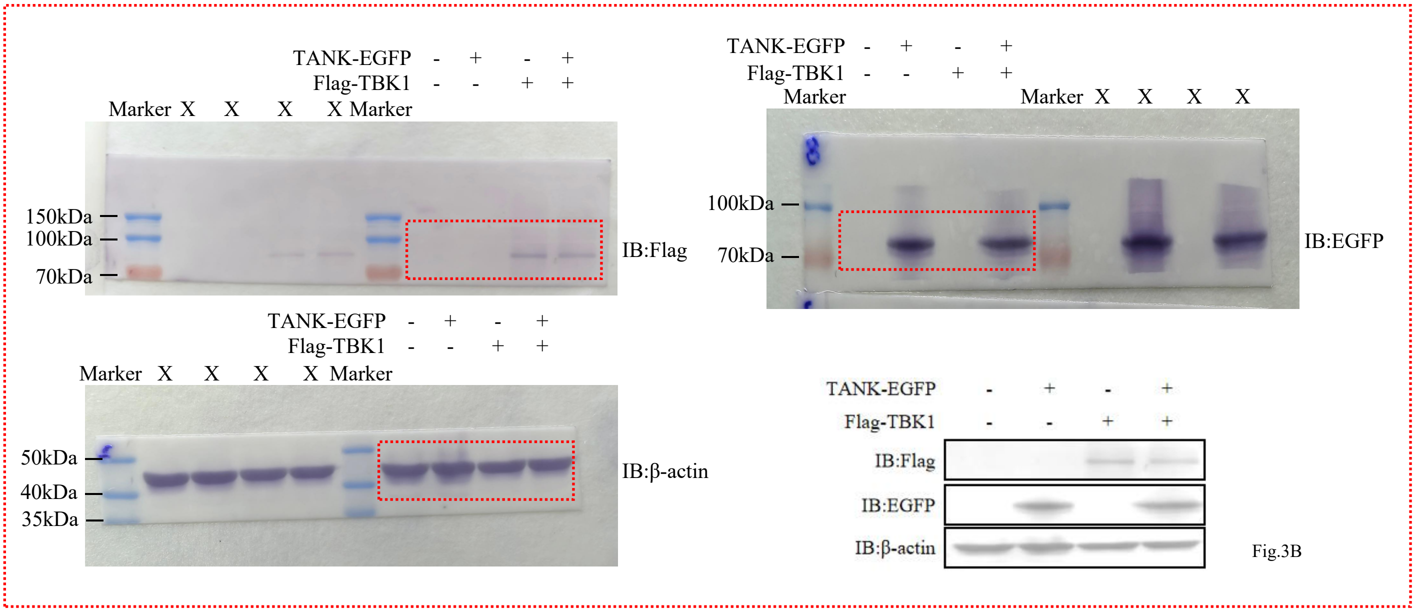

Fig.3B

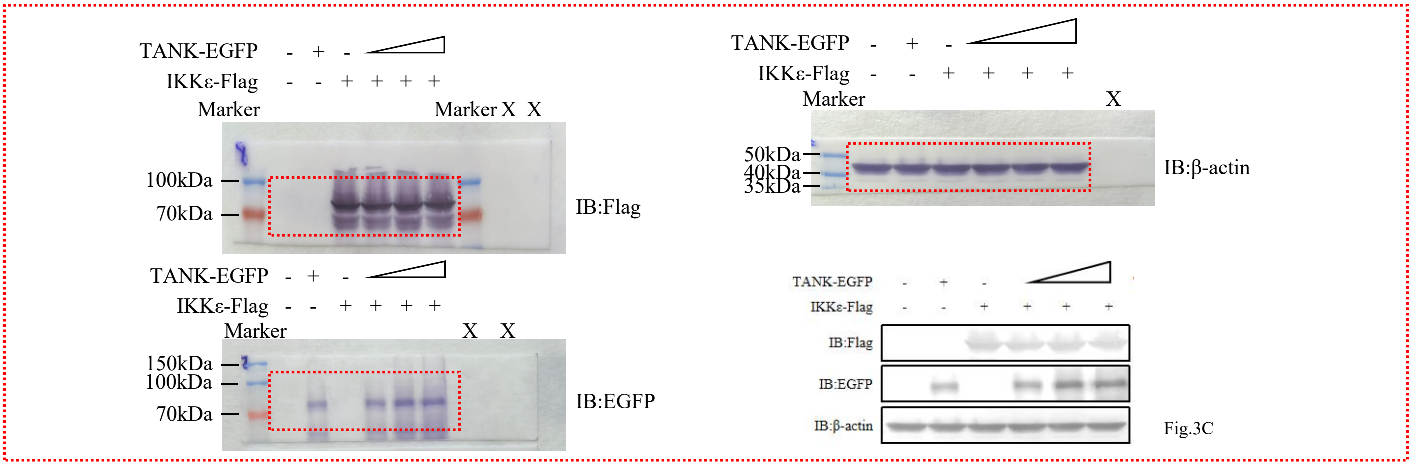

Fig.3C

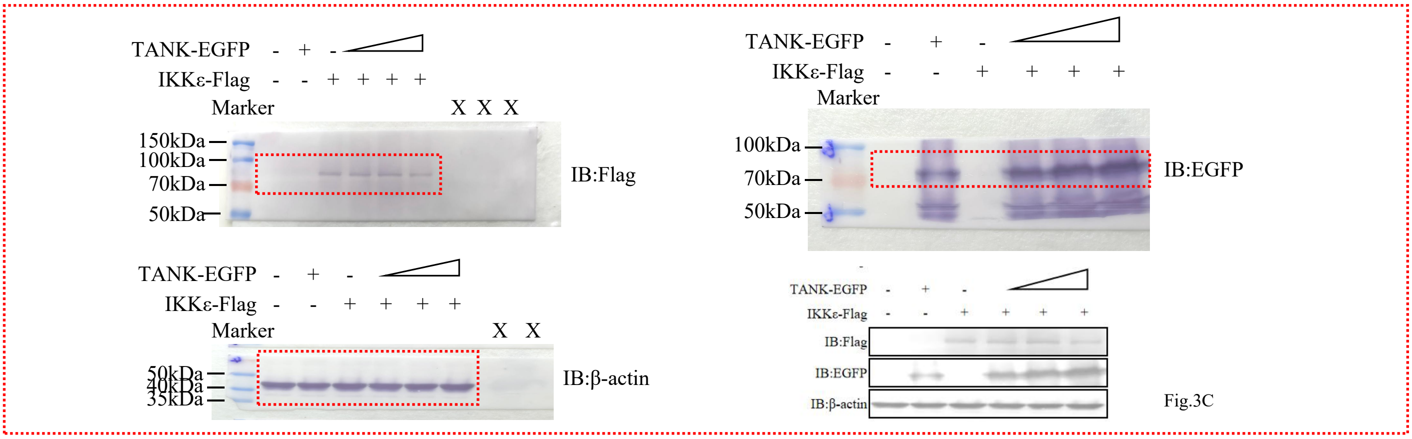

Fig.3C

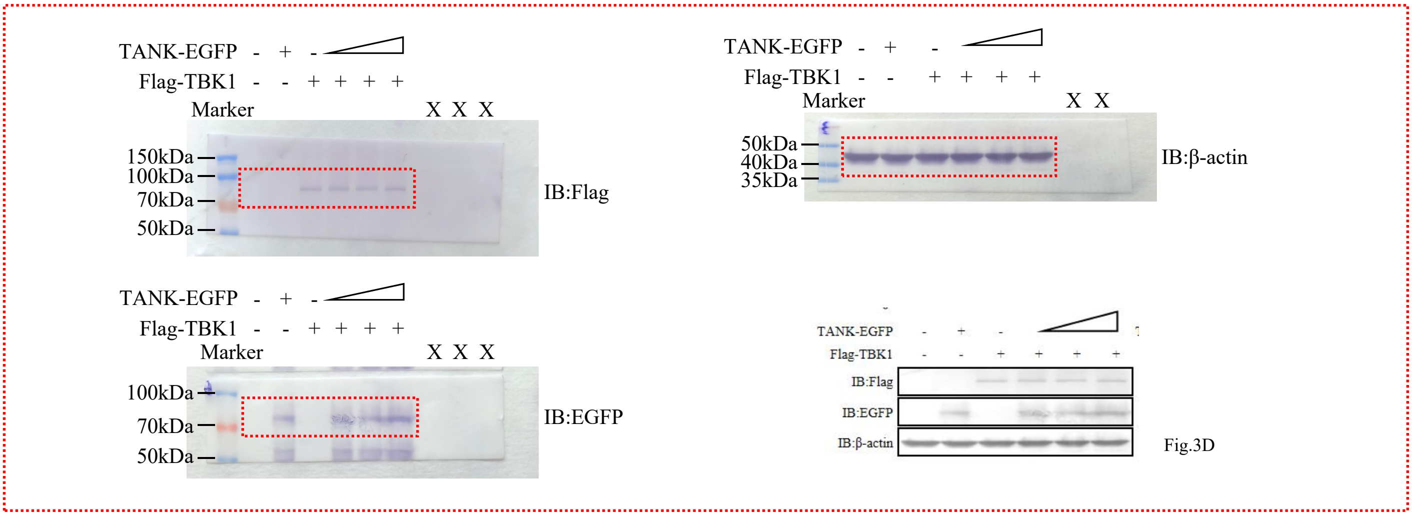

Fig.3D

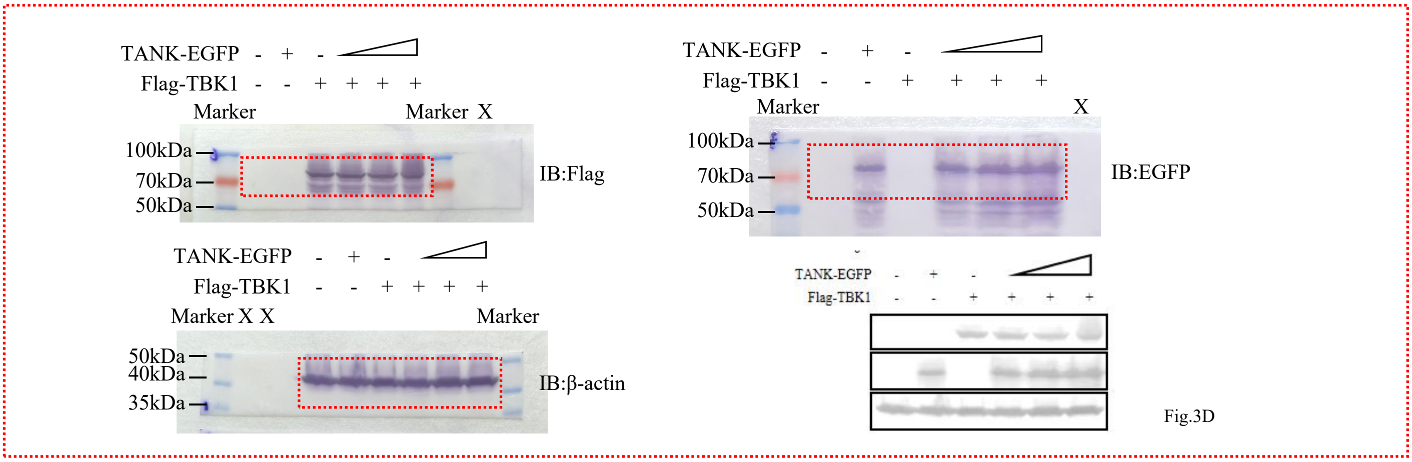

Fig.3D

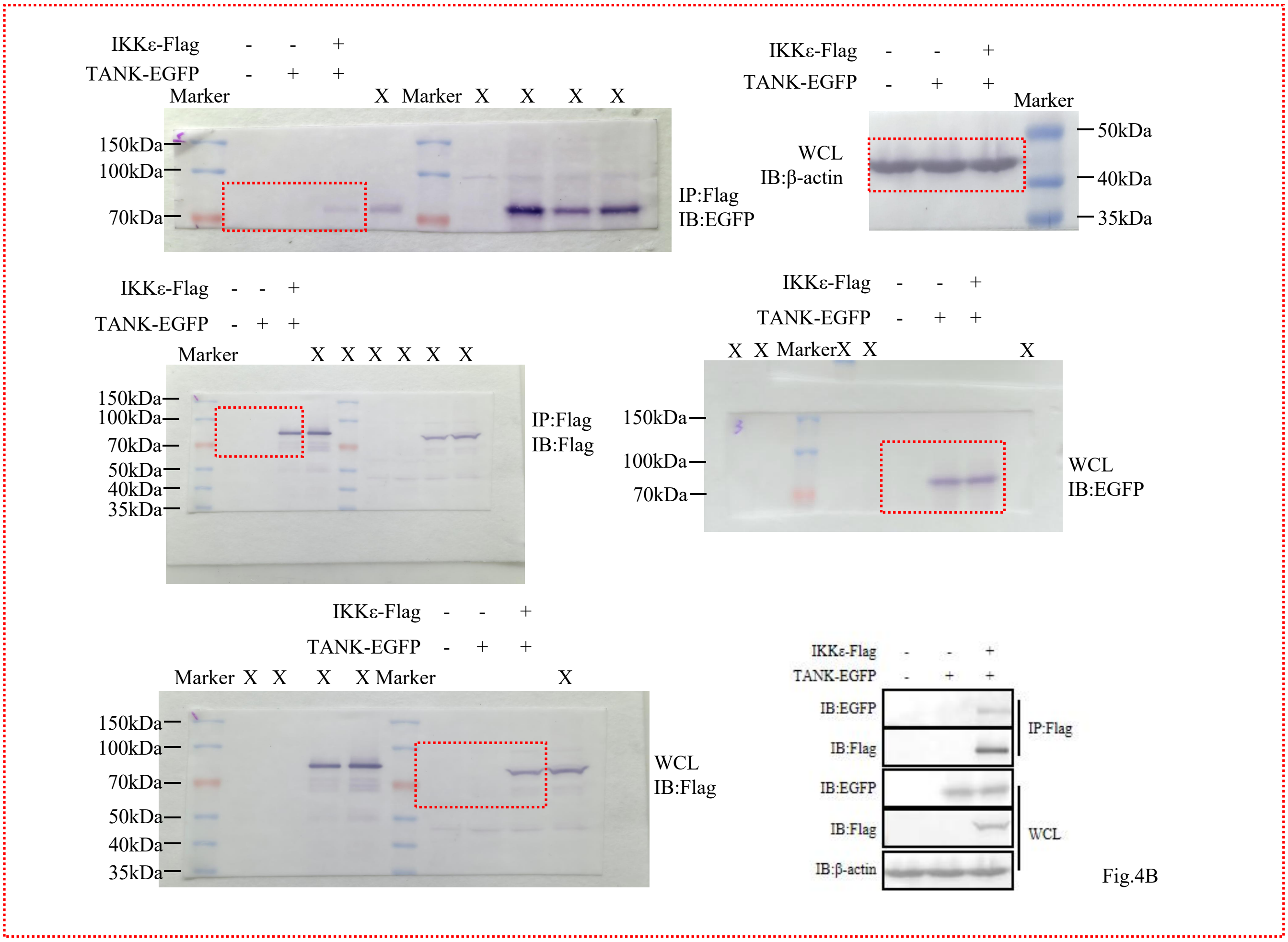
IKKε-Flag
TANK-EGFP
Marker
IP:Flag
IB:EGFP
WCL
IB:β-actin
IB:Flag
150kDa
100kDa
70kDa
50kDa
40kDa
35kDa
IP:Flag
WCL
Fig.4B

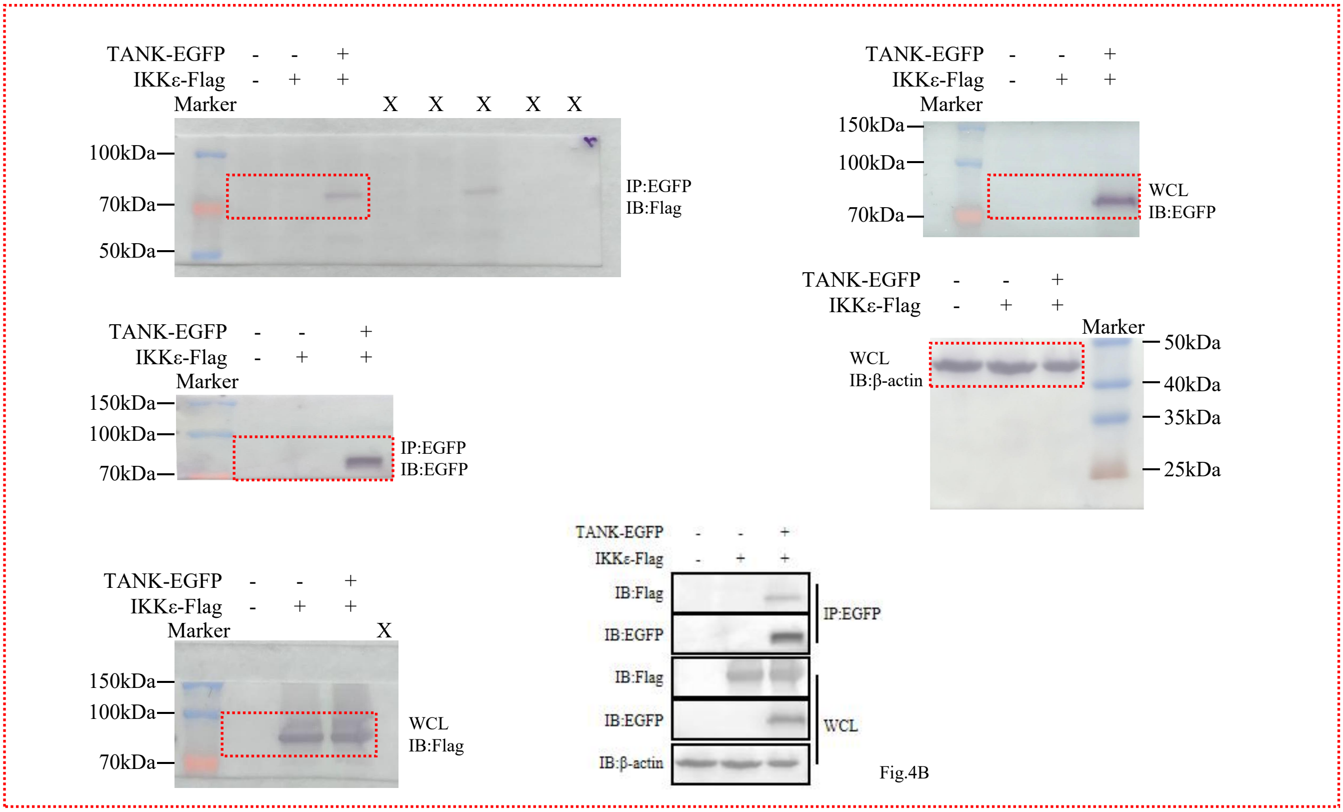
TANK-EGFP
IKKε-Flag
Marker
IP:EGFP
IB:Flag
IB:EGFP
WCL
IB:β-actin
150kDa
100kDa
70kDa
50kDa
40kDa
35kDa
25kDa
Fig.4B

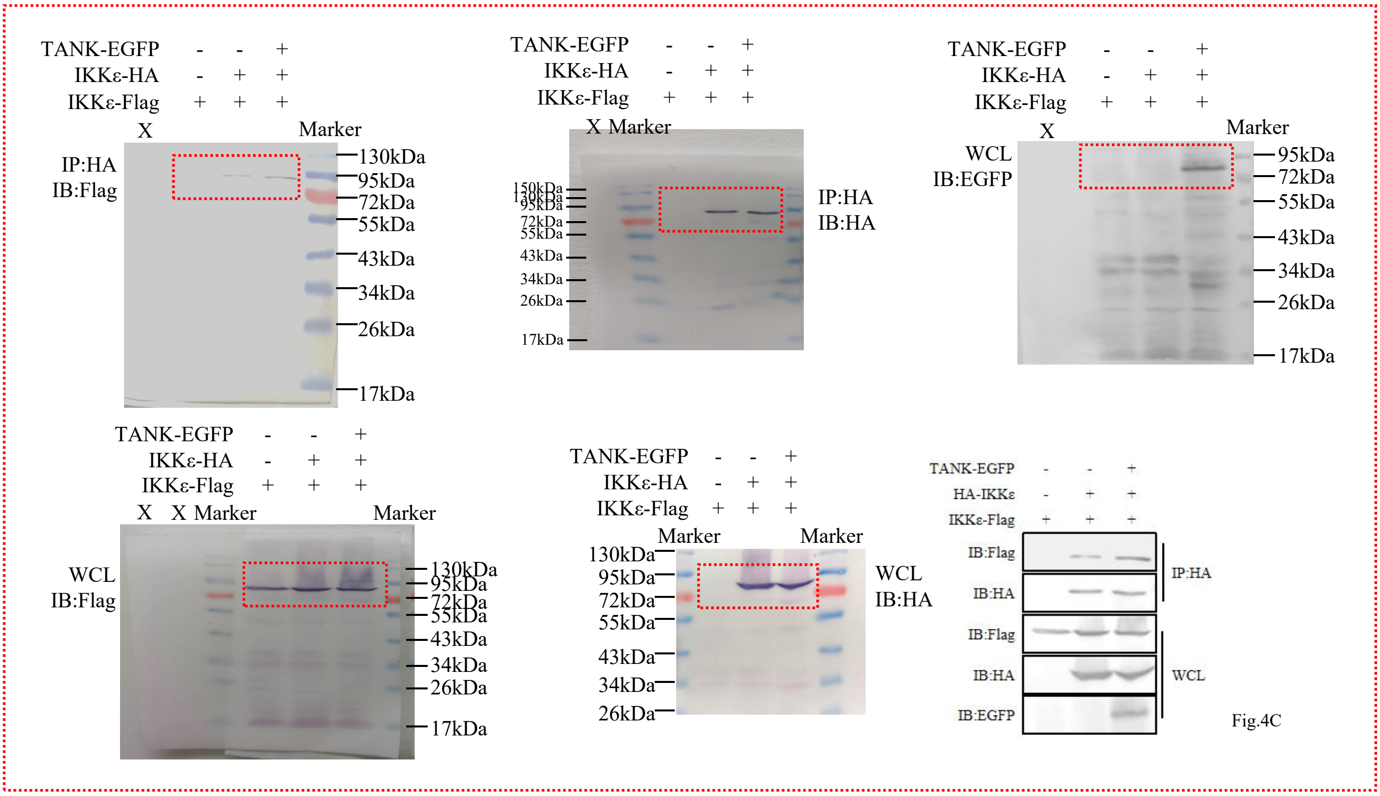
TANK-EGFP - - +
IKKε-HA - + +
IKKε-Flag + + +
X
Marker
IP:HA
IB:Flag
130kDa
95kDa
72kDa
55kDa
43kDa
34kDa
26kDa
17kDa
TANK-EGFP - - +
IKKε-HA - + +
IKKε-Flag + + +
X Marker
IP:HA
IB:HA
150kDa
130kDa
95kDa
72kDa
55kDa
43kDa
34kDa
26kDa
17kDa
TANK-EGFP - - +
IKKε-HA - + +
IKKε-Flag + + +
X
Marker
WCL
IB:EGFP
95kDa
72kDa
55kDa
43kDa
34kDa
26kDa
17kDa
TANK-EGFP - - +
IKKε-HA - + +
IKKε-Flag + + +
X X Marker
Marker
WCL
IB:Flag
130kDa
95kDa
72kDa
55kDa
43kDa
34kDa
26kDa
17kDa
TANK-EGFP - - +
IKKε-HA - + +
IKKε-Flag + + +
Marker
Marker
WCL
IB:HA
130kDa
95kDa
72kDa
55kDa
43kDa
34kDa
26kDa
TANK-EGFP - - +
HA-IKKε - + +
IKKε-Flag + + +
IB:Flag
IB:HA
IP:HA
IB:Flag
IB:HA
IB:EGFP
WCL
Fig.4C

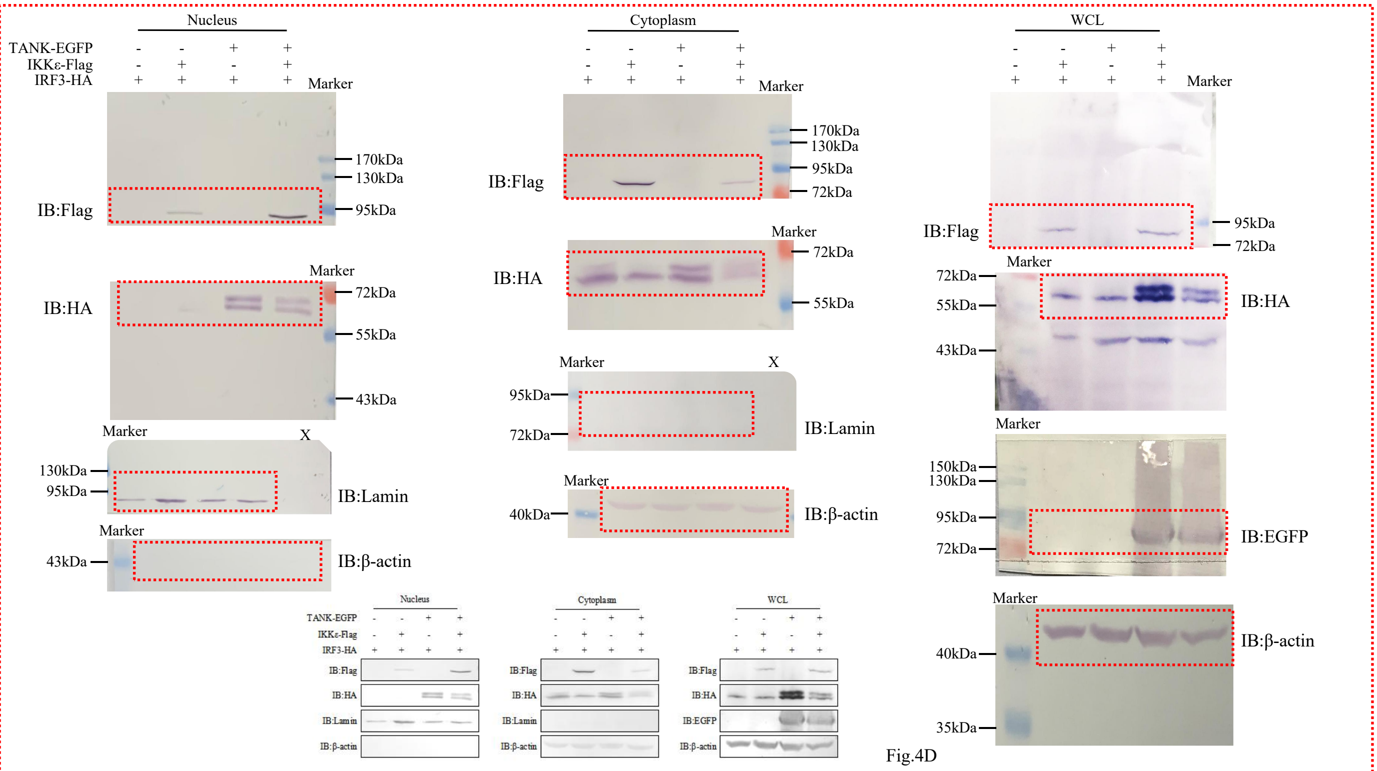
Nucleus
Cytoplasm
WCL
TANK-EGFP
IKKε-Flag
IRF3-HA
Marker
IB:Flag
IB:HA
IB:Lamin
IB:β-actin
IB:EGFP
170kDa
130kDa
150kDa
95kDa
72kDa
55kDa
43kDa
40kDa
35kDa
X

Fig.4D

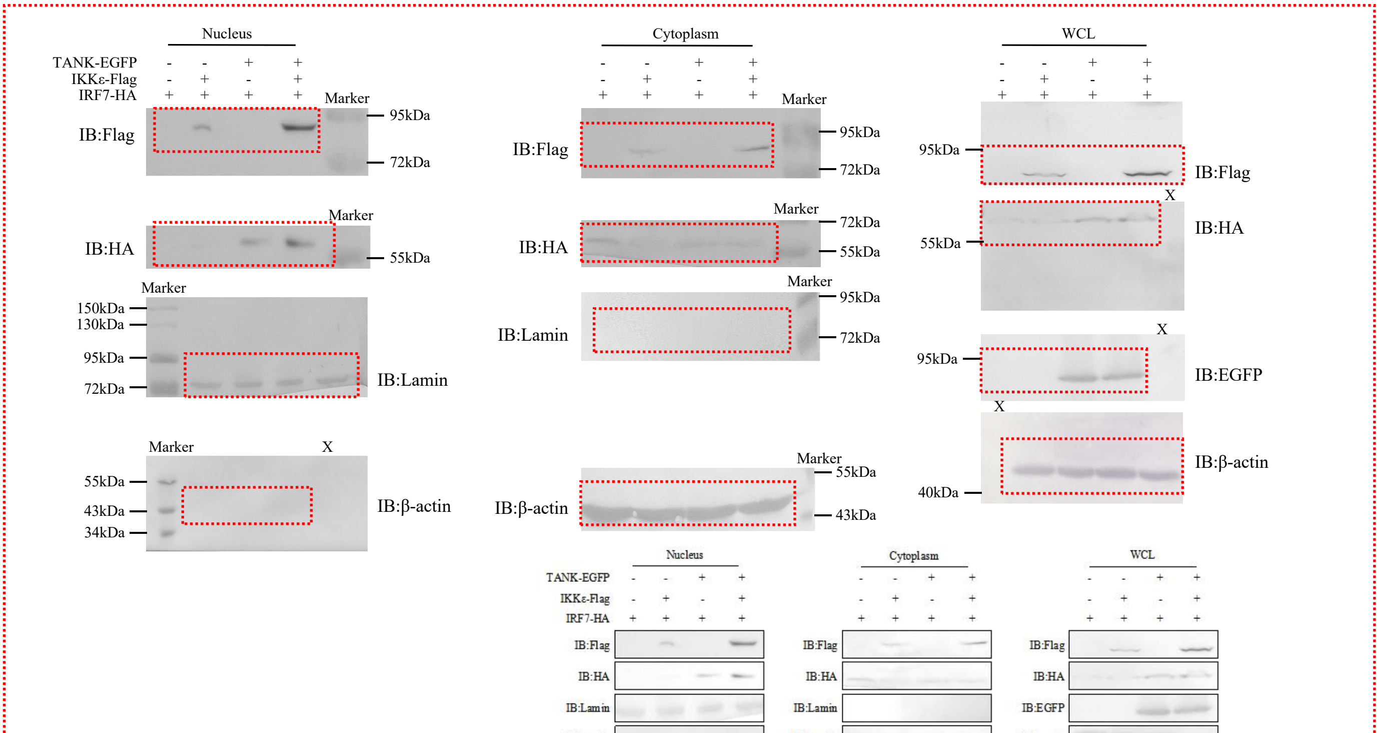
Nucleus
Cytoplasm
WCL
TANK-EGFP
IKKε-Flag
IRF7-HA
Marker
IB:Flag
IB:HA
IB:Lamin
IB:β-actin
IB:EGFP
150kDa
130kDa
95kDa
72kDa
55kDa
43kDa
40kDa
34kDa
X

Fig.4E

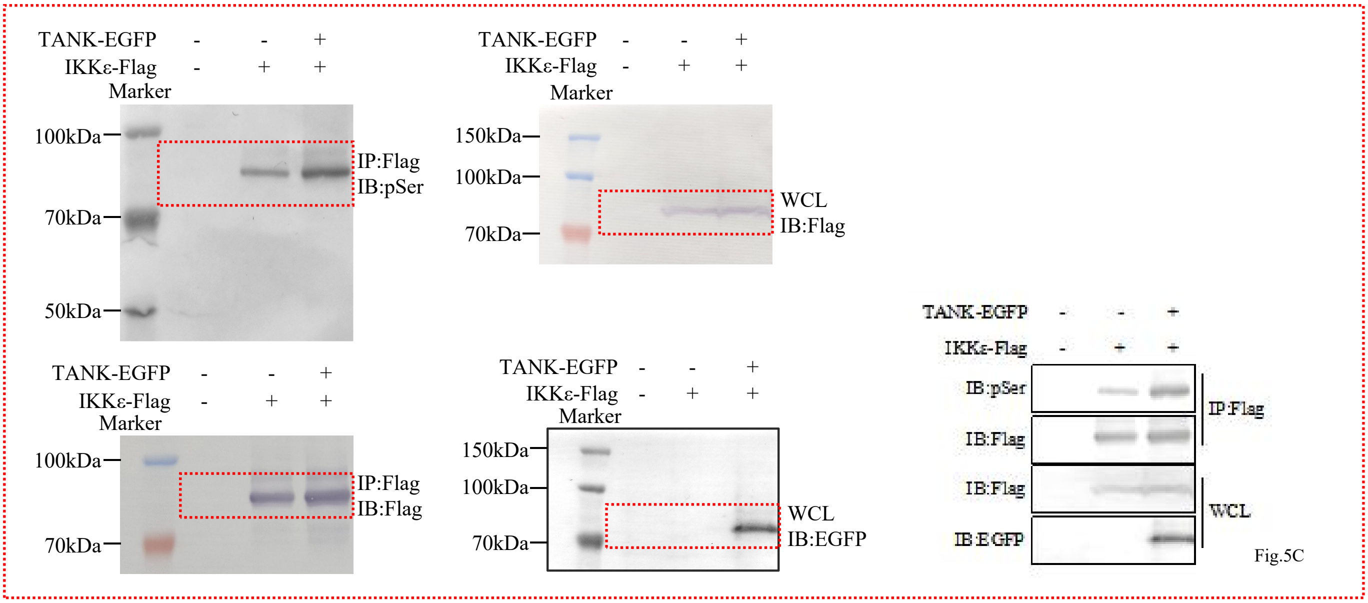
TANK-EGFP - - +
IKKε-Flag - + +
Marker
100kDa
70kDa
50kDa
IP:Flag
IB:pSer
150kDa
WCL
IB:Flag
IP:Flag
IB:Flag
WCL
IB:EGFP
IB:pSer
IB:Flag
IB:Flag
IB:EGFP
IP:Flag
WCL
Fig.5C

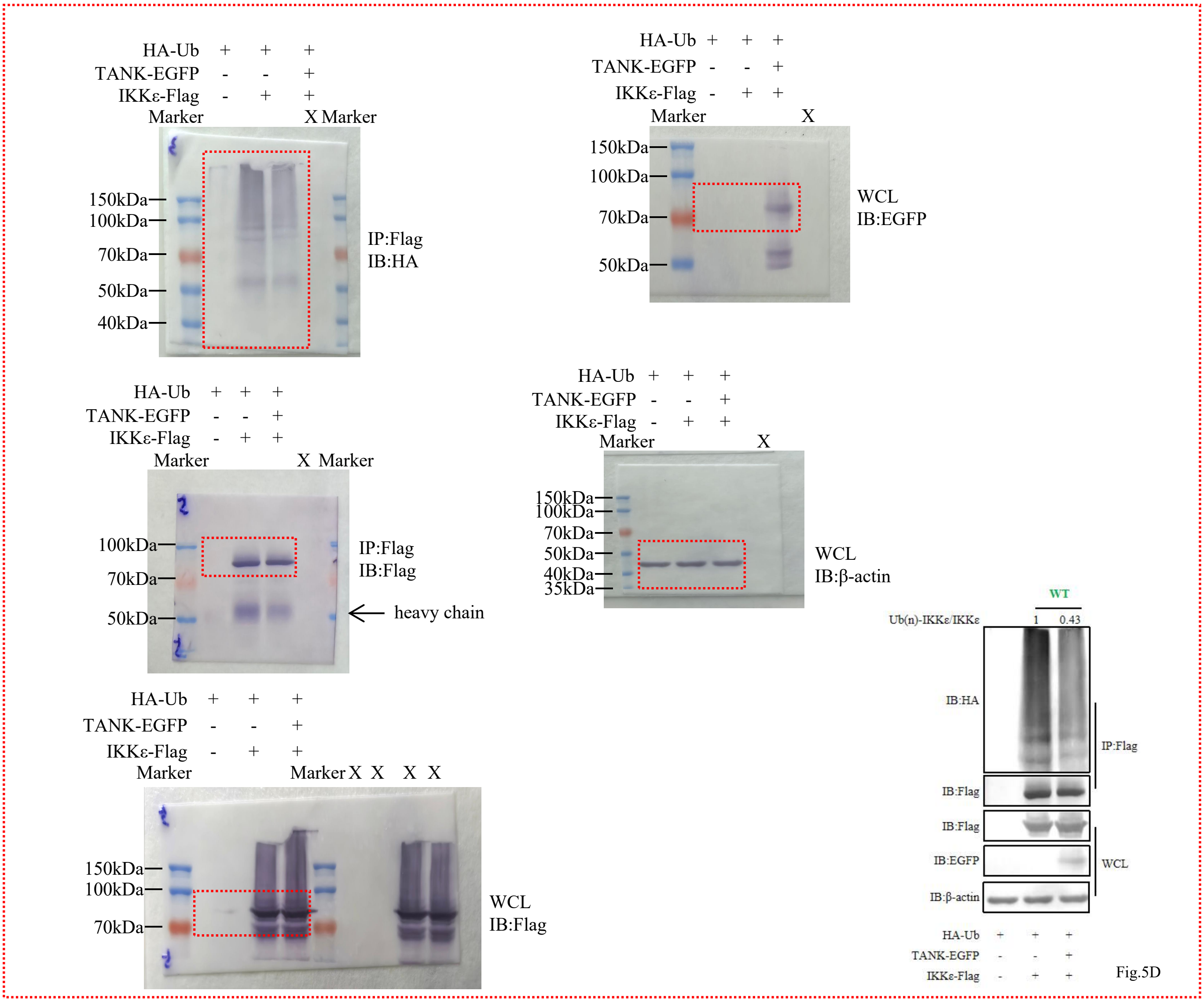
HA-Ub + + +
TANK-EGFP - - +
IKKε-Flag - + +
Marker
X Marker
150kDa
100kDa
70kDa
50kDa
40kDa
35kDa
IP:Flag
IB:HA
WCL
IB:EGFP
IP:Flag
IB:Flag
heavy chain
WCL
IB:β-actin
Marker X X X X
WCL
IB:Flag
WT
Ub(n)-IKKε/IKKε
1
0.43
IB:HA
IB:Flag
IB:Flag
IB:EGFP
IB:β-actin
IP:Flag
WCL
Fig.5D

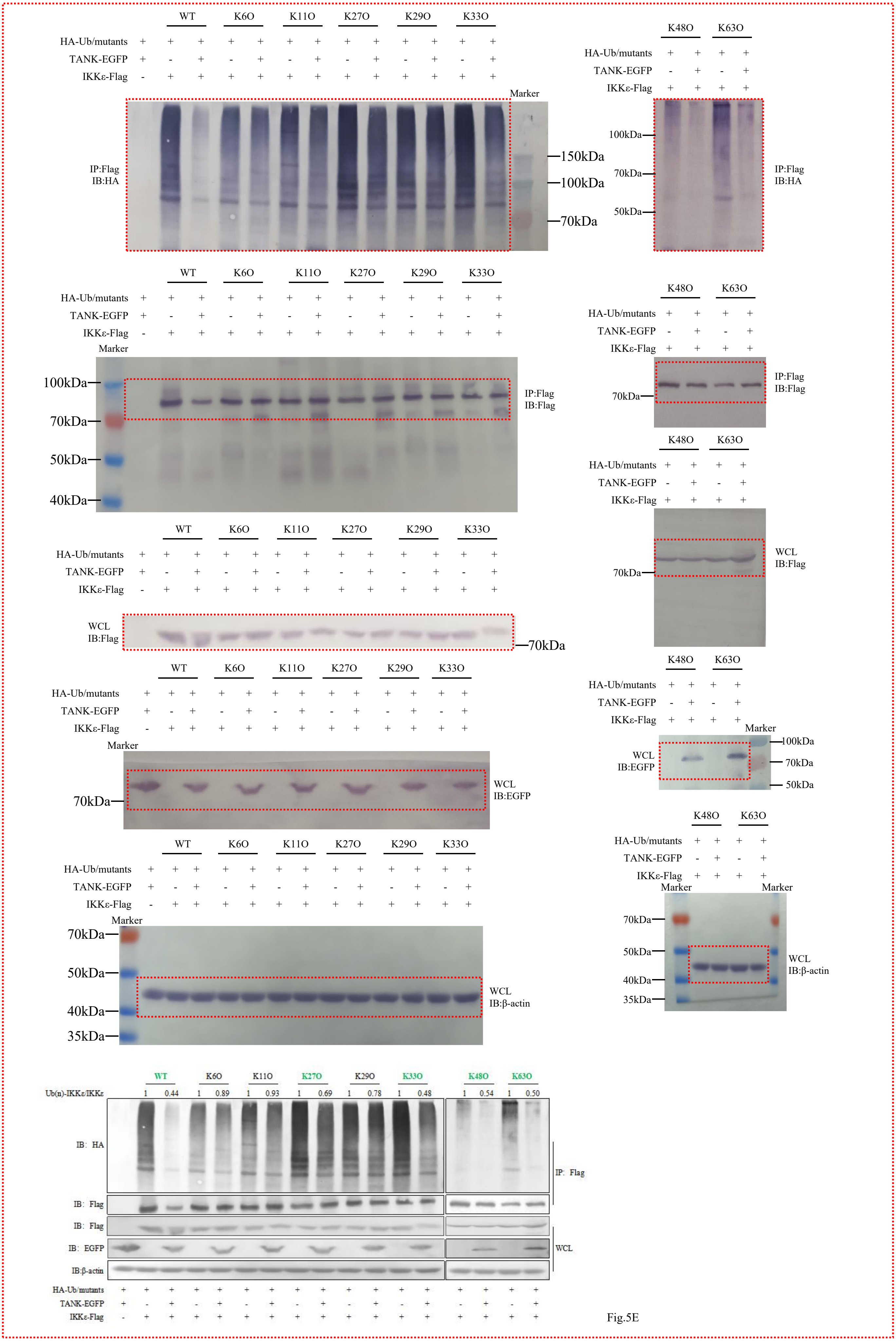
WT
K6O
K11O
K27O
K29O
K33O
K48O
K63O
HA-Ub/mutants
TANK-EGFP
IKKε-Flag
Marker
IP:Flag
IB:HA
150kDa
100kDa
70kDa
50kDa
40kDa
35kDa
IP:Flag
IB:Flag
WCL
IB:Flag
WCL
IB:EGFP
WCL
IB:β-actin
Ub(n)-IKKε/IKKε
1 0.44 1 0.89 1 0.93 1 0.69 1 0.78 1 0.48 1 0.54 1 0.50
IB: HA
IB: Flag
IB: EGFP
IB:β-actin
IP: Flag
WCL

Fig.5E

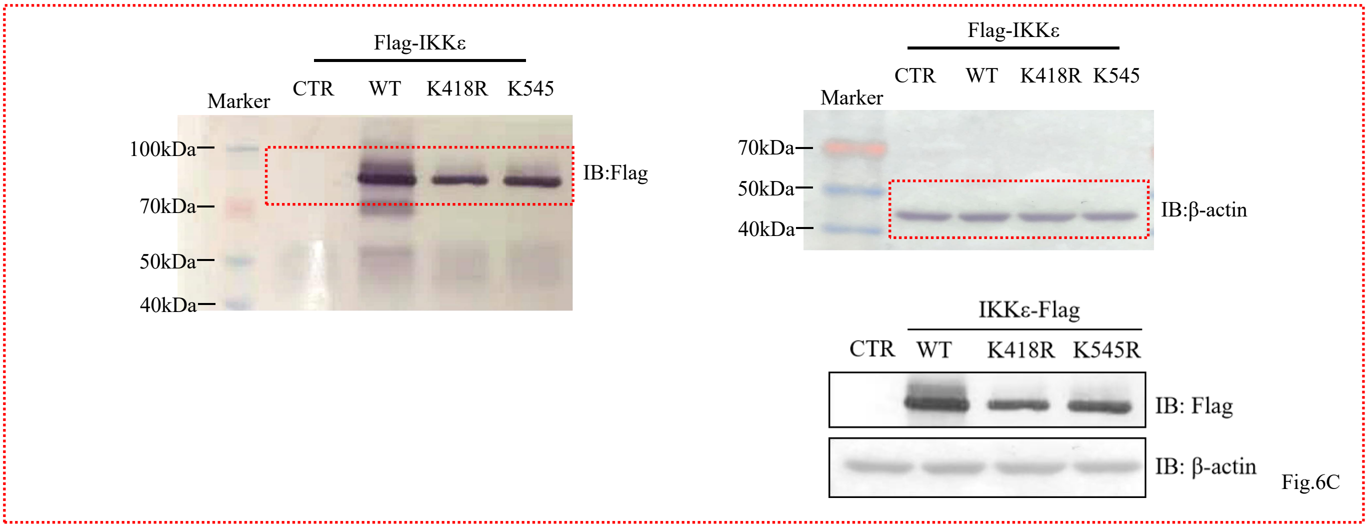

Fig.6C

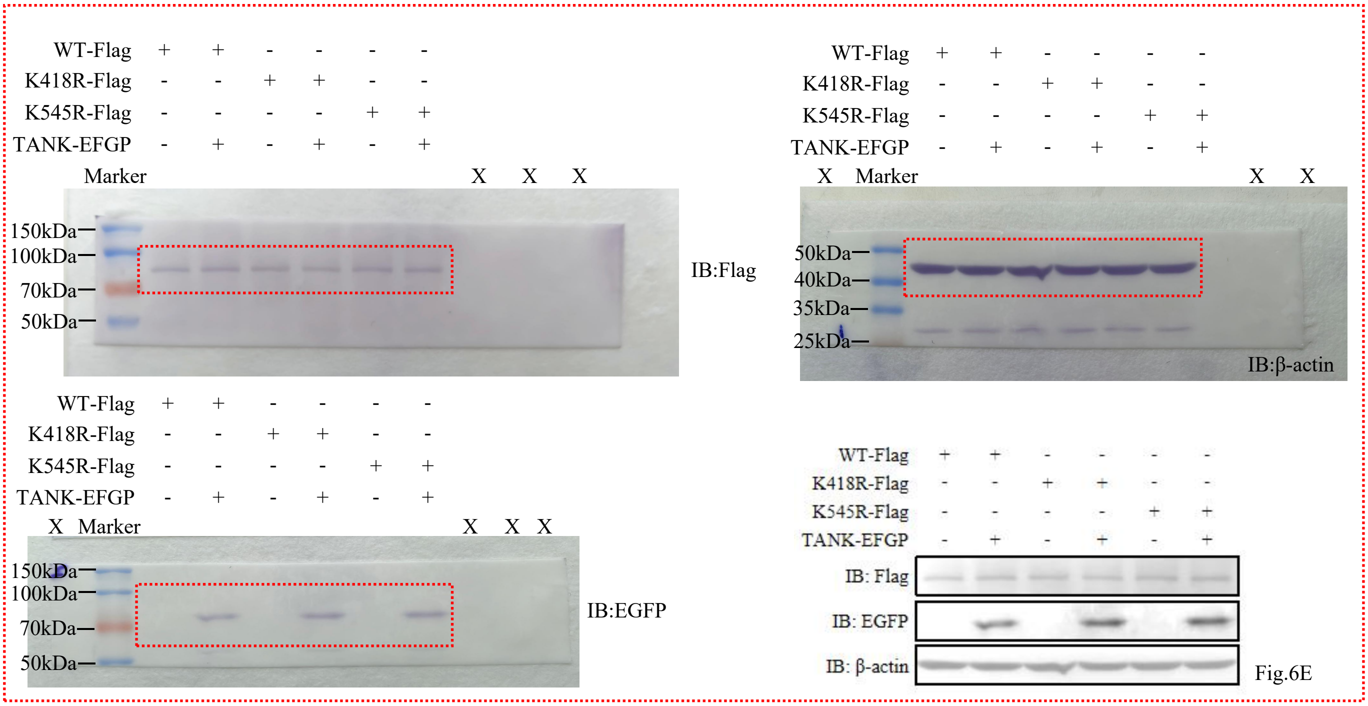

Fig.6E

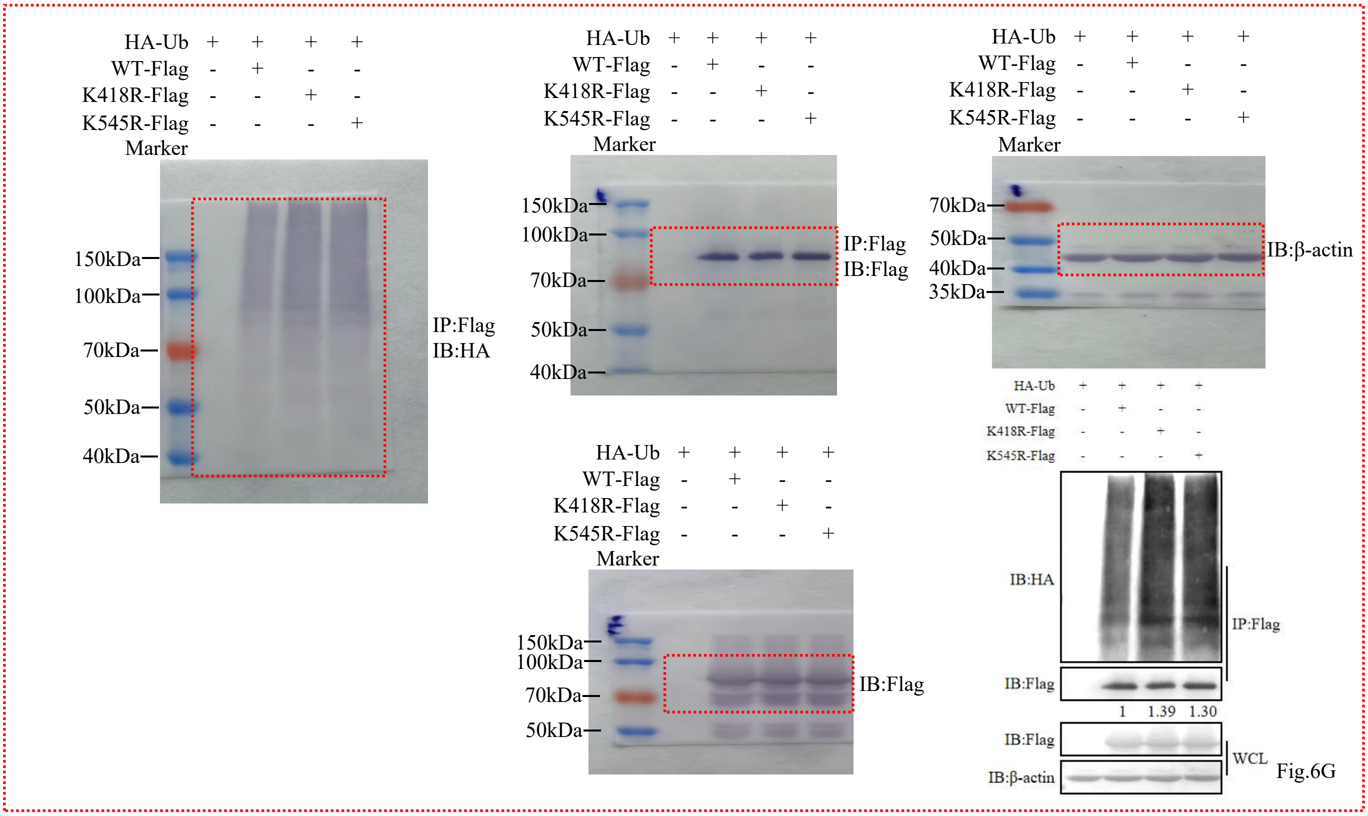

Fig.6G

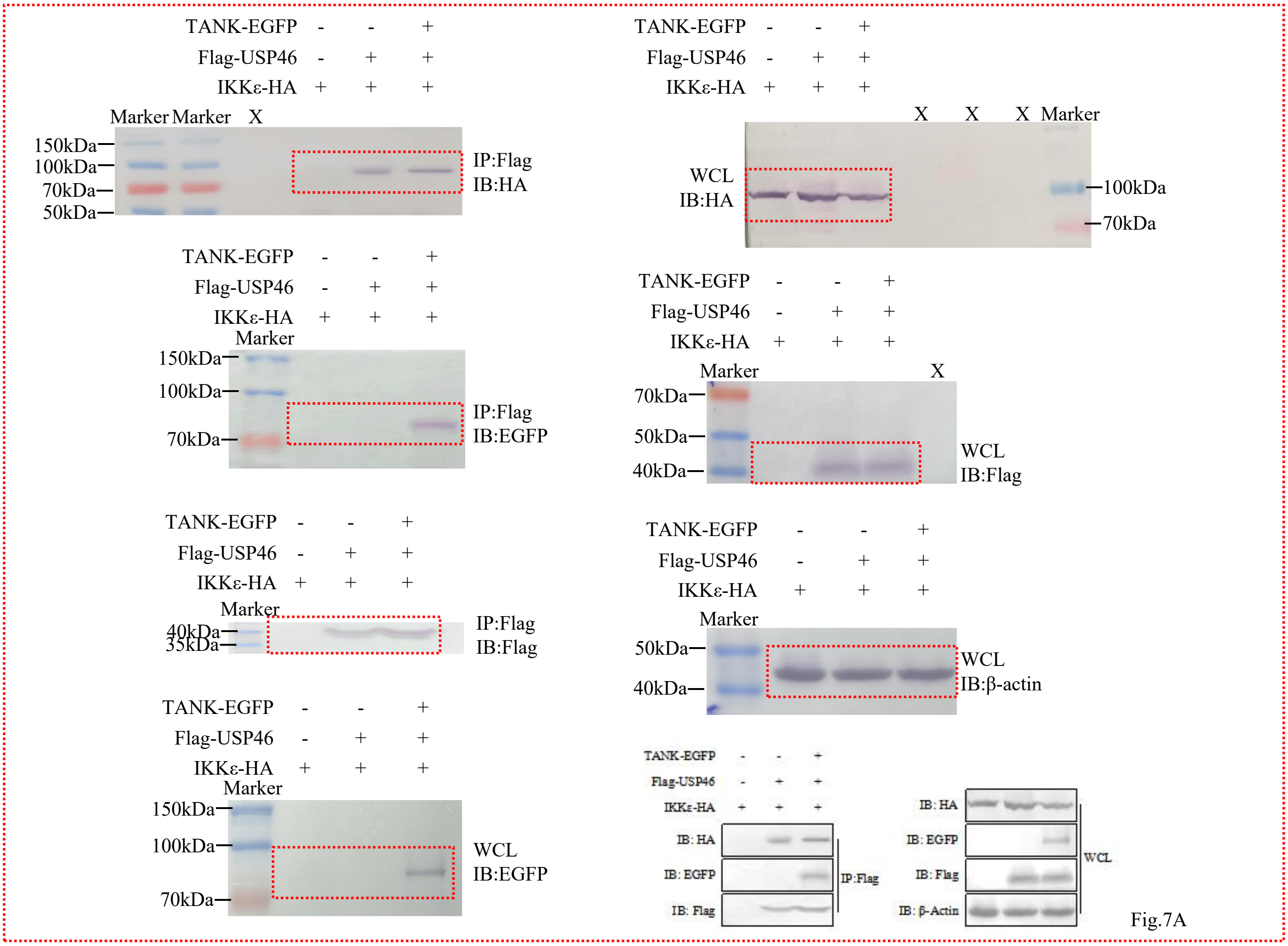
TANK-EGFP - - +
Flag-USP46 - + +
IKKε-HA + + +
Marker Marker X
150kDa
100kDa
70kDa
50kDa
IP:Flag
IB:HA
X X X Marker
WCL
IB:HA
100kDa
70kDa
IP:Flag
IB:EGFP
40kDa
35kDa
IP:Flag
IB:Flag
WCL
IB:Flag
50kDa
WCL
IB:β-actin
WCL
IB:EGFP
IB: HA
IB: EGFP
IB: Flag
IP:Flag
IB: β-Actin
WCL
Fig.7A

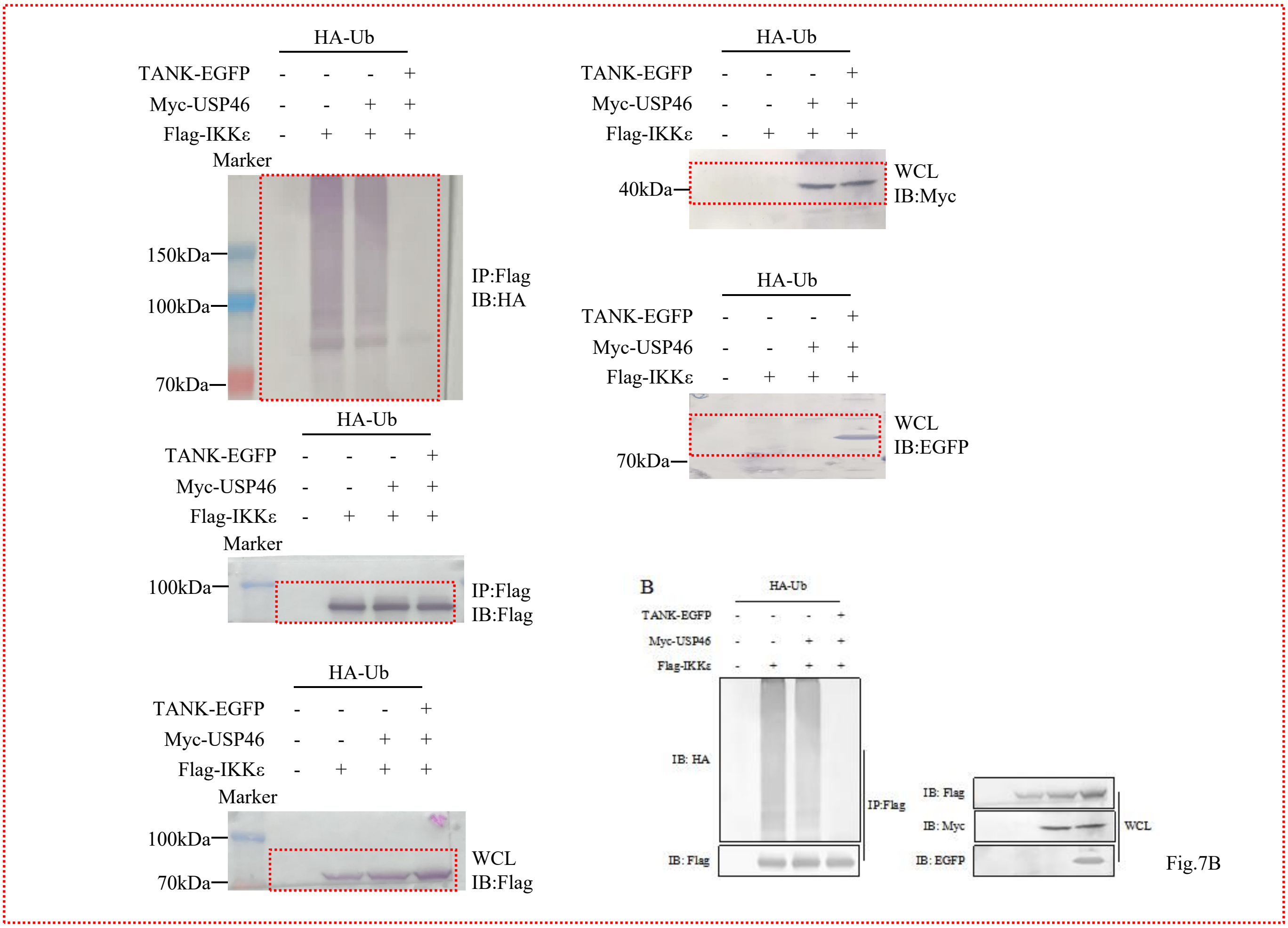
HA-Ub
TANK-EGFP - - - +
Myc-USP46 - - + +
Flag-IKKε - + + +
Marker
150kDa
100kDa
70kDa
40kDa
IP:Flag
IB:HA
WCL
IB:Myc
WCL
IB:EGFP
IP:Flag
IB:Flag
WCL
IB:Flag
B
IB: HA
IB: Flag
IP:Flag
IB: Myc
IB: EGFP
WCL
Fig.7B

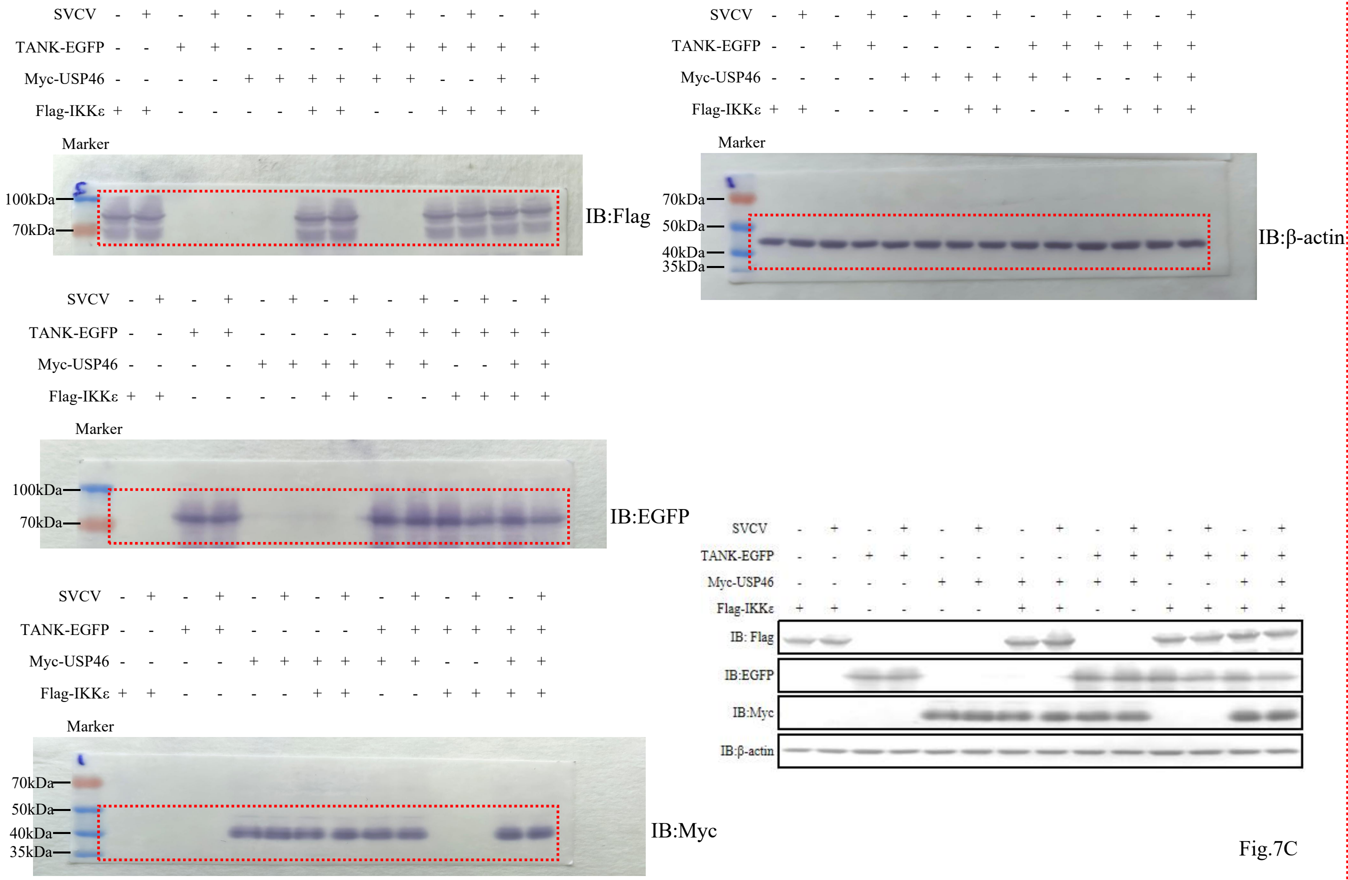
SVCV - + - + - + - + - + - + - +
TANK-EGFP - - + + - - - - + + + + + +
Myc-USP46 - - - - + + + + + + - - + +
Flag-IKKε + + - - - - + + - - + + + +
Marker
100kDa
70kDa
IB:Flag
50kDa
40kDa
35kDa
IB:β-actin
IB:EGFP
IB:Myc
IB: Flag
IB:EGFP
IB:Myc
IB:β-actin

Fig.7C

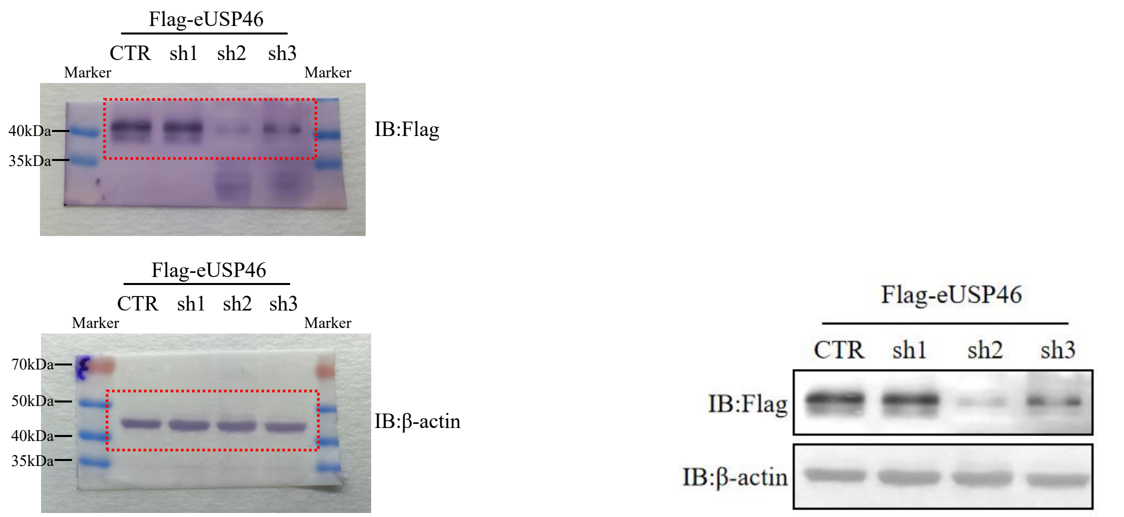
Flag-eUSP46
CTR sh1 sh2 sh3
Marker
Marker
40kDa
35kDa
IB:Flag
70kDa
50kDa
IB:β-actin

Fig.7E

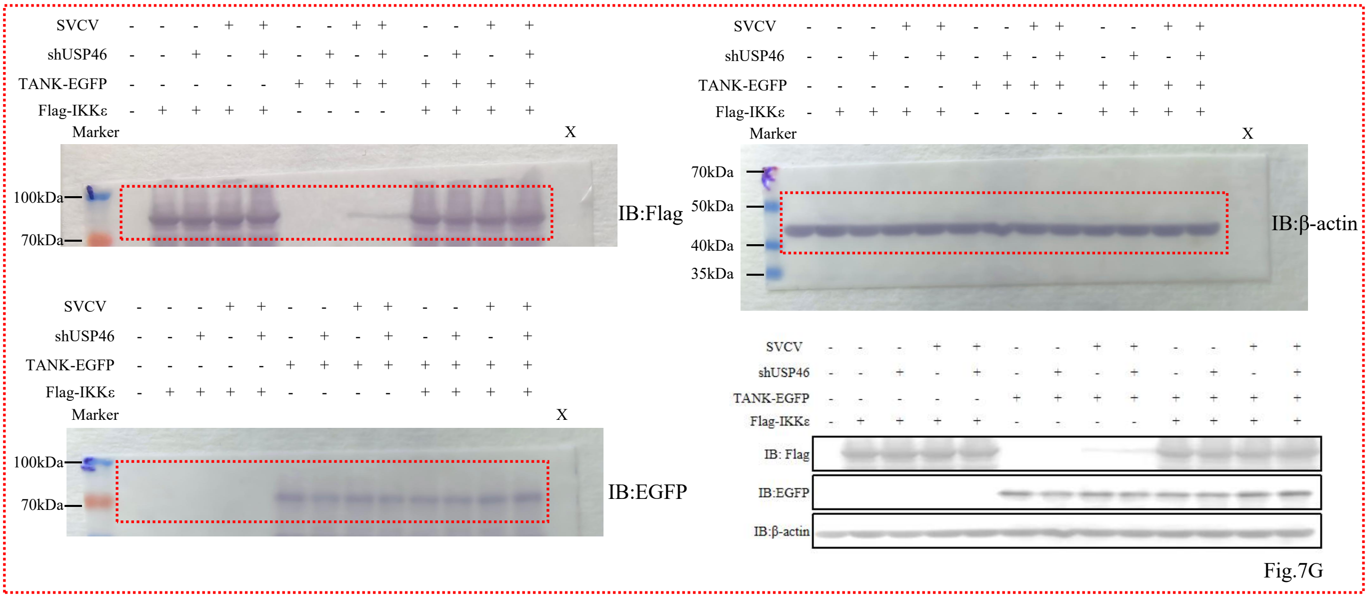
SVCV
shUSP46
TANK-EGFP
Flag-IKKε
Marker
X
100kDa
70kDa
IB:Flag
50kDa
40kDa
35kDa
IB:β-actin
IB:EGFP
IB: Flag
IB:EGFP
IB:β-actin

Fig.7G

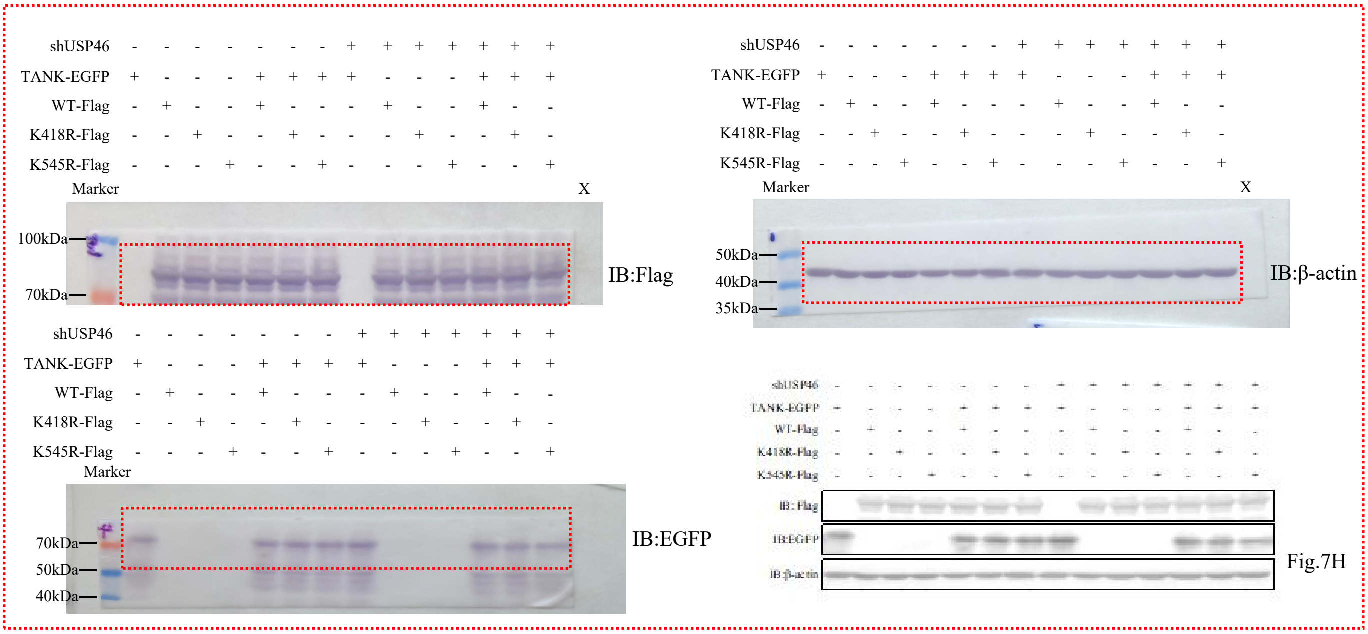
shUSP46
TANK-EGFP
WT-Flag
K418R-Flag
K545R-Flag
Marker
X
100kDa
70kDa
IB:Flag
50kDa
40kDa
35kDa
IB:β-actin
IB:EGFP
IB: Flag
IB:EGFP
IB:β-actin

Fig.7H

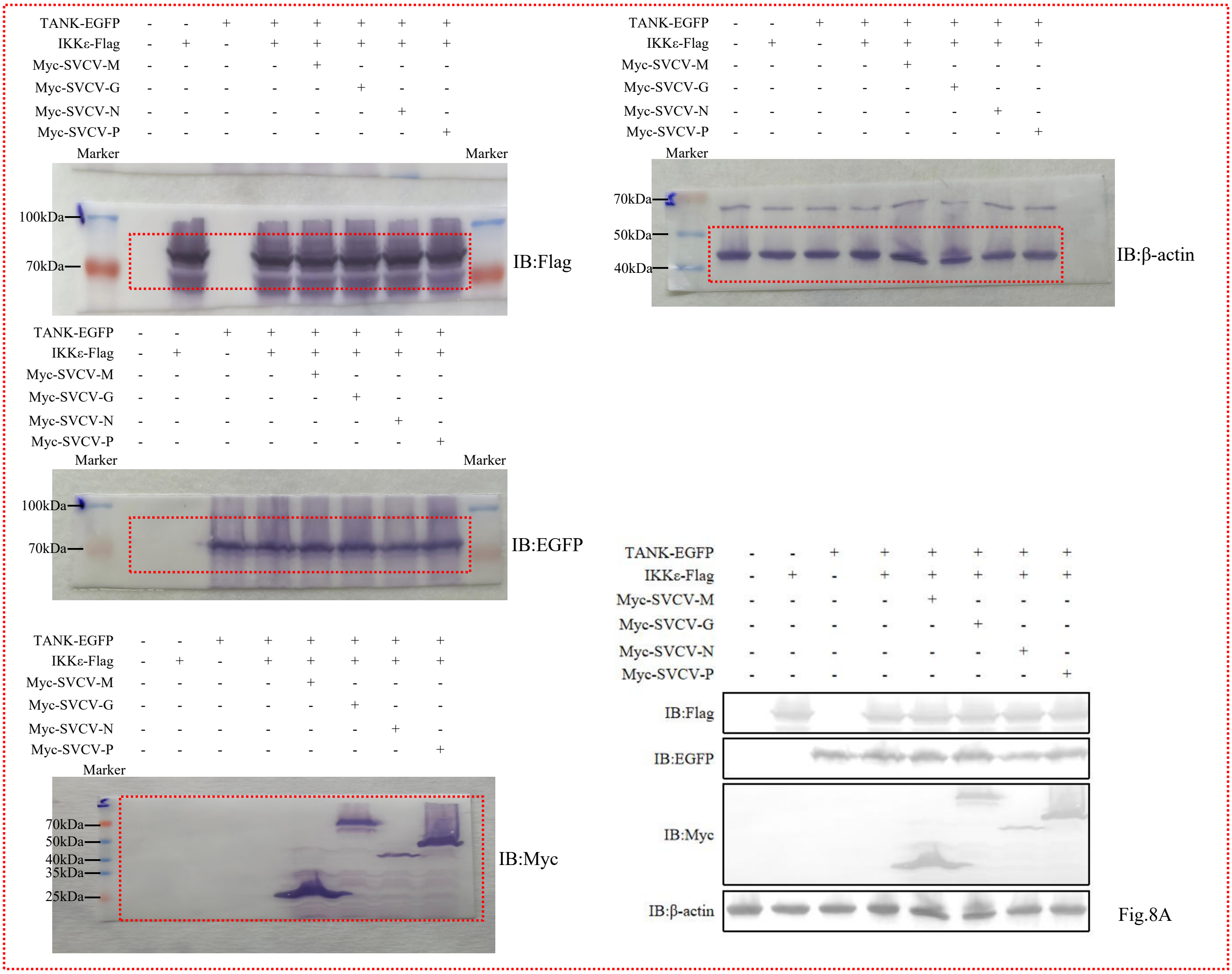
TANK-EGFP
IKKε-Flag
Myc-SVCV-M
Myc-SVCV-G
Myc-SVCV-N
Myc-SVCV-P
Marker
100kDa
70kDa
50kDa
40kDa
35kDa
25kDa
IB:Flag
IB:β-actin
IB:EGFP
IB:Myc
Fig.8A

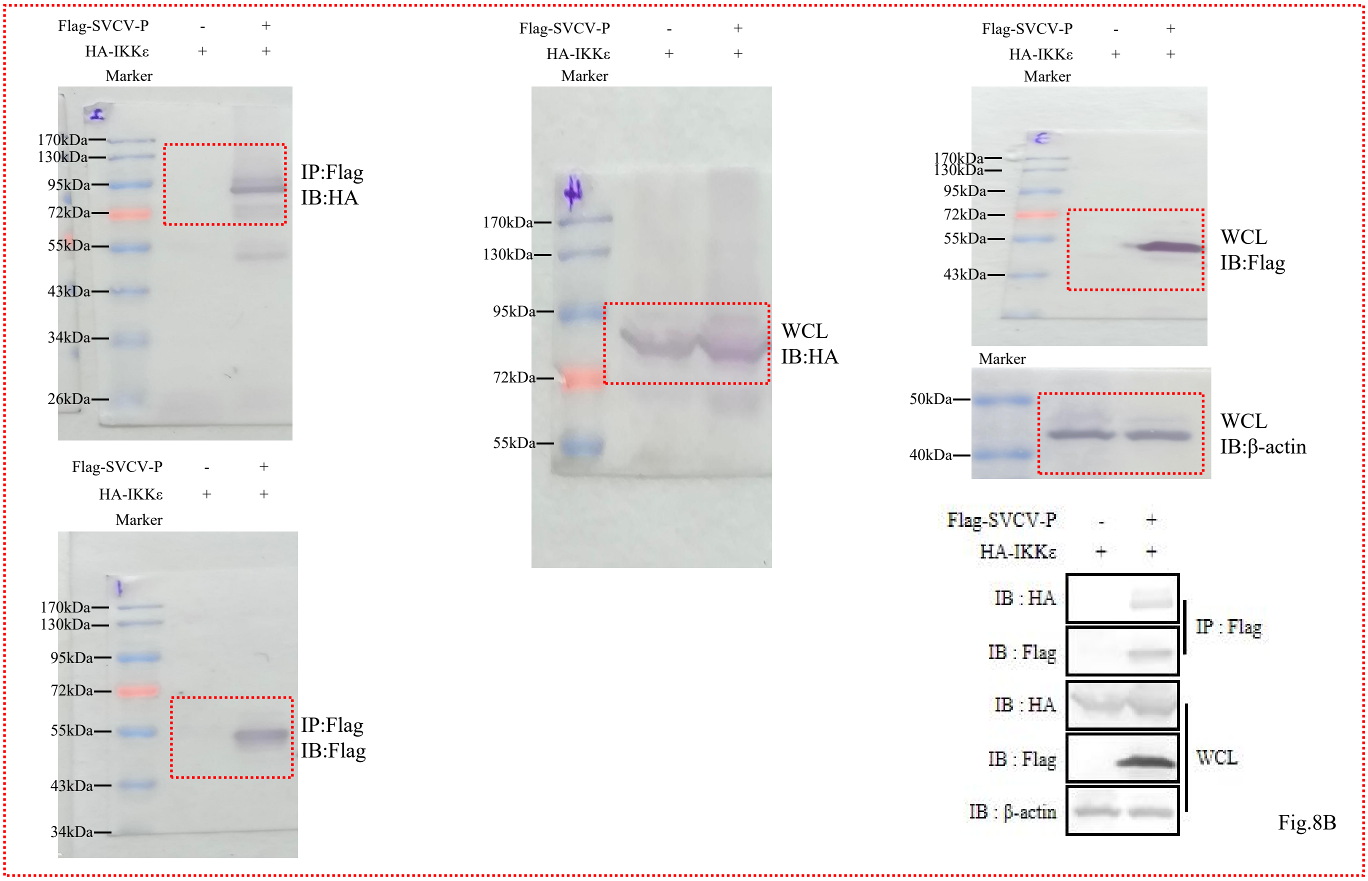
Flag-SVCV-P
HA-IKKε
Marker
170kDa
130kDa
95kDa
72kDa
55kDa
43kDa
34kDa
26kDa
50kDa
40kDa
IP:Flag
IB:HA
IB:Flag
WCL
IB:β-actin
IB : HA
IB : Flag
IB : β-actin
IP : Flag
Fig.8B

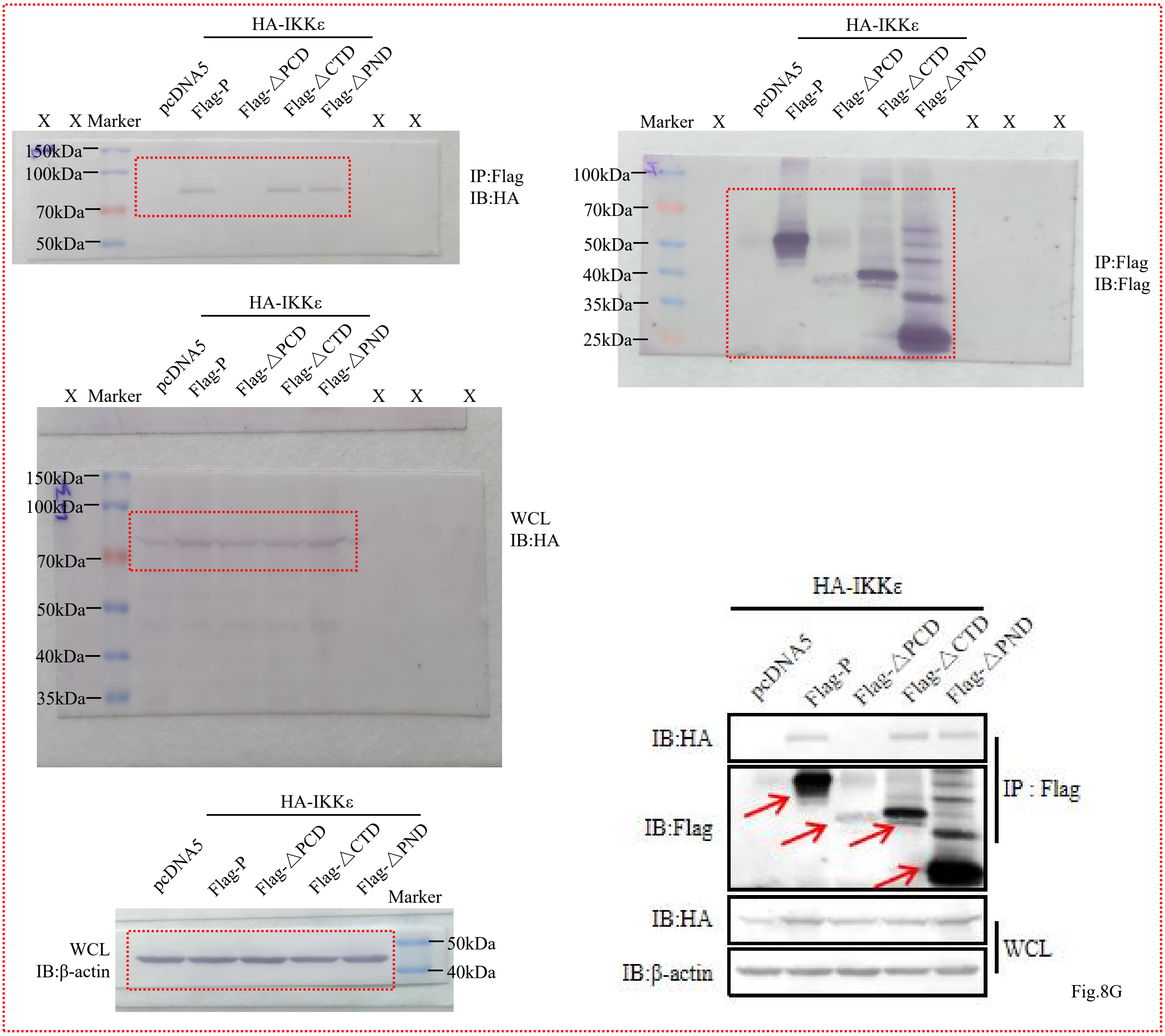
HA-IKKε
pcDNA5
Flag-P
Flag-△PCD
Flag-△CTD
Flag-△PND
X
X Marker
X
X
150kDa
100kDa
70kDa
50kDa
IP:Flag
IB:HA
Marker
X
X
X
X
100kDa
70kDa
50kDa
40kDa
35kDa
25kDa
IP:Flag
IB:Flag
X Marker
X
X
X
150kDa
100kDa
70kDa
50kDa
40kDa
35kDa
WCL
IB:HA
Marker
WCL
IB:β-actin
50kDa
40kDa
IB:HA
IB:Flag
IB:HA
IB:β-actin
IP : Flag
WCL
Fig.8G

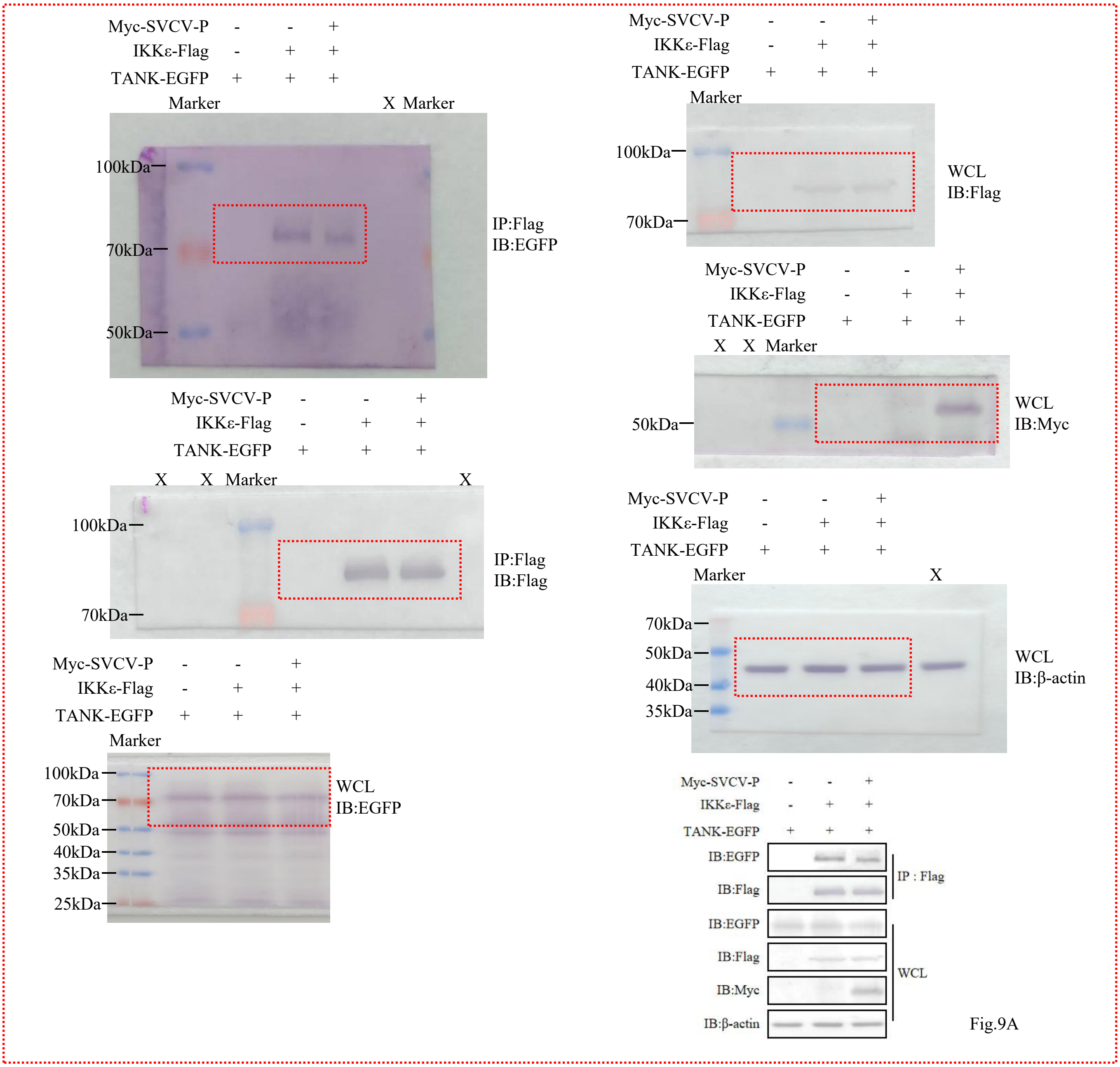
Myc-SVCV-P - - +
IKKε-Flag - + +
TANK-EGFP + + +
Marker
X Marker
100kDa
70kDa
50kDa
IP:Flag
IB:EGFP
X X Marker
X
IB:Flag
WCL
IB:EGFP
40kDa
35kDa
25kDa
IB:Myc
IB:β-actin
IP : Flag
WCL

Fig.9A

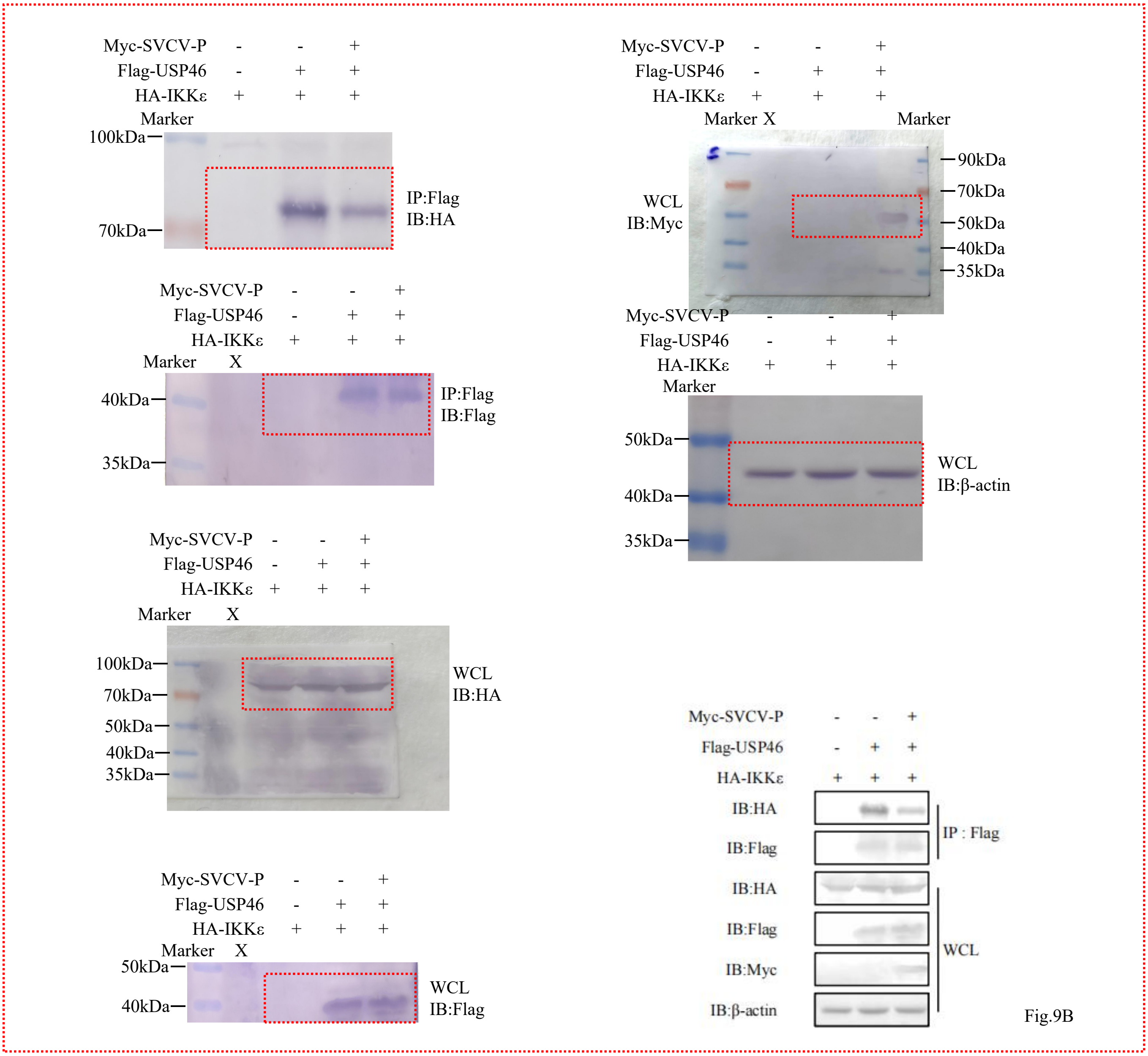
Myc-SVCV-P - - +
Flag-USP46 - + +
HA-IKKε + + +
Marker
100kDa
70kDa
IP:Flag
IB:HA
Marker X
40kDa
35kDa
IP:Flag
IB:Flag
100kDa
70kDa
50kDa
40kDa
35kDa
WCL
IB:HA
50kDa
40kDa
WCL
IB:Flag
Marker X Marker
90kDa
70kDa
50kDa
40kDa
35kDa
WCL
IB:Myc
WCL
IB:β-actin
IB:HA
IB:Flag
IP : Flag
IB:Myc
IB:β-actin
WCL
Fig.9B

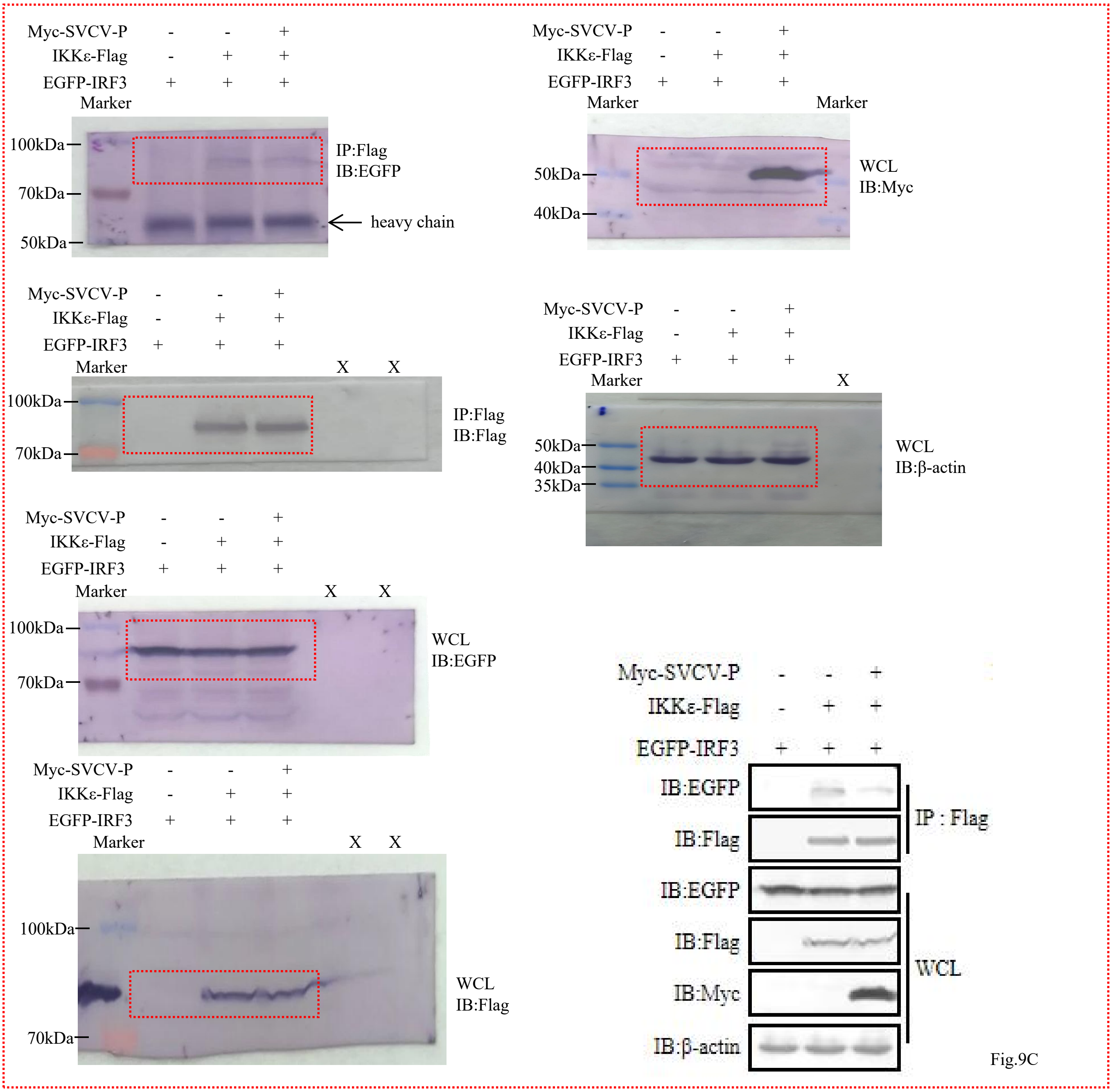
Myc-SVCV-P - - +
IKKε-Flag - + +
EGFP-IRF3 + + +
Marker
100kDa
70kDa
50kDa
IP:Flag
IB:EGFP
heavy chain
Myc-SVCV-P - - +
IKKε-Flag - + +
EGFP-IRF3 + + +
Marker
Marker
50kDa
40kDa
WCL
IB:Myc
Myc-SVCV-P - - +
IKKε-Flag - + +
EGFP-IRF3 + + +
Marker
X X
100kDa
70kDa
IP:Flag
IB:Flag
Myc-SVCV-P - - +
IKKε-Flag - + +
EGFP-IRF3 + + +
Marker
X
50kDa
40kDa
35kDa
WCL
IB:β-actin
Myc-SVCV-P - - +
IKKε-Flag - + +
EGFP-IRF3 + + +
Marker
X X
100kDa
70kDa
WCL
IB:EGFP
Myc-SVCV-P - - +
IKKε-Flag - + +
EGFP-IRF3 + + +
Marker
X X
100kDa
70kDa
WCL
IB:Flag
Myc-SVCV-P - - +
IKKε-Flag - + +
EGFP-IRF3 + + +
IB:EGFP
IB:Flag
IP : Flag
IB:EGFP
IB:Flag
IB:Myc
IB:β-actin
WCL

Fig.9C

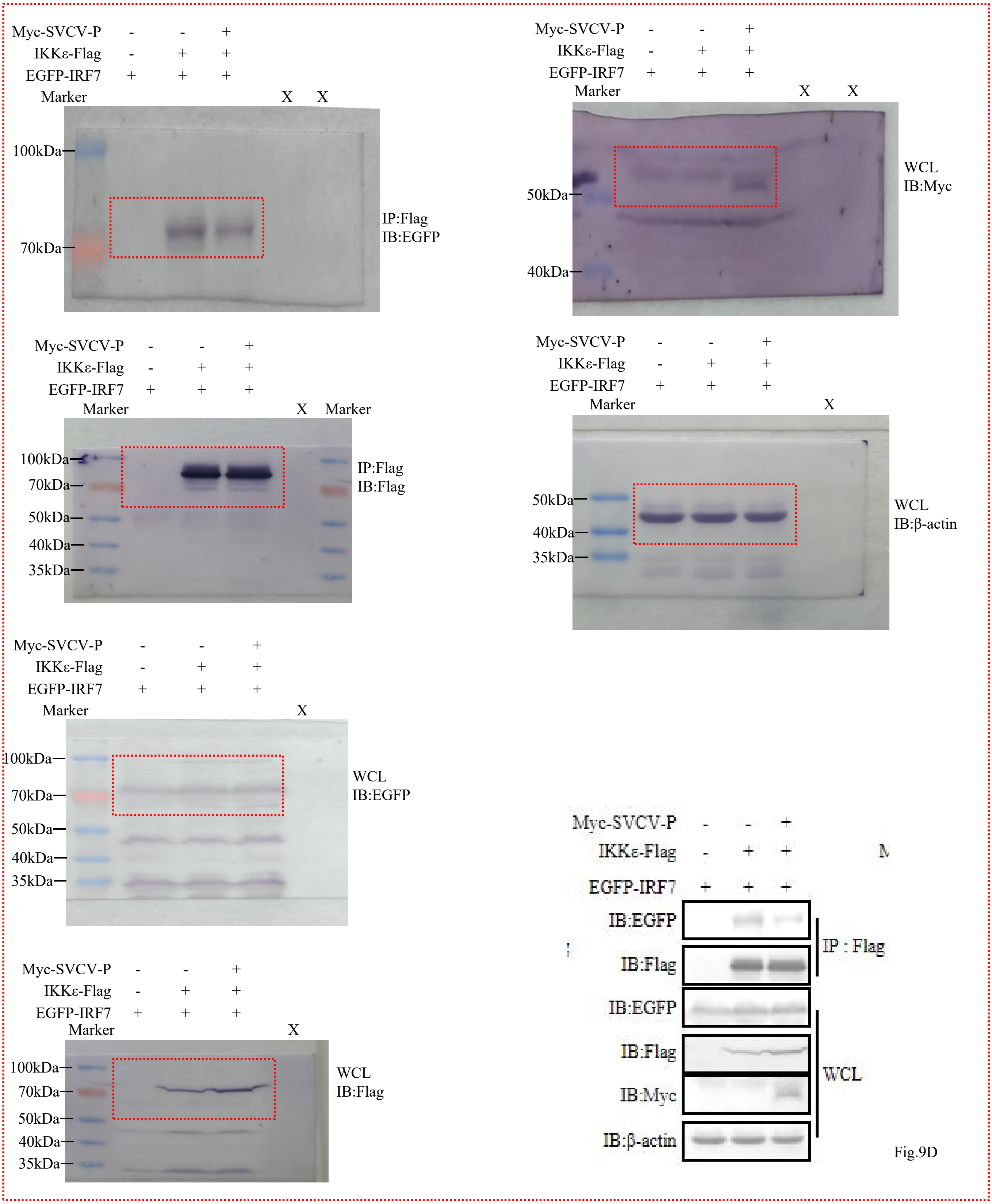
Myc-SVCV-P - - +
IKKε-Flag - + +
EGFP-IRF7 + + +
Marker
X X
100kDa
70kDa
IP:Flag
IB:EGFP
Myc-SVCV-P - - +
IKKε-Flag - + +
EGFP-IRF7 + + +
Marker
X X
50kDa
40kDa
WCL
IB:Myc
Myc-SVCV-P - - +
IKKε-Flag - + +
EGFP-IRF7 + + +
Marker
X
Marker
100kDa
70kDa
50kDa
40kDa
35kDa
IP:Flag
IB:Flag
Myc-SVCV-P - - +
IKKε-Flag - + +
EGFP-IRF7 + + +
Marker
X
50kDa
40kDa
35kDa
WCL
IB:β-actin
Myc-SVCV-P - - +
IKKε-Flag - + +
EGFP-IRF7 + + +
Marker
X
100kDa
70kDa
50kDa
40kDa
35kDa
WCL
IB:EGFP
Myc-SVCV-P - - +
IKKε-Flag - + +
EGFP-IRF7 + + +
Marker
X
100kDa
70kDa
50kDa
40kDa
35kDa
WCL
IB:Flag
Myc-SVCV-P - - +
IKKε-Flag - + +
EGFP-IRF7 + + +
IB:EGFP
IB:Flag
IP : Flag
IB:EGFP
IB:Flag
IB:Myc
IB:β-actin
WCL
Fig.9D

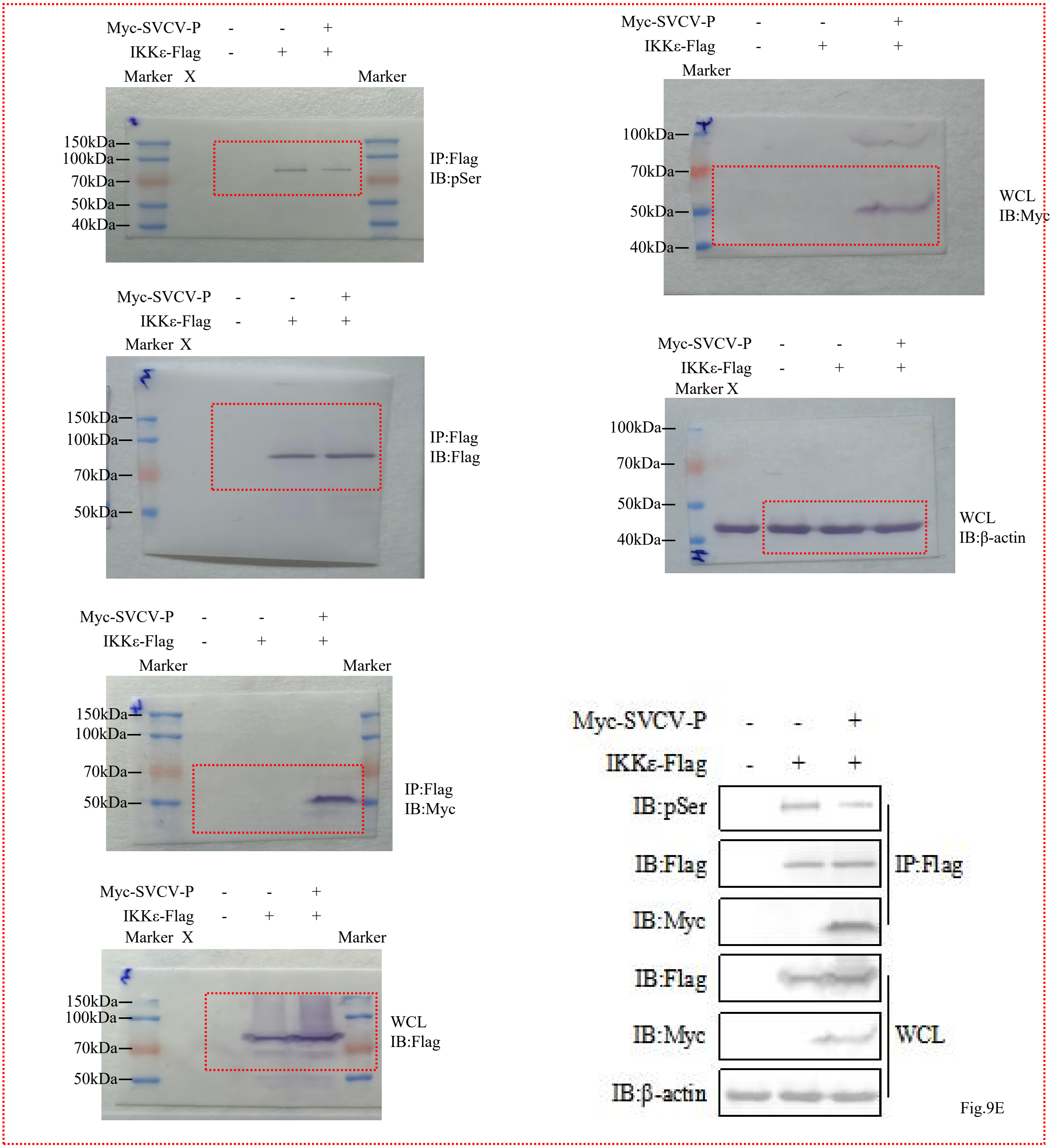

Myc-SVCV-P - - +
IKKε-Flag - + +
Marker X
Marker
150kDa
100kDa
70kDa
50kDa
40kDa
IP:Flag
IB:pSer
IB:Flag
IB:Myc
WCL
IB:Myc
IB:Flag
IB:β-actin
IB:pSer
IB:Flag
IB:Myc
IB:Flag
IB:Myc
IB:β-actin
IP:Flag
WCL

Fig.9E

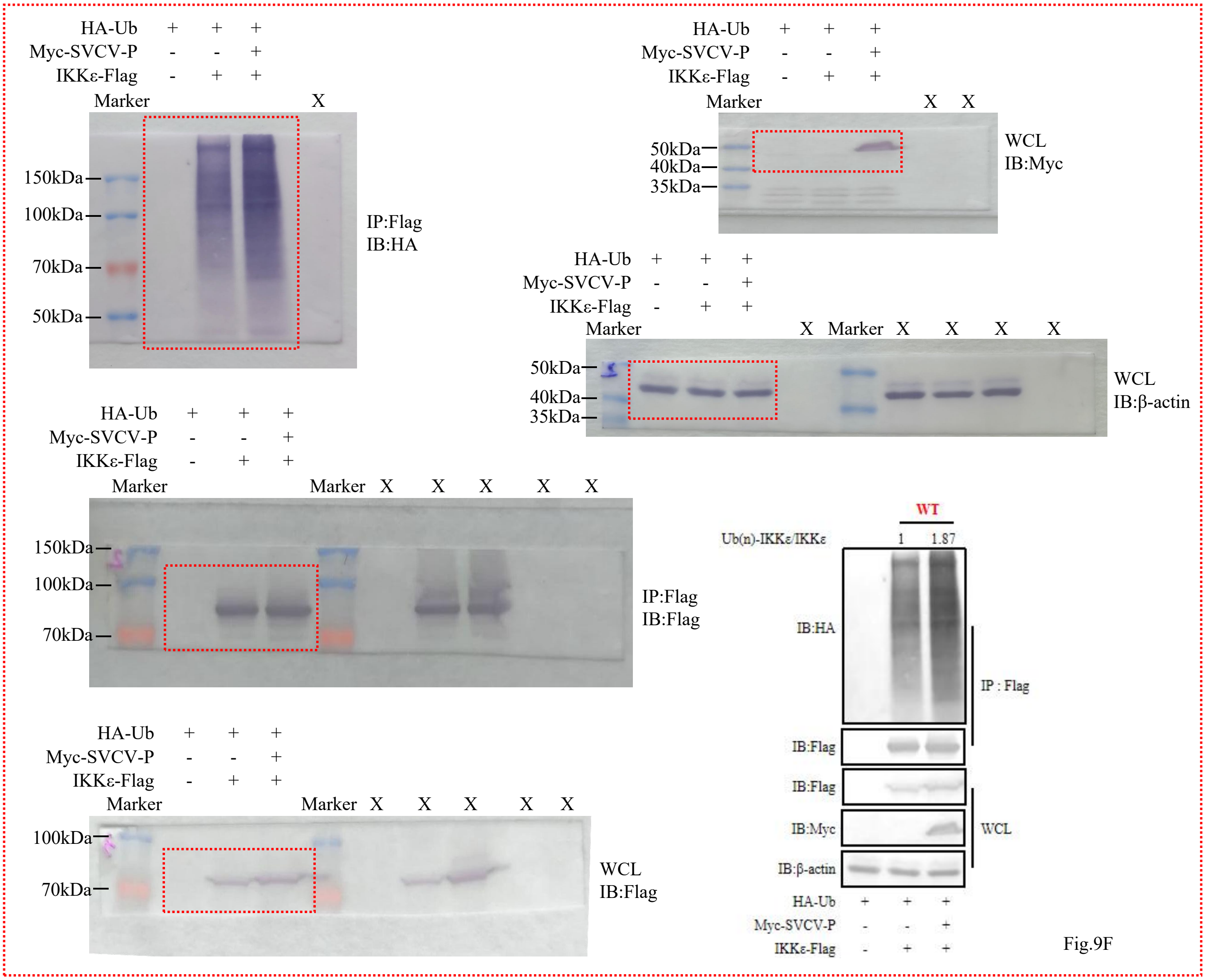
HA-Ub + + +
Myc-SVCV-P - - +
IKKε-Flag - + +
Marker X
150kDa
100kDa
70kDa
50kDa
IP:Flag
IB:HA
HA-Ub + + +
Myc-SVCV-P - - +
IKKε-Flag - + +
Marker X X
50kDa
40kDa
35kDa
WCL
IB:Myc
HA-Ub + + +
Myc-SVCV-P - - +
IKKε-Flag - + +
Marker X Marker X X X X
50kDa
40kDa
35kDa
WCL
IB:β-actin
HA-Ub + + +
Myc-SVCV-P - - +
IKKε-Flag - + +
Marker Marker X X X X X
150kDa
100kDa
70kDa
IP:Flag
IB:Flag
WT
Ub(n)-IKKε/IKKε 1 1.87
IB:HA
IP : Flag
IB:Flag
IB:Flag
IB:Myc
WCL
IB:β-actin
HA-Ub + + +
Myc-SVCV-P - - +
IKKε-Flag - + +
HA-Ub + + +
Myc-SVCV-P - - +
IKKε-Flag - + +
Marker Marker X X X X X
100kDa
70kDa
WCL
IB:Flag

Fig.9F

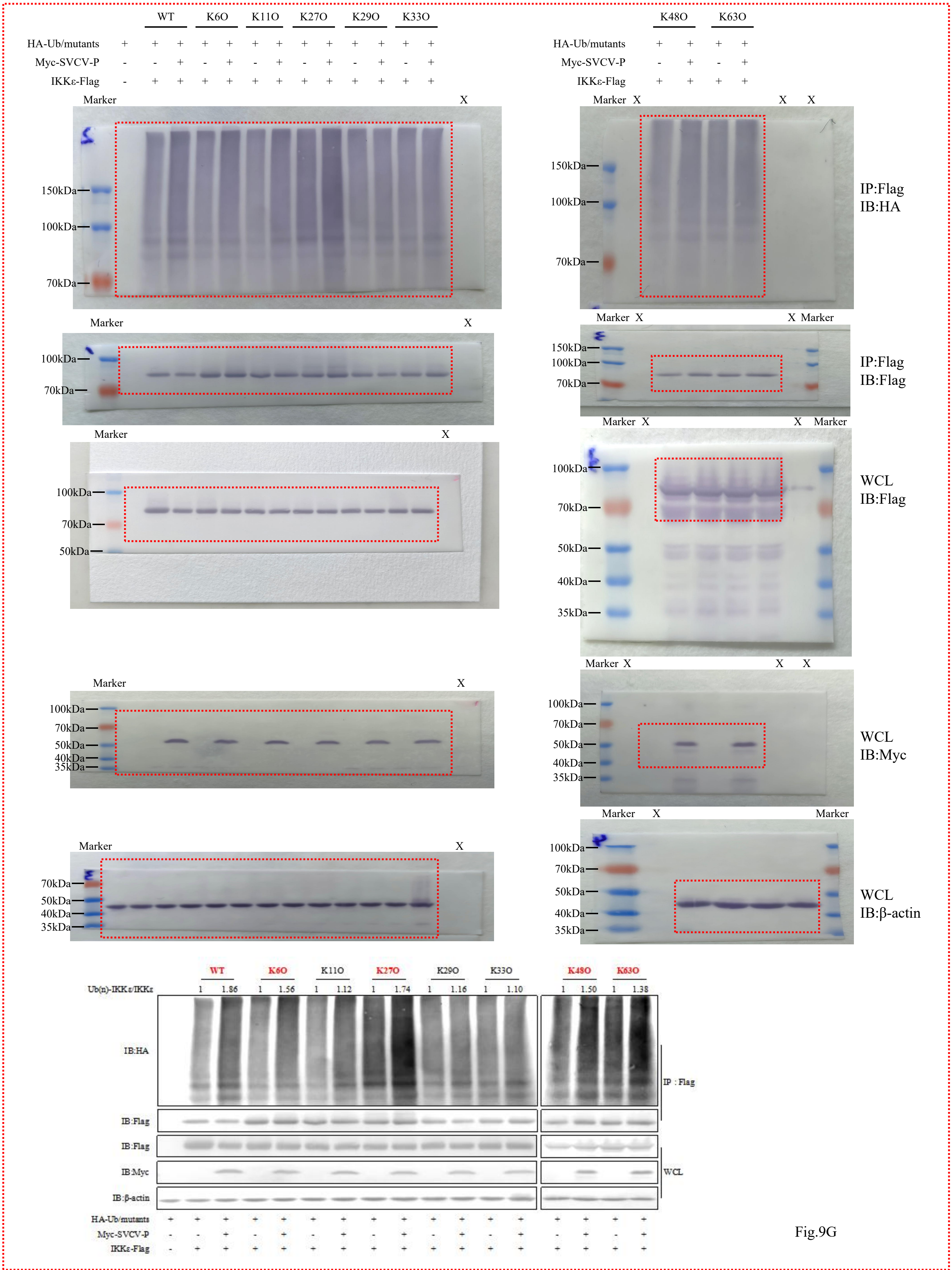
WT
K6O
K11O
K27O
K29O
K33O
K48O
K63O
HA-Ub/mutants
Myc-SVCV-P
IKKε-Flag
Marker
X
150kDa
100kDa
70kDa
50kDa
40kDa
35kDa
IP:Flag
IB:HA
IP:Flag
IB:Flag
WCL
IB:Flag
WCL
IB:Myc
WCL
IB:β-actin
Ub(n)-IKKε/IKKε
1 1.86 1 1.56 1 1.12 1 1.74 1 1.16 1 1.10 1 1.50 1 1.38
IB:HA
IB:Flag
IB:Flag
IB:Myc
IB:β-actin
IP : Flag
WCL

Fig.9G

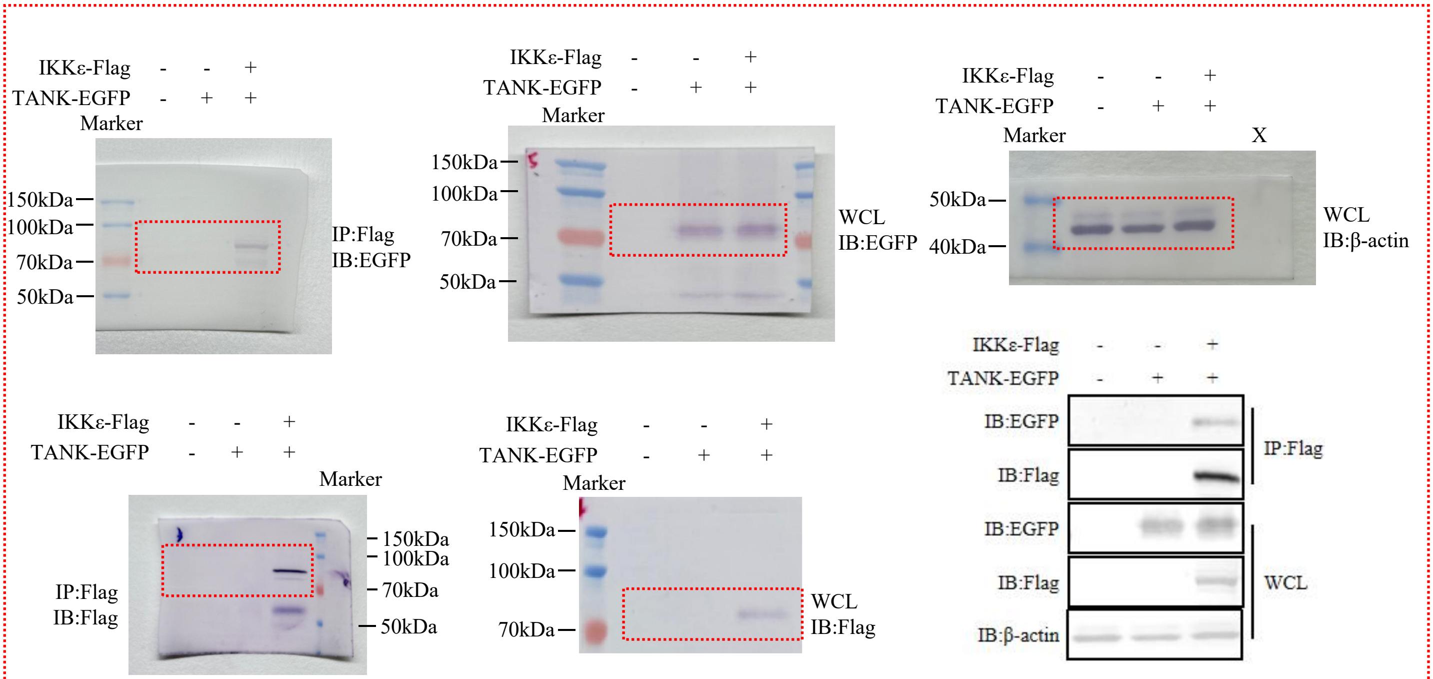

Supplementary Fig.3A

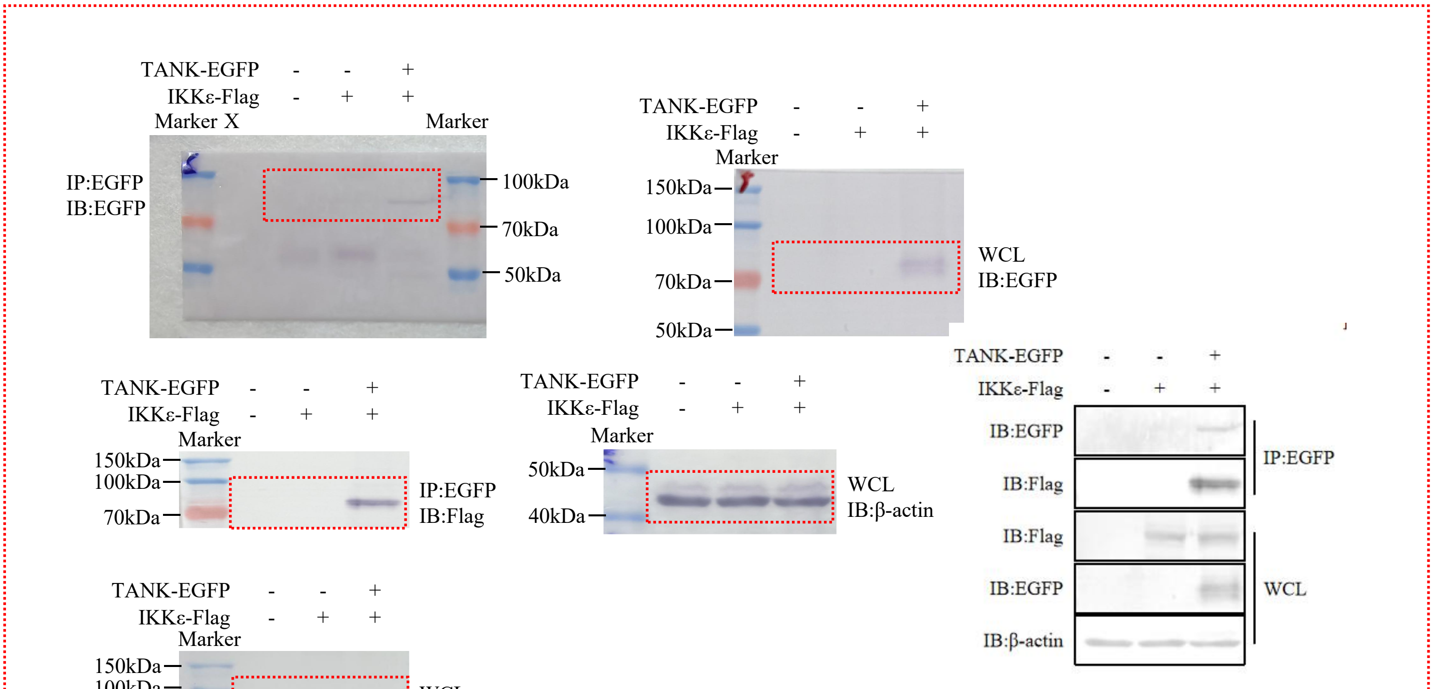

Supplementary Fig.3B

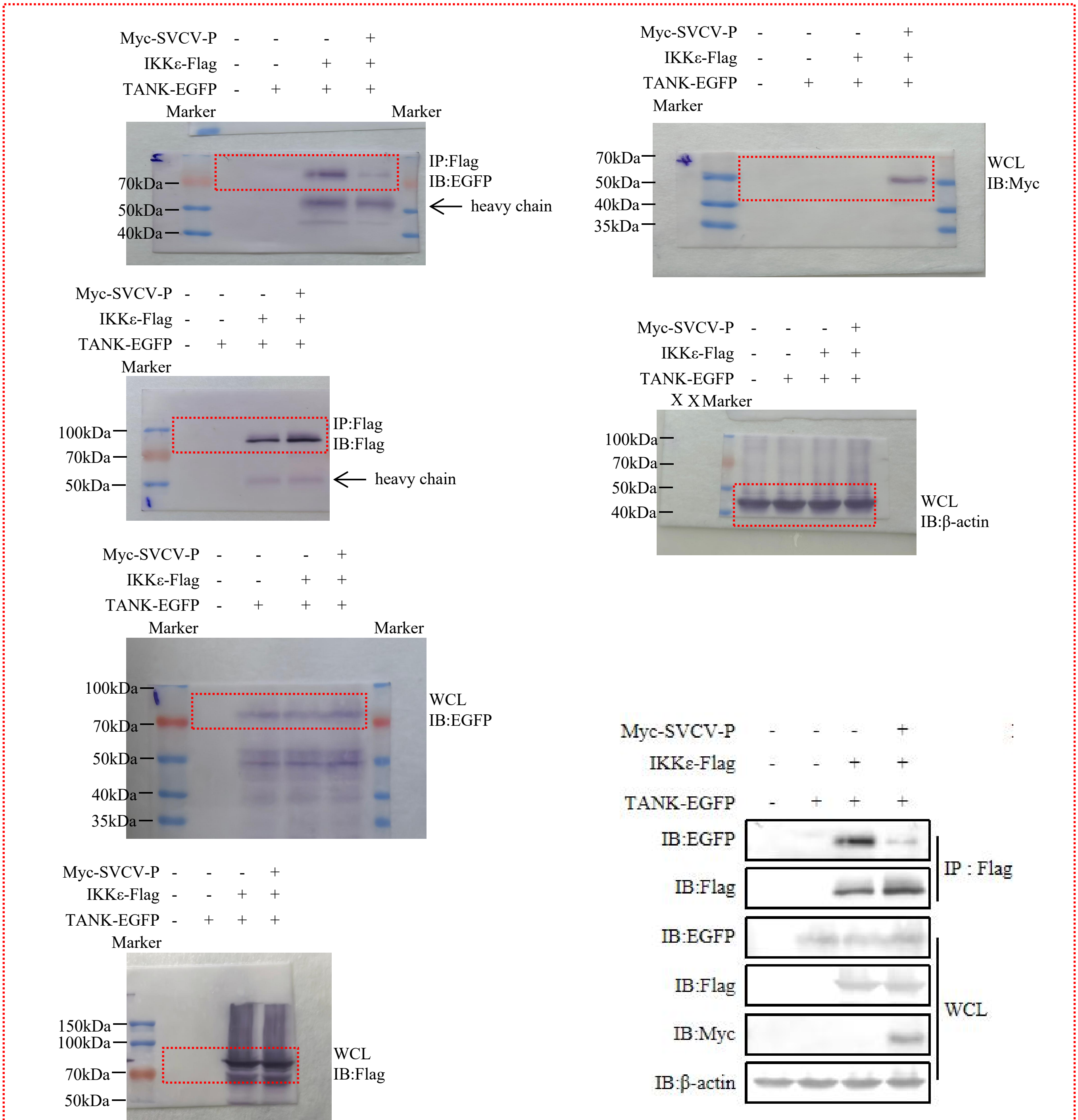

Supplementary Fig.5A

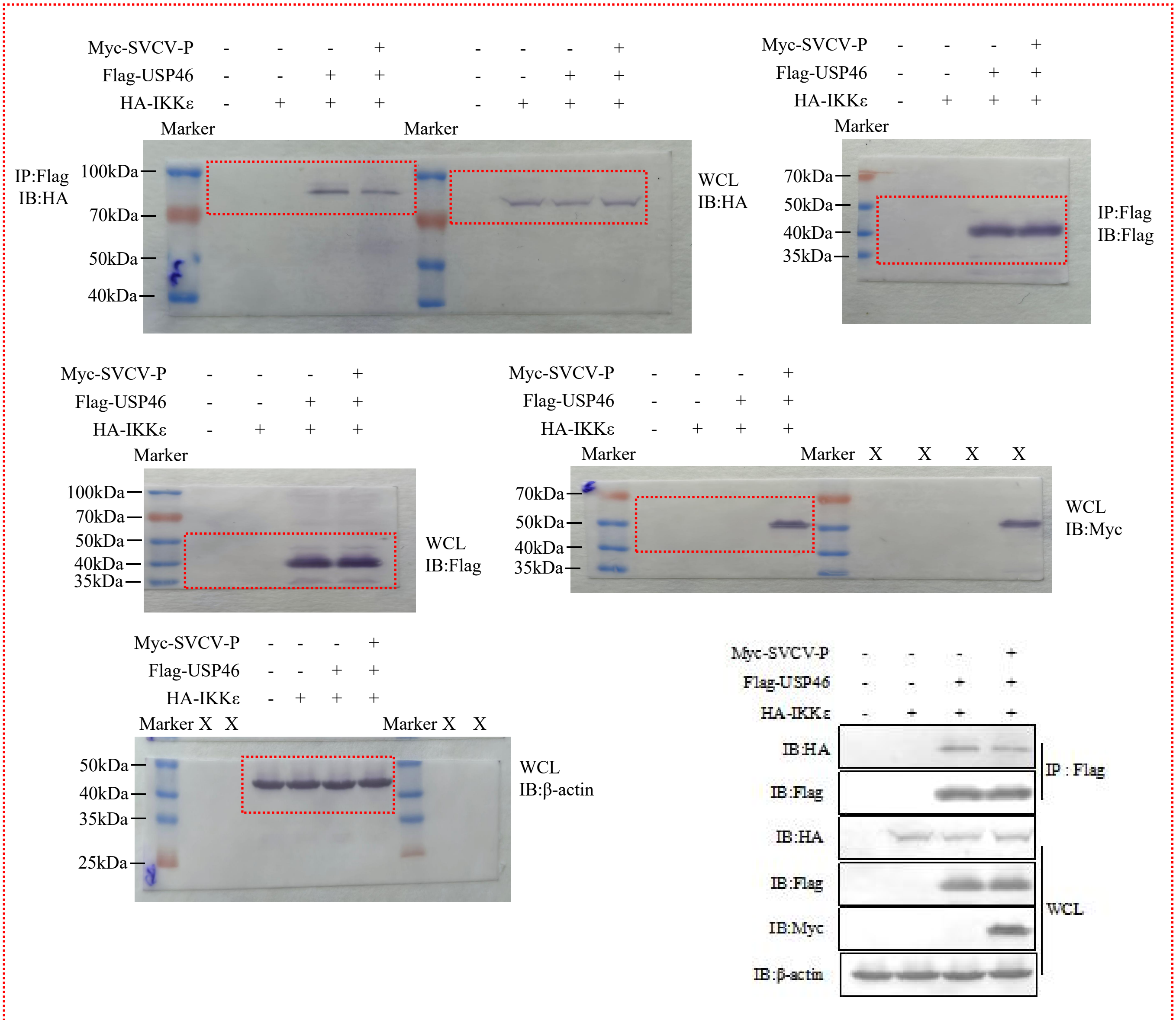

Supplementary Fig.5B
